# Cross‐sectional interactive associations of physical activity and sedentary behaviour with physical capacity across adulthood

**DOI:** 10.1002/jcsm.13457

**Published:** 2024-04-18

**Authors:** Jérémy Raffin, Yves Rolland, Mylène Aubertin‐Leheudre, Jaqueline Aragoni da Silva, Sophie Guyonnet, Fabien Pillard, Bruno Vellas, Philipe de Souto Barreto, for the INSPIRE group

**Affiliations:** ^1^ Institut Hospitalo‐Universitaire (IHU) HealthAge Toulouse France; ^2^ Institut du Vieillissement, Gérontopôle de Toulouse Centre Hospitalo‐Universitaire de Toulouse Toulouse France; ^3^ CERPOP UMR 1295, University of Toulouse III, Inserm, UPS Toulouse France; ^4^ Département des Sciences de l'activité physique, Faculté des sciences Université du Québec à Montréal Montréal Canada; ^5^ Centre de recherche, Institut universitaire de gériatrie de Montréal (IUGM), CIUSSS du Centre‐Sud‐de‐l'Île‐de‐Montréal Montréal Canada; ^6^ Unité de Médecine du Sport, Clinique Universitaire du Sport, Hôpital Pierre Paul RIQUET (Centre Hospitalo‐Universitaire) Toulouse France; ^7^ Centre RESTORE (Geroscience and Rejuvenation Center), UMR 1301 (INSERM)/UMR 5070 (CNRS) Toulouse France

**Keywords:** aging, exercise, lifestyle habits, physical activity, physical function, sedentary behaviour

## Abstract

**Background:**

The way physical activity (PA) and sedentary behaviour (SB) independently and interactively modify the age‐related decline in physical capacity remains poorly understood. This cross‐sectional study investigated the independent and interactive associations of PA and SB with physical function and performance throughout the adult life course.

**Methods:**

Data from 499 community‐dwelling adults (63% female) aged 20–92 years, involved in the INSPIRE Human Translational Cohort, were used in this cross‐sectional study. Daily time spent on moderate‐to‐vigorous PA (MVPA, min/day) and SB (h/day) was measured with activPAL triaxial accelerometers. Physical function and performance were assessed through the measurement of the 4‐m usual gait speed (m/s), handgrip strength (kg), lower‐limb strength (isokinetic knee extension torque, N·m), estimated lower‐limb power (five‐time chair‐rise test performance, s) and cardiorespiratory fitness (V̇O_2_max, mL/kg/min). Confounder‐adjusted multiple linear and curvilinear regressions were performed to investigate how MVPA, SB and their interactions were associated with the physical outcomes (all square root‐transformed except gait speed) throughout the adulthood spectrum.

**Results:**

Interaction analyses revealed that the combination of higher levels of MVPA with lower levels of SB favourably reshaped the negative relationship between handgrip strength and age (age^2^ × SB × MVPA: *B* = −7E‐08, SE = 3E‐08, *P* < 0.05). In addition, higher levels of MVPA were independently associated with an improved age‐related profile in gait speed (age^2^ × MVPA: *B* = 3E‐06, SE = 1E‐06, *P* < 0.05), chair‐rise performance (age × MVPA: *B* = −9E‐05, SE = 4E‐05, *P* < 0.05) and V̇O_2_max (MVPA at 21 years: *B* = 3E‐02, SE = 7E‐03, *P* < 0.05; age × MVPA: *B* = −5E‐04, SE = 2E‐04, *P* < 0.05). Conversely, the detrimental association of age with lower‐limb muscle strength (age × SB: *B* = −1E‐04, SE = 6E‐05, *P* < 0.05) and chair‐rise performance (age × SB: *B* = 1E‐05, SE = 7E‐06, *P* < 0.05) was exacerbated with increasing duration of SB, independently of MVPA. Supplementary analyses further revealed that some of these associations were age and sex specific.

**Conclusions:**

This cross‐sectional study demonstrated that reduced sedentary time and increased activity duration were independently and synergistically associated with an attenuated age‐related loss in physical capacity. These findings need to be confirmed with longitudinal data but encourage both adopting an active lifestyle and reducing sedentary time as preventive measures against physical aging.

## Introduction

Physical activity (PA), defined as any bodily movement produced by the skeletal muscles that requires an energy expenditure of more than 1.5 metabolic equivalents of task (METs),^1^ is an important modifiable factor that may attenuate the loss of physical function and performance related to aging.^2^ Over the past decades, numerous studies conducted in young (<40 years), middle‐aged (40–59 years) or older adults (60+) demonstrated that higher levels of PA were associated with better aerobic fitness^3^, ^4^, ^5^, ^6^, ^7^, ^8^, ^9^ and faster gait speed.^6^, ^10^, ^11^, ^12^, ^13^, ^14^, ^15^, ^16^, ^17^, ^18^, ^19^ However, investigations conducted on muscle power,^6^, ^10^, ^12^, ^13^, ^18^, ^20^, ^21^ lower‐limb^6^, ^12^, ^22^, ^23^, ^24^ and handgrip^17^, ^18^, ^20^, ^22^, ^24^, ^25^, ^26^ muscle strength provided mixed results.

These discrepancies may be explained by the fact that all studies did not use the same tools for assessing PA levels (accelerometers vs. questionnaires), included populations from different age ranges or did not control for sedentary behaviour (SB; which is defined as any waking behaviour spent sitting, lying or reclining with an energy expenditure of 1.5 METs or less^27^). SB has progressively become the target of growing interest, but its relationship with physical function and performance remains poorly understood. Studies conducted in young adults reported lower cardiorespiratory fitness with increasing levels of SB.^8^ Studies involving middle‐aged and old adults consistently reported no associations between handgrip strength and SB,^17^, ^18^, ^24^, ^25^, ^28^ but mixed results regarding gait speed,^10^, ^12^, ^17^, ^18^, ^28^, ^29^, ^30^ lower‐limb muscle strength^12^, ^23^, ^24^ and estimated lower‐limb muscle power.^9^, ^10^, ^12^, ^13^, ^18^, ^20^, ^24^, ^29^, ^30^, ^31^ Other studies, including subjects from the whole adult lifespan, indicated that the levels of SB were negatively associated with lower‐limb muscle strength,^22^ but not with aerobic fitness.^5^

In addition to having independent effects, PA and SB may also interact with each other to modulate physical function and performance in a more complex manner. Yet, only a few studies have examined how these interactions may be associated with physical function across adulthood. In older adults, it has been shown that achieving at least 36 min of moderate‐to‐vigorous PA (MVPA) per day dampened the negative relationship between sitting time and physical function.^32^ In middle‐aged and older adults, it was shown that MVPA moderates the effect of SB on gait speed.^10^ Further, in adults aged 51 and over, combining at least 7 min/day of MVPA and <12.4 h/day of SB was associated with better muscle power compared with having an opposite MVPA/SB pattern.^10^ Likewise, better V̇O_2_max was reported in young adults who associated high MVPA (highest 20%) with low SB (lowest 20%) levels, compared with those with a reversed MVPA/SB combination.^8^ Other studies investigating whether this interaction was associated with gait speed,^33^ handgrip strength,^33^ muscle power^33^ and lower‐limb muscle strength^23^ reported inconclusive results in middle‐aged and older subjects.

Therefore, the literature regarding the role of PA and SB in modulating the age‐associated decline in physical function and performance is not consistent, and researchers have highlighted the need for considering both PA and SB in statistical models.^10^ Comprehensive studies including large populations with ages covering the full spectrum of adulthood, with objectively measured PA and SB, and exploring both their independent and interactive associations with physical function and performance are lacking. In this work, we investigated the independent and interacting associations of age, MVPA and SB with physical functions across the adult lifespan in a large cohort of subjects aged 20–92. We hypothesized that (1) PA and SB would interact with each other to modify the relationship between age and physical health. Further, we expected that (2) higher levels of MVPA would independently reduce the age‐related differences in physical function and performance, while (3) higher levels of SB would have the opposite effect.

## Methods

The present cross‐sectional study was conducted in the context of the INSPIRE research program,^S1,S2^ a geroscience initiative that was designed to fulfil two main objectives: (1) identifying the biomarkers of aging using both animal and human cohorts (the INSPIRE Human Translational Cohort and the INSPIRE Animal Cohort) and (2) implementing the Integrated Care for Older People (ICOPE) recommendations provided by the World Health Organization (WHO) into the clinical care of older adults in order to monitor and prevent the decline in intrinsic capacity. The protocol of the INSPIRE Human Translational Cohort was conducted in accordance with the 1964 Declaration of Helsinki and registered on http://clinicaltrials.gov (ID NCT04224038). It was approved by the French Ethics Committee located in Rennes (CPP Ouest V) in October 2019. The French ‘National Commission for Data Protection’ gave its authorization on 13 April 2017 (Ref. Nb. MMS/OSS/NDT171027). All participants provided their written consent.

### Study population

This research used the baseline data collected in 2019–2021 from the INSPIRE Human Translational Cohort. INSPIRE is a 10‐year observational longitudinal study that started in October 2019 and involves 1014 voluntary community‐dwelling participants recruited in the south‐western area of France.^S1^ Men and women aged 20 and over (no upper limit regarding age) and affiliated with the French social security system were included, with no further eligibility criteria. In order to have a representative sample of the overall population, people suffering from any conditions, including those that may reduce physical capacity, such as cardiovascular diseases, pulmonary diseases, metabolic diseases, osteoarthritis or cancer, were not excluded unless the conditions restricted their life expectancy at 5 years (or at 1 year for disabled older adults). Individuals dispossessed of their liberty for judicial or administrative reasons or under guardianship were not included. The present study was restricted to the subset of participants who agreed to wear accelerometers, which represents a total of 499 subjects with valid data.

### Assessment of physical function and performance

Physical function was assessed by measuring usual gait speed (4‐m walking test) as an indicator of locomotion^S3^ and handgrip strength as an indicator of overall muscle strength.^S4^ Physical performance was assessed using a five‐time chair‐rise test, which estimated lower‐limb muscle power^S3^; a V̇O_2_max test, which assessed cardiorespiratory fitness^S5^; and a lower‐limb isokinetic muscle strength test,^S6–S10^ which assessed lower‐limb dynamic muscle strength. Normative values have been published for gait speed,^S11–S13^ handgrip strength,^S14–S16^ five‐time chair‐rise test,^S14,S17^ lower‐limb isokinetic strength^S18^ and V̇O_2_max^S19,S20^ in adult populations from different ages, covering the whole adulthood spectrum. Data on gait speed, handgrip strength and five‐time chair‐rise tests were collected from all INSPIRE participants. However, data on V̇O_2_max and lower‐limb isokinetic muscle strength were collected on a voluntary basis in 245 and 252 subjects among the INSPIRE participants. A detailed description of the physical function and performance tests can be found in the supporting information.

### Physical activity and sedentary behaviour assessment

ActivPAL accelerometers were used as valid and reliable tools for measuring the levels of MVPA and SB.^34^, ^35^ The participants wore the accelerometer on their thighs for 7 days and were instructed not to change their lifestyle habits throughout this period. PA and SB data were then extracted using the software PAL Analysis and processed with the R package and the PAL Event Analysis Excel macro. The mean of the daily duration spent stepping at a cadence ≥ 100 steps per minute^36^ and time spent cycling were computed for each individual to define the daily average duration of MVPA (min/day). Similarly, the mean of the daily duration of waking time spent sitting or lying was calculated in order to obtain the daily average duration of SB (min/day and converted into h/day for descriptive purposes). The average daily number of sit‐to‐stand transitions was also considered for analysis. Days with 4 h or more of non‐wear time were excluded from the analyses.^37^ Participants who did not have accelerometer data for at least 4 days, including one weekend day, were also excluded from the analyses.^37^

### Confounders

All models were adjusted for sex, cardiovascular disease (defined as having at least one history of stroke, ischaemic heart disease, heart failure or peripheral vascular disease), hypertension, hypercholesterolaemia, diabetes, chronic pulmonary disease, cancer, disabling osteoarthritis, depression, cognition (using the mini mental state examination), waist‐to‐hip ratio, number of drugs and economic status (using the total family income). The average daily number of sit‐to‐stand transitions was also included in the models in order to control for the number of SB breaks. Because anthropometric characteristics may affect physical function and performance, further adjustments for body weight and body height were performed for gait speed, handgrip strength and chair‐rise test. Isokinetic muscle strength was also adjusted for body weight, and gait speed was further controlled for gait aid.

### Statistical analyses

Descriptive statistics were calculated as medians with first and third quartiles for continuous variables and numbers with percentages for categorical variables. Multiple linear regressions were performed in order to investigate the independent and interacting associations of PA (continuous) and SB (continuous) with physical outcomes across the lifespan, using age as a continuous predictor. Given that aging is not a linear process and that functional decline accelerates with age,^S11,S21,S22^ which was further suggested by a preliminary visual inspection of our data, we considered the possibility of running curvilinear models using second‐order polynomial regressions. Therefore, for each dependent variable, we first compared linear versus polynomial models using unadjusted regressions. To do so, we ran for each outcome two unadjusted models, one including only age as a predictor (linear model) and one including the term age^2^ in addition to age (polynomial model). These two models were compared using an *F* test, and the polynomial model was retained for the analyses only if the *F* test was significant.^S23^ It turned out that polynomial models were chosen for gait speed and handgrip strength, while linear models were selected for the other outcomes. After the models were chosen, confounder‐adjusted analyses were performed, using PA and SB as moderators interacting with age and age^2^ in order to test Hypotheses 2 and 3. The three‐way interactions of age × PA × SB and age^2^ × PA × SB were also included in the models in order to test our Hypothesis 1. In addition, customized contrast tests were performed to obtain, for each dependent variable, the age ranges for which age, PA, SB and their interactions had a significant effect. The effects of the single terms (age, MVPA and SB) and second‐order interaction terms (age × MVPA and age × SB) were computed while fixing any moderators (MVPA, SB or both) at their median levels. It should be noted that the term ‘effect’ was used throughout this manuscript as a statistical term that does not imply causal relationships. For each regression model, the normal distribution and the homoscedasticity of the residuals were checked visually. If these assumptions were violated, data transformation was performed, which led to the square root transformation of all the dependent variables except gait speed. Estimated marginal means were also computed from the models in order to present estimated values of each physical outcome across the lifespan. These estimations were provided for the overall sample and for different levels of MVPA and SB. The analyses were also supported by figures that illustrated the relationships examined herein. In addition, sensitivity analyses were performed for V̇O_2_max and isokinetic strength while removing individuals with cardiovascular disease. Moreover, supplementary analyses were conducted for all the outcomes while stratifying the whole sample by sex. All statistical analyses were carried out with the software packages SPSS statistics and R, with a significance threshold set at *P* < 0.05.

## Results

### Characteristics of the participants

Among the 499 participants included in the present study, all of them had valid measures of gait speed and handgrip strength, while 496, 165 and 168 subjects had valid data for the chair‐rise test, V̇O_2_max and lower‐limb isokinetic strength, respectively. The included subjects were aged 20–92 years (median = 63 years), with 63.1% being women. Ninety‐seven per cent (*n* = 486/499) of the participants wore the accelerometers for at least 6 days, and 99.6% (*n* = 497/499) of the subjects wore the device during the whole weekend. The median MVPA and SB levels were 21.4 (Q1–Q3 = 10.4–37) min/day and 6.5 (Q1–Q3 = 5.4–7.7) h/day, respectively. Further characteristics of physical capacity, health status and socio‐economic data are provided in *Table* *1*. Stratified characteristics according to each decade of age and sex categories are available in *Tables* *S1a*–*S1c*.

**Table 1:** Characteristics of the participants

Variable (unit)	Total		
	Sample size	Statistic	Min–max
Age (years)	499	63 (45–74)	20–92
Female	499	315 (63.1%)	
Education	499	499 (100%)	
No education		1 (0.2%)	
Primary school certificate		19 (3.8%)	
Secondary education diploma		36 (7.2%)	
High school diploma		70 (14%)	
University degree		373 (74.7%)	
Socio‐professional category	499	499 (100%)	
Farmers		5 (1%)	
Artisans, shopkeepers and CEOs		25 (5%)	
Executives and intellectual professionals		176 (35.3%)	
Intermediate professions		164 (32.9%)	
Employees		76 (15.2%)	
Workers		4 (0.8%)	
Unemployed		6 (1.2%)	
Others		43 (8.6%)	
Family income (€)	496	496 (100%)	
<1000–1500		45 (9.1%)	
1500–2800		136 (27.4%)	
2800–4200+		292 (58.9%)	
Cannot or do not wish to answer		23 (4.6%)	
Height (m)	499	1.7 (1.6–1.7)	1.3–2
Body weight (kg)	498	66 (58–76.5)	39–142
Body mass index (kg/m^2^)	498	24.1 (21.7–27)	15.6–40.4
Waist‐to‐hip ratio	499	0.9 (0.8–0.9)	0.6–1.3
Heart rate (b.p.m.)	498	67 (60–74)	38–100
Systolic blood pressure (mmHg)	498	127 (115–142)	84–217
Diastolic blood pressure (mmHg)	498	72 (64–79)	50–112
Number of drugs (*n*)	499	1 (1–3)	1–15
Cardiovascular disease	499	72 (14.4%)	
Chronic pulmonary disease	499	34 (6.8%)	
Cancer	499	43 (8.6%)	
Diabetes	499	14 (2.8%)	
Hypertension	499	124 (24.8%)	
Hypercholesterolaemia	499	83 (16.6%)	
Depression	499	72 (14.4%)	
Osteoarthritis	499	40 (8%)	
Walking aid	491	12 (2.4%)	
MVPA (min/day)	499	21.4 (10.4–37)	0–127.9
SB (min/day)	499	391.1 (321.7–464.5)	110.4–809.6
Gait speed (m/s)	499	1.3 (1.1–1.4)	0.4–2.1
Men	184	1.3 (1.1–1.4)	0.5–2.1
Women	315	1.3 (1.1–1.4)	0.4–1.9
Handgrip strength (kg)	499	32 (26–40)	6–68
Men	184	42 (34–49.5)	19–68
Women	315	28 (24–33)	6–49
Five‐time sit to stand (*n*)	496	8.9 (7–10)	3.9–17
Men	182	9 (7.2–10)	3.9–16.1
Women	314	8.4 (7–10)	4–17
V̇O_2_max (mL/kg/min)	165	23 (20–30)	13–57
Men	69	27 (22–34)	13–57
Women	96	22 (19–26)	14–42
Isokinetic strength (kg)	168	97 (71–121)	29–244
Men	73	119 (97–142)	52–244
Women	95	82 (62–101)	29–177
Mini mental state examination score	498	29 (28–30)	16–30
Short physical performance battery score	494	12 (12–12)	2–12

*Note*: Continuous variables are presented as median (Q1–Q3) along with minimum and maximum values. Categorical variables are presented as *n* (%). Abbreviations: MVPA, moderate‐to‐vigorous physical activity; SB, sedentary behaviour.

### Independent associations with moderate‐to‐vigorous physical activity

**Figure 1:**
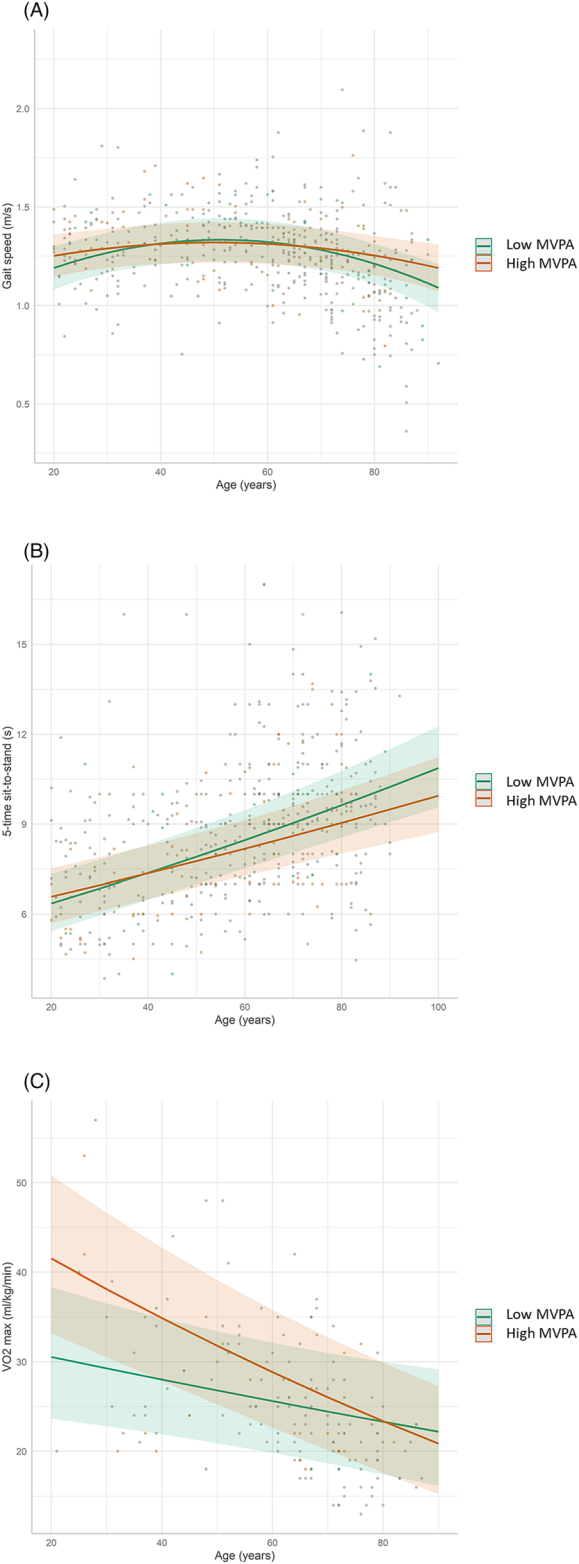
Associations between age and physical function and performance according to the levels of moderate‐to‐vigorous physical activity (MVPA). The graph indicates that there was a significant interaction between age and MVPA such that higher levels of MVPA were associated with a more favourable age‐related relationship in gait speed (A), chair‐rise performance (B) and V̇O_2_max (C), independently of sedentary behaviour levels. For illustrative purposes, low and high MVPA levels were defined using the first and third quartile values of the distribution, which roughly equal 10 and 37 min/day, respectively.

We detected a significant effect of MVPA and its interaction with age and age^2^, revealing that higher MVPA levels were associated with a better age‐related profile in gait speed, chair‐rise performance and V̇O_2_max. Notably, MVPA was positively associated with gait speed from 80 to 92 years, chair‐rise performance from 58 to 92 years and V̇O_2_max from 21 to 70 years. The age‐related differences in gait speed and chair‐rise performance were reduced with increasing levels of MVPA, while the opposite effect was observed for V̇O_2_max. The independent associations of the physical outcomes with MVPA and its interaction with age are reported in *Tables* *2* and *S2*. They are illustrated in *Figure* *1*. Estimated marginal mean values and their differences across the lifespan according to the levels of MVPA are given in *Table* *S5a*.

**Table 2:** Independent and interactive associations of age, moderate‐to‐vigorous physical activity and sedentary behaviour with physical function and performance across the adult lifespan

IV^a^	Gait speed (m/s)			Handgrip strength (square root of kg)			Five‐time chair rise (square root of s)			Isokinetic strength (square root of N·m)			V̇O_2_max (square root of mL/kg/min)		
	*N* = 486			*N* = 494			*N* = 491			*N* = 165			*N* = 162		
	*B*	SE	*P*	*B*	SE	*P*	*B*	SE	*P*	*B*	SE	*P*	*B*	SE	*P*
Age	7E‐03	2E‐03	**0.001**	4E‐03	6E‐03	0.477	9E‐03	1E‐03	**<0.001**	−4E‐02	1E‐02	**<0.001**	−2E‐02	3E‐03	**<0.001**
Age^2^	−1E‐04	3E‐05	**<0.001**	−2E‐04	9E‐05	**0.012**	—	—	—	—	—	—	—	—	—
MVPA	2E‐03	2E‐03	0.150	6E‐03	4E‐03	0.169	2E‐03	2E‐03	0.368	−5E‐03	2E‐02	0.788	3E‐02	7E‐03	**<0.001**
MVPA × age	−2E‐04	9E‐05	0.063	−2E‐04	3E‐04	0.402	−9E‐05	4E‐05	**0.043**	2E‐04	5E‐04	0.597	−5E‐04	2E‐04	**0.003**
MVPA × age^2^	3E‐06	1E‐06	**0.032**	2E‐06	3E‐06	0.656	—	—	—	—	—	—	—	—	—
SB	2E‐04	3E‐04	0.435	−1E‐03	7E‐04	0.088	−7E‐04	3E‐04	**0.035**	6E‐03	3E‐03	**0.050**	3E‐05	1E‐03	0.974
SB × age	−3E‐05	2E‐05	0.141	6E‐05	5E‐05	0.191	1E‐05	7E‐06	**0.045**	−1E‐04	6E‐05	**0.025**	−2E‐06	2E‐05	0.916
SB × age^2^	3E‐07	2E‐07	0.157	−7E‐07	7E‐07	0.258	—	—	—	—	—	—	—	—	—
MVPA × SB	4E‐06	2E‐05	0.786	−7E‐05	4E‐05	0.099	2E‐05	2E‐05	0.183	−2E‐04	2E‐04	0.279	−1E‐04	6E‐05	0.074
MVPA × SB × age	4E‐07	9E‐07	0.630	5E‐06	2E‐06	**0.048**	−7E‐07	4E‐07	0.075	4E‐06	4E‐06	0.337	2E‐06	2E‐06	0.118
MVPA × SB × age^2^	−9E‐09	1E‐08	0.407	−7E‐08	3E‐08	**0.024**	—	—	—	—	—	—	—	—	—

*Note*: Bold *P* values indicate statistical significance. Abbreviations: IV, independent variable; MVPA, moderate‐to‐vigorous physical activity (min/day); SB, sedentary behaviour (min/day); SE, standard error.

^a^ The effects of the single terms (age, MVPA and SB) and second‐order interaction terms (age × MVPA and age × SB) were computed while fixing any moderators (MVPA, SB or both) at their median levels. In addition, the effects of MVPA, SB and MVPA × SB were provided for the lower limit of age (20 for gait speed, handgrip strength and chair‐rise test and 21 for isokinetic strength and V̇O_2_max). The same limits were chosen to compute the effects of age, age × MVPA, age × SB and age × MVPA × SB in quadratic models (gait speed and handgrip strength).

### Independent associations with sedentary behaviour

**Figure 2:**
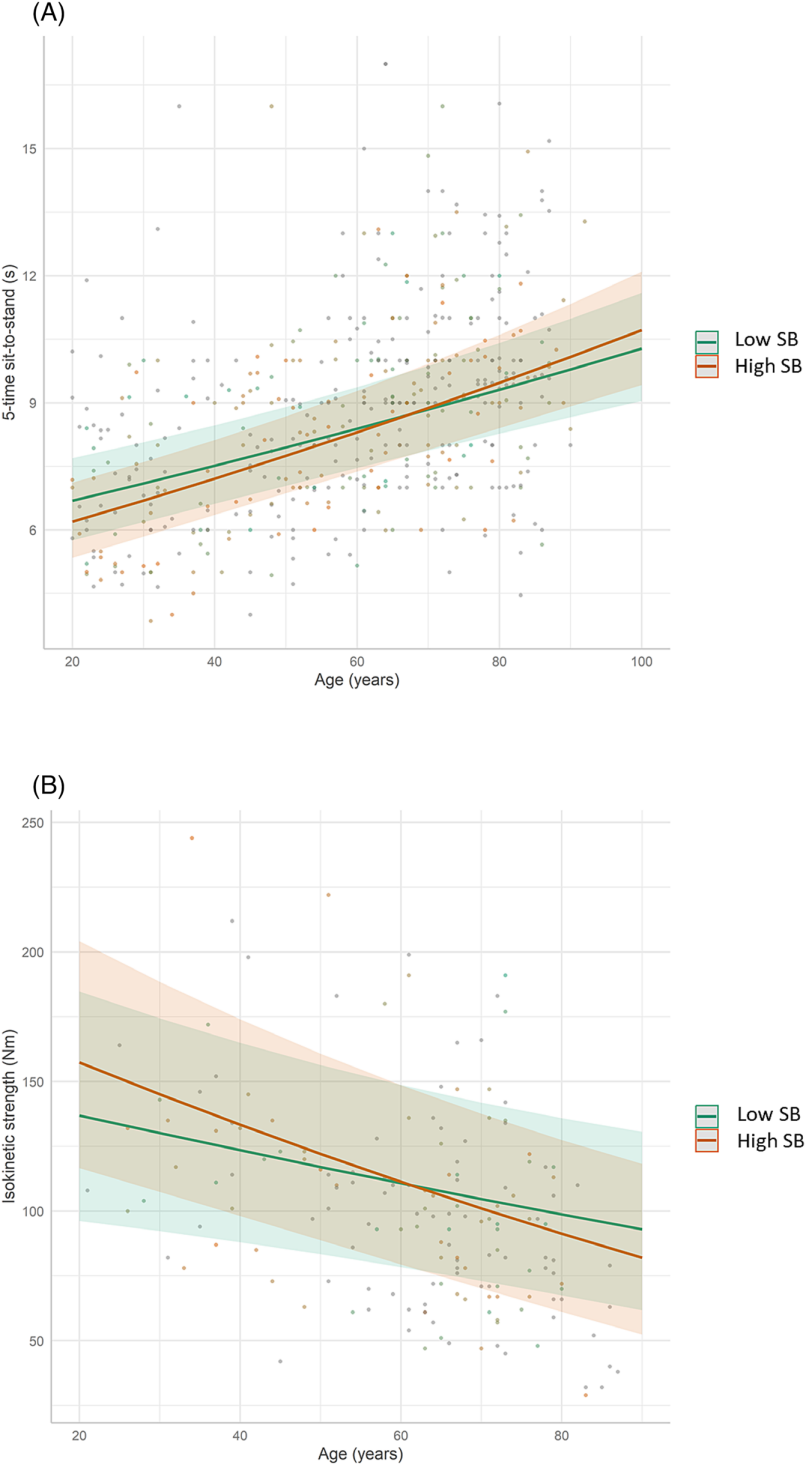
Associations between age and physical function and performance according to the levels of sedentary behaviour (SB). The graph indicates that there was a significant interaction between age and SB such that the detrimental associations of age with chair‐rise performance (A) and isokinetic strength (B) were more pronounced with increasing SB duration, independently of moderate‐to‐vigorous physical activity levels. For illustrative purposes, low and high SB levels were defined using the first and third quartile values of the distribution, which roughly equal 5.4 and 7.7 h/day, respectively.

Interaction analyses indicated that the deleterious association of age with lower‐limb muscle strength and chair‐rise performance was more pronounced with increasing levels of SB, independently of MVPA levels. More specifically, SB was negatively associated with gait speed in individuals aged 54–71 years. In contrast, SB levels were positively associated with chair‐rise performance in individuals aged 21–36 years. The independent association of the physical outcomes with SB and its interaction with age are reported in *Tables* *2* and *S2*. These findings are illustrated in *Figure* *2*. Estimated marginal mean values and their differences across the lifespan according to the levels of SB are given in *Table* *S6a*.

### Interaction between physical activity and sedentary behaviour

**Figure 3:**
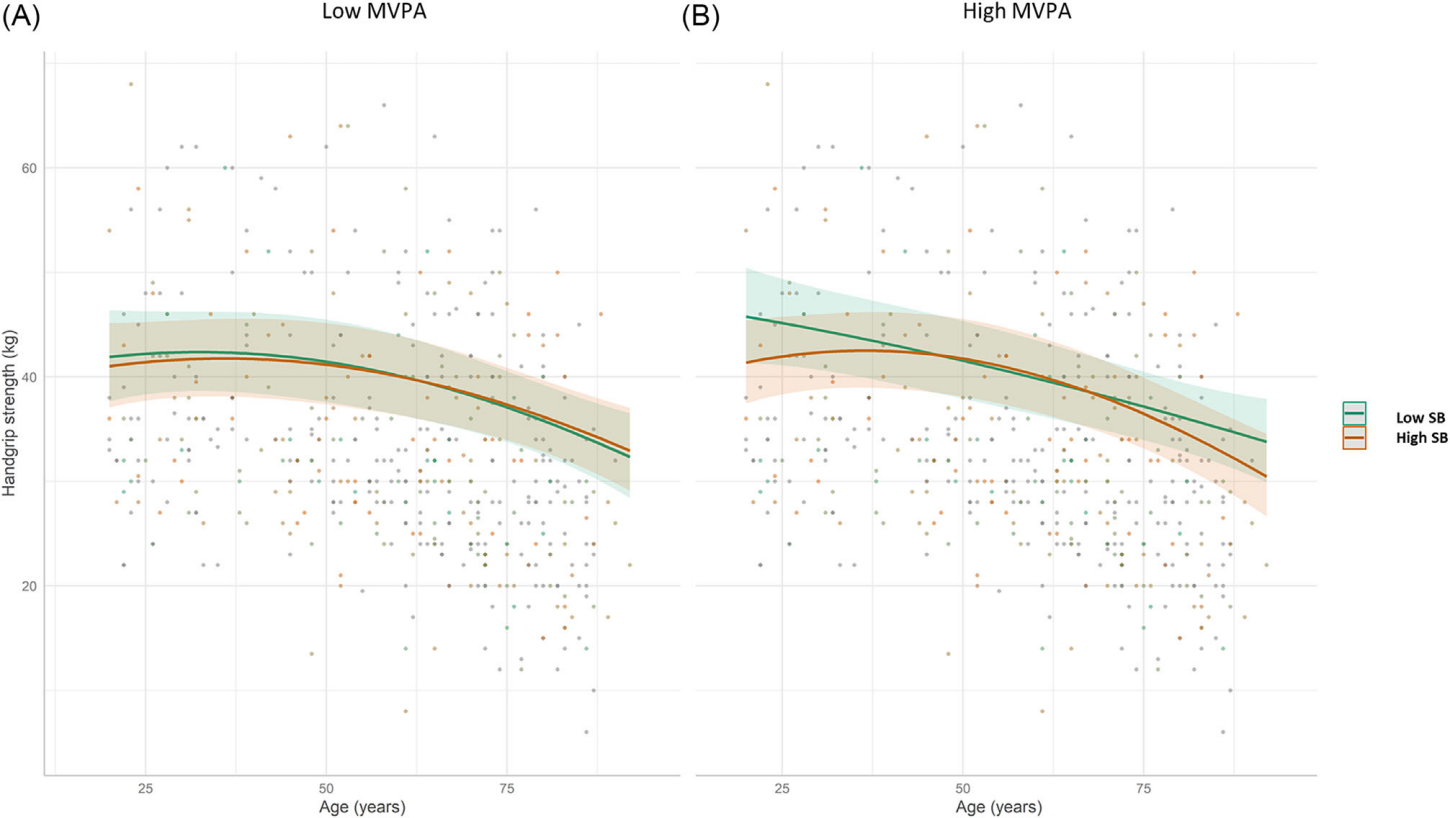
Associations between age and handgrip strength according to the levels of sedentary behaviour (SB) and moderate‐to‐vigorous physical activity (MVPA). Young and old individuals who combined high levels of MVPA with low levels of SB (“Low SB” category in panel B) had better handgrip strength than those who did not (panel A and “High SB” category in panel B). For illustrative purposes, low and high SB levels were defined using the first and third quartile values of the distribution, which roughly equal 5.4 and 7.7 h/day, respectively. Likewise, low and high MVPA levels were defined using the first and third quartile values of the distribution, which roughly equal 10 and 37 min/day, respectively.

Our analyses revealed significant interaction effects between age, age^2^, MVPA and SB on handgrip strength. As depicted in *Figure* *3*, we observed a synergistic effect of MVPA and SB, indicating that the age‐related decline in handgrip strength was reduced in older adults who combined high levels of MVPA with low levels of SB compared with those who had low MVPA levels and/or high sedentary time. The interacting effects of age, age^2^, SB and MVPA on the physical outcomes are reported in *Tables* *2* and *S2*. Estimated marginal mean values and their differences across the lifespan according to the levels of SB and MVPA are given in *Table* *S7a*.

### Sensitivity and supplementary analyses stratified by sex

Sensitivity analyses are reported in *Table* *S3a*. They indicated that after removing the individuals who suffered from cardiovascular diseases (15 and 14 subjects for isokinetic strength and V̇O_2_max, respectively), the associations regarding V̇O_2_max and isokinetic strength reported above remained significant.

After stratifying the analyses by sex, it appeared that the independent associations of MVPA with chair‐rise performance and V̇O_2_max were observed in men but not in women, while the associations with gait speed were no longer significant. In addition, the independent associations of SB with chair rise were observed only in men, while the associations with isokinetic strength were no longer significant. Lastly, the interactive associations of MVPA and SB with handgrip strength were observed in women but not in men, and further significant MVPA × SB interactions were detected for chair rise in women and for V̇O_2_max in men. More specifically, in men, the beneficial relationship between MVPA and V̇O_2_max was attenuated with higher levels of SB. In women, the age‐related loss in chair‐rise performance was reduced in those who combined low levels of SB with high levels of MVPA, compared with those with high levels of SB and/or low levels of MVPA. The sex‐stratified analyses are presented in *Tables* *S3b*–*S3e* and illustrated in *Figures* *S1*–*S4*. Sex‐stratified estimated marginal mean values and their differences across the lifespan according to the levels of MVPA and SB are reported in *Tables* *S4b*, *S4c*, *S5b*, *S5c*, *S6b*, *S6c*, *S7b* and *S7c*.

## Discussion

The present cross‐sectional study describes the relationship between age, MVPA and SB with several indicators of physical function and performance across the adult lifespan in 499 individuals aged 20–92 years. Our main findings were that higher levels of MVPA were associated with an improved age‐related profile in gait speed, lower‐limb muscle power and cardiorespiratory fitness, independently of SB duration. On the contrary, higher SB duration exacerbated the detrimental association of age with lower‐limb muscle strength and muscle power, independently of MVPA levels. In addition, the negative association between age and handgrip strength was favourably modified by the combination of higher levels of MVPA with lower levels of SB. Furthermore, we found that some of these relationships were sex specific.

Our model revealed that higher MVPA levels were independently associated with attenuated age‐related differences in gait speed and lower‐limb muscle power in older adults and with higher aerobic capacity in young to moderately old subjects (<70 years). This is consistent with findings according to which active individuals displayed better chair‐rise performances in middle^24^ and older age^9^, ^12^, ^18^ but not during young adulthood,^11^ compared with less active people. This is also coherent with the submaximal nature of usual gait speed, which starts declining late in life, and implies that a potential preventive role of MVPA would be evident only when aging is advanced enough to deteriorate physical function. Similarly, we observed an increase in the effect of MVPA with age regarding chair‐rise performance (*Figure* *1* *B*), suggesting that MVPA provides stronger relative benefits when physical performance is reduced. The overlap of the regression lines observed in young individuals suggests that other factors than MVPA may explain the variability in chair‐rise performance in fit young adults. The weakening of the associations between MVPA and V̇O_2_max with increasing age is a known phenomenon^S24,S25^ that may be due to the fact that older individuals probably performed their MVPA at lower intensities than young individuals. The aging‐induced reduction in muscle and cardiovascular adaptations to chronic exercise^38^ may be another reason. Thus, a longitudinal approach with objective and close monitoring of PA levels is needed to confirm the hypothesis.

The inability of the accelerometers to quantify the load of resistance PAs may also explain the lack of association of MVPA with lower‐limb muscle strength and lower‐limb muscle power in young individuals. These associations may be easier to detect in older adults, whose physical performance is weak enough to be favourably impacted by other types of MVPA.^39^ Nonetheless, the literature remained conflicting because in adults aged 65 and over, no association between PA level and leg‐press performance^6^ was reported, while investigations performed in a large sample size indicated better isometric knee extension strength in active compared with inactive subjects aged 40–75.^24^

Regarding SB, our results suggest that SB may accelerate the age‐related loss in lower‐limb strength and power, regardless of MVPA levels. Further exploration remains necessary given the lack of studies in young populations^8^ and the discrepancies reported in older adults, including both significant^9^, ^10^, ^29^ and non‐significant findings.^18^, ^24^ Furthermore, as mentioned above, accelerometers cannot accurately quantify some activities, and thigh‐worn devices may inappropriately classify some resistance exercises such as leg extensions or squats as SB or sit‐to‐stand transitions instead of MVPA. This limitation may explain why, on average, highly sedentary individuals do not steadily have lower muscle strength/power values than their age‐matched low‐sedentary peers across adulthood. This is illustrated by the intersection of the regression lines in *Figure* *2* and by the unexpected positive association between SB duration and lower‐limb muscle power observed in young subjects (*Table* *S2*). The lack of associations with the other outcomes is in accordance with published work on gait speed^17^, ^18^, ^29^ and handgrip strength,^17^, ^18^, ^24^, ^28^ although the data on V̇O_2_max remain contrasted.^5^, ^8^

Our work is one of the few that considers the influence of the interaction between MVPA and SB on the associations between age and several physical outcomes throughout adulthood. Our results demonstrate a synergistic effect of MVPA and SB on age, such that the age‐associated reduction in handgrip strength was attenuated in highly active older adults who had low SB compared with less active and/or more sedentary persons (*Figure* *3* and *Table* *S2*). Notably, this age‐moderating effect was observed at the age of 64, which suggests that late adulthood might be a critical life period when MVPA and SB seem to further impact physical function. This age specificity may explain the disparate results reported from previous studies that did not specifically examine the age range of significance regarding the relationship of handgrip strength with PA,^17^, ^20^, ^22^, ^24^, ^25^, ^26^ SB^17^, ^18^, ^20^, ^24^, ^25^, ^28^ and their interactions.^33^ The lack of interaction effect on the other outcomes is in accordance with some^33^ but not all^10^ published accelerometry‐based data on gait speed and questionnaire‐based data on muscle strength^23^ in middle‐aged and older adults. Investigations on cardiorespiratory fitness are lacking, but in young individuals, it was shown that V̇O_2_max was higher in individuals combining low SB and high MVPA compared with those with high SB and low MVPA levels.^8^ This study,^8^ however, lacked subjects with low SB and low MVPA and subjects with high SB and high MVPA, which emphasizes the need for further investigation.

The mechanisms underlying the present findings involve the well‐known respective beneficial and antagonistic effects of MVPA and SB on the neuromuscular, cardiovascular and endocrine systems and on the energy metabolism. Compared with inactive and sedentary subjects, active and non‐sedentary individuals may have bigger muscle fibres, better motor‐unit recruitment, higher stroke volume and muscle oxygen consumption and a better hormone profile that all together contribute to the prevention of the age‐related loss of physical capacity.^S26,S27^

Our sex‐stratified analyses revealed that some independent associations with MVPA (chair‐rise performance and V̇O_2_max) and SB (chair rise) were observed in men but not in women. Further, we observed that the interaction of MVPA and SB was also sex dependent, with significant associations detected in women for handgrip strength and chair‐rise tests and in men for V̇O_2_max. These sex‐specific patterns suggest that the relationships examined herein might be driven by different mechanisms in men compared with women, which highlights the need for sex‐specific large‐sample research designs in the fields of PA and SB.

One limitation of the present study is its cross‐sectional design, which precludes any causal inference regarding the direction of the relationships investigated in this work. Of note, exercise interventions conducted in type 2 diabetes patients reported a reduction in sedentary time following 1 year of high‐intensity interval training.^S28^ This suggests that improvements in aerobic capacity following exercise training may lead to changes in SB. Thus, SB might be a cause but also a consequence of decreased physical capacity, which highlights the need for longitudinal studies to clarify the causality between PA, SB and physical capacity. Such longitudinal designs would also be more appropriate than cross‐sectional observations to determine the true rate of physical decline over time. The small sample size for some outcomes, including V̇O_2_max and lower‐limb muscle strength, is another weakness that reduced the chances of detecting significant associations. Also, the use of accelerometers limited the accurate quantification of the intensity of some types of MVPA, especially resistance activities. The lack of adjustment for other confounding factors, such as diet or lifestyle habits, is another limitation that may underlie unexpected findings, such as the slight increases in gait speed observed from the age of 20 to around 50 or the positive associations between SB and chair‐rise performance observed in young individuals. Also, it is not excluded that the study participants changed their PA/SB habits in response to wearing accelerometers, which may have affected the accuracy of our analyses. The strengths of our study are the objective measures of MVPA and SB, along with the exploration of their independent and interactive effects on physical function and performance over the whole spectrum of adulthood, from 20 years to more than 90. The presence of maximal performance‐based physical tests, such as V̇O_2_max and lower‐limb muscle strength, which are rare in populations over 80 years old, is an important positive point to highlight.

In conclusion, this study suggests that increasing MVPA and limiting SB may help reduce the age‐related loss in physical performance and function observed across the adult lifespan. Our findings support the current recommendations of doing at least 150 min/week of MVPA and reducing SB to prevent aging‐related deterioration in physical function and performance and delay late‐life dependency.^1^ Particular attention should be given to adults aged 60 and above, as this is the approximate age from which MVPA was significantly associated with an attenuated age‐related loss in lower‐limb power and function and a synergistic effect with SB reduction. In contrast, for maintaining aerobic fitness, we recommend engaging in aerobic activities early in adult life, as no association was found after the age of 70. Further investigations are needed to clarify the role of the type of MVPA, such as resistance exercise, that is often underestimated through accelerometry. Longitudinal study designs with long‐term follow‐ups involving a large‐age‐range population would enable us to establish the annual rate of change in physical capacity according to the levels of MVPA and SB. Lastly, sex‐specific research is also required to clarify how PA and SB may differentially modify the rate of physical decline in men compared with women, and lifestyle interventions would help clarify the extent to which modifying MVPA and SB habits could prevent the overtime decline in physical capacity.

## Conflict of interest statement

The authors declare that they have no conflict of interest.

## Supporting information


**Table S1a.** Characteristics of the participants according to each decade.
**Table S1b.** Characteristics of the participants according to each decade in men.
**Table S1c.** Characteristics of the participants according to each decade in women.
**Table S2.** Contrast analyses on the independent and interactive associations of age, MVPA and SB with physical function and performance across the adult lifespan.
**Table S3a.** Sensitivity analyses on the independent and interactive associations of age, MVPA and SB with VO_2_max and lower limb isokinetic strength across the adult lifespan after removing individuals with cardiovascular disease.
**Table S3b.** Independent and interactive associations of age, MVPA and SB with physical function and performance across the adult lifespan in men.
**Table S3c.** Independent and interactive associations of age, MVPA and SB with physical function and performance across the adult lifespan in women.
**Table S3d.** Contrast analyses on the independent and interactive associations of age, MVPA and SB with physical function and performance across the adult lifespan men.
**Table S3e.** Contrast analyses on the independent and interactive associations of age, MVPA and SB with physical function and performance across the adult lifespan in women.
**Table S4a.** Estimated marginal mean values of physical function and performance across the adult lifespan.
**Table S4b.** Estimated marginal mean values of physical function and performance across the adult lifespan in men.
**Table S4c.** Estimated marginal mean values of physical function and performance across the adult lifespan in women.
**Table S5a.** Estimated marginal mean values of physical function and performance across the adult lifespan according to the levels of MVPA.
**Table S5b.** Estimated marginal mean values of physical function and performance across the adult lifespan according to the levels of MVPA in men.
**Table S5c.** Estimated marginal mean values of physical function and performance across the adult lifespan according to the levels of MVPA in women.
**Table S6a**. Estimated marginal mean values of physical function and performance across the adult lifespan according to the levels of SB.
**Table S6b**. Estimated marginal mean values of physical function and performance across the adult lifespan according to the levels of SB in men.
**Table S6c**. Estimated marginal mean values of physical function and performance across the adult lifespan according to the levels of SB in women.
**Table S7a.** Estimated marginal mean values of physical function and performance across the adult lifespan according to the levels of MVPA and SB.
**Table S7b.** Estimated marginal mean values of physical function and performance across the adult lifespan according to the levels of MVPA and SB in men.
**Table S7c.** Estimated marginal mean values of physical function and performance across the adult lifespan according to the levels of MVPA and SB in women.
**Figure S1.** Associations between age and physical function and performance according to the levels of MVPA and SB in men.
**Figure S2.** Associations between age and VO_2_max according to the levels of MVPA and SB in men.
**Figure S3.** Associations between age and 5‐time chair rise performance according to the levels of MVPA and SB in women.
**Figure S4.** Associations between age and handgrip strength according to the levels of MVPA and SB in women.

## Appendix A.

The members of the INSPIRE platform group are as follows:

Inspire‐T human cohort group: coordinators: Sophie Guyonnet, Bruno Vellas; project managers: Lauréane Brigitte, Agathe Milhet; clinical research assistants: Elodie Paez, Emeline Muller, Sabine Le Floch; investigators: Catherine Takeda, Catherine Faisant, Françoise Lala, Gabor Abellan Van Kan, Zara Steinmeyer, Antoine Piau, Tony Macaron, Davide Angioni, Pierre‐Jean Ousset; nurses: Mélanie Comté, Nathalie Daniaud, Fanny Boissou‐Parachaud; methodology, statistical analysis and data management subgroup: Sandrine Andrieu, Christelle Cantet; body composition, V̇O_2_max, isocinetism subgroup: Yves Rolland, Philipe de Souto Barreto, Fabien Pillard; technician DXA: Bernard Teysseyre; MRI subgroup: Marie Faruch, Pierre Payoux; ICOPE subgroup: Catherine Takeda, Neda Tavassoli; biological sample collection subgroup: Marie Dorard, Bénédicte Razat, Camille Champigny, Sophie Guyonnet.

Inspire animal cohort groups: Cédric Dray, Jean‐Philippe Pradère (Fish colony); Angelo Parini, Yohan Santin (Murine cohort).

Associated research teams: Dominique Langin, Pierre Gourdy, Laurent Martinez, Anne Bouloumié, Angelo Parini (I2MC lab); Nicolas Fazilleau, Roland Liblau, Jean‐Charles Guéry, Michel Simon, Nicolas Gaudenzio, Luciana Bostan, Hicham El Costa, Nabila Jabrane Ferrat (Infinity lab); Philippe Valet, Cédric Dray, Isabelle Ader, Valérie Planat (Restore); Pierre Payoux, Patrice Peran (Tonic lab); Cyrille Delpierre, Sandrine Andrieu (CERPOP lab); Claire Rampon, Noélie Davezac, Bruno Guiard (CRCA/CBI lab); Nathalie Vergnolles, Jean‐Paul Motta, Sara Djelabi, Pauline Floch (IRSD lab); Jean‐Emmanuel Sarry (CRCT lab).
