# Supplementary material for: Cross‐sectional interactive associations of physical activity and sedentary behaviour with physical capacity across adulthood
Source: J Cachexia Sarcopenia Muscle. 2024 Apr 18;15(3):1134–45. doi: 10.1002/jcsm.13457 (PMC11154759; doi:10.1002/jcsm.13457)
Supplement: Supplementary file 1 — Table S1a. Characteristics of the participants according to each decade. Table S1b. Characteristics of the participants according to each decade in men. Table S1c. Characteristics of the participants according to each decade in women. Table S2. Contrast analyses on the independent and interactive associations of age, MVPA and SB with physical function and performance across the adult lifespan. Table S3a. Sensitivity analyses on the independent and interactive associations of age, MVPA and SB with VO2max and lower limb isokinetic strength across the adult lifespan after removing individuals with cardiovascular disease. Table S3b. Independent and interactive associations of age, MVPA and SB with physical function and performance across the adult lifespan in men. Table S3c. Independent and interactive associations of age, MVPA and SB with physical function and performance across the adult lifespan in women. Table S3d. Contrast analyses on the independent and interactive associations of age, MVPA and SB with physical function and performance across the adult lifespan men. Table S3e. Contrast analyses on the independent and interactive associations of age, MVPA and SB with physical function and performance across the adult lifespan in women. Table S4a. Estimated marginal mean values of physical function and performance across the adult lifespan. Table S4b. Estimated marginal mean values of physical function and performance across the adult lifespan in men. Table S4c. Estimated marginal mean values of physical function and performance across the adult lifespan in women. Table S5a. Estimated marginal mean values of physical function and performance across the adult lifespan according to the levels of MVPA. Table S5b. Estimated marginal mean values of physical function and performance across the adult lifespan according to the levels of MVPA in men. Table S5c. Estimated marginal mean values of physical function and performance across the adult lifespan according to the leve [file JCSM-15-1134-s001.docx]

**Cross-sectional interactive associations of physical activity and sedentary behaviour with physical capacity across adulthood**

Jérémy Raffin*, PhD^1,2^; Yves Rolland, MD, PhD^1,2,3^; Mylène Aubertin-Leheudre, PhD^4^; Jaqueline Aragoni da Silva, PhD^1,2^; Sophie Guyonnet, PhD^1,2,3^; Fabien Pillard, MD, PhD^5,6^; Bruno Vellas, MD, PhD^1,2,3^; Philipe de Souto Barreto, PhD^1,2,3^ ; for the INSPIRE group#

#Members are listed in the manuscript

**Supplementary Material**

**Content:**

**Supplementary Method description**

**Supplementary references**

**Supplementary Table 1a.** Characteristics of the participants according to each decade

**Supplementary Table 1b.** Characteristics of the participants according to each decade in men

**Supplementary Table 1c.** Characteristics of the participants according to each decade in women

**Supplementary Table 2.** Contrast analyses on the independent and interactive associations of age, MVPA and SB with physical function and performance across the adult lifespan

**Supplementary Table 3a.** Sensitivity analyses on the independent and interactive associations of age, MVPA and SB with VO2max and lower limb isokinetic strength across the adult lifespan after removing individuals with cardiovascular disease.

**Supplementary Table 3b.** Independent and interactive associations of age, MVPA and SB with physical function and performance across the adult lifespan in men

**Supplementary Table 3c.** Independent and interactive associations of age, MVPA and SB with physical function and performance across the adult lifespan in women

**Supplementary Table 3d.** Contrast analyses on the independent and interactive associations of age, MVPA and SB with physical function and performance across the adult lifespan men

**Supplementary Table 3e.** Contrast analyses on the independent and interactive associations of age, MVPA and SB with physical function and performance across the adult lifespan in women

**Supplementary Table 4a.** Estimated marginal mean values of physical function and performance across the adult lifespan

**Supplementary Table 4b.** Estimated marginal mean values of physical function and performance across the adult lifespan in men

**Supplementary Table 4c.** Estimated marginal mean values of physical function and performance across the adult lifespan in women

**Supplementary Table 5a.** Estimated marginal mean values of physical function and performance across the adult lifespan according to the levels of MVPA

**Supplementary Table 5b.** Estimated marginal mean values of physical function and performance across the adult lifespan according to the levels of MVPA in men

**Supplementary Table 5c.** Estimated marginal mean values of physical function and performance across the adult lifespan according to the levels of MVPA in women

**Supplementary Table 6a**. Estimated marginal mean values of physical function and performance across the adult lifespan according to the levels of SB

**Supplementary Table 6b**. Estimated marginal mean values of physical function and performance across the adult lifespan according to the levels of SB in men

**Supplementary Table 6c**. Estimated marginal mean values of physical function and performance across the adult lifespan according to the levels of SB in women

**Supplementary Table 7a.** Estimated marginal mean values of physical function and performance across the adult lifespan according to the levels of MVPA and SB

**Supplementary Table 7b.** Estimated marginal mean values of physical function and performance across the adult lifespan according to the levels of MVPA and SB in men

**Supplementary Table 7c.** Estimated marginal mean values of physical function and performance across the adult lifespan according to the levels of MVPA and SB in women

**Supplementary Figure 1.** Associations between age and physical function and performance according to the levels of MVPA and SB in men.

**Supplementary Figure 2.** Associations between age and VO_2_max according to the levels of MVPA and SB in men.

**Supplementary Figure 3.** Associations between age and 5-time chair rise performance according to the levels of MVPA and SB in women.

**Supplementary Figure 4.** Associations between age and handgrip strength according to the levels of MVPA and SB in women.

**Supplementary Method description**

**Assessment of physical function and performance.**

**Gait speed** was assessed using the validated 4-meter gait speed test^S3^. Participants were asked to walk two times over a 4-meter course at their usual pace and the faster of the two trials was retained to calculate gait speed in meter per second (m/s)^S3^.

**Handgrip strength** was measured in a standing position using a hydraulic hand-held dynamometer (Jamar©, Chicaco, IL, USA) adjusted to the participants’ hand size. The subjects were instructed to squeeze as hard as they could while keeping their elbow at 90° flexion. The procedure was performed three times interspaced by 2-min rest intervals using the dominant hand and the highest measure, in kilograms (kg), was kept for the analyses^S4^.

**The 5-time chair rise test** ***(estimated lower limb muscle power)*** was performed on a straight-backed chair without armrests. The participants were instructed to stand up and sit down five times as quick as possible while keeping their arms folded across their chest. The elapsed time from the starting sitting position to the end of the fifth stand was recorded with a stopwatch and retained for the analyses^S3^.

**Cardiorespiratory fitness (*V̇O_2_max*)** was assessed through a progressive exercise testing protocol performed on an ergocycle under medical supervision. The participant started cycling for 3 minutes with an initial workload set at 20% of the theoretical maximum aerobic power (tMAP) derived from the Wasserman’s equation^S5^. Afterwards, the workload was increased by 10% of the theoretical power reserve ((tMAP-0,3tMAP)*0,10) every minute until exhaustion. Cardiac activity and blood pressure was continuously monitored throughout the test using an electrocardiogram and a sphygmomanometer. The validated testing procedure was stopped when the following criteria were met: 1) maximal heart rate measured at exhaustion was superior to 90% of the aged-predicted maximal heart rate, 2) respiratory exchange ratio (RER) superior to 1.1, and 3) insufficient cycling rate.

**Lower limb isokinetic muscle strength (N⋅m)** was measured using the validated Biodex dynamometer (Biodex system 3 ©, Shirtley, NY, USA)^S6^. The maximal lower limb isokinetic strength of knee extensors from the dominant lower limb was measured using an angular velocity of 60°.s-1. After a 5-min warm-up at 50-60 rpm on a cycle ergometer using a braking resistance corresponding to 2% of body mass^S7^, the participant seated on the Biodex© dynamometer with a 85° hip flexion from the anatomical position. The lever arm was aligned with the lateral epicondyle of the knee while the trunk, the dominant thigh and leg (slightly above the medial malleolus) were stabilized with belts. The range of motion was defined at 85° (knee flexion 5° to 90°) as follows: the participant was asked to perform a voluntary maximal knee extension which defined the 0° position. Afterwards, the initial 5 degrees of flexion were completed and the dynamometer blocked. The reduction of the initial angles of flexion was done to allow the subject to exert at least 10% of the assigned torque limit. Individual calibration was performed at a position of 30° of knee flexion before each test in order to correct for gravity^S6^. During the test, the participant was instructed to keep the arms crossed upon the chest with the hands on the opposite shoulders^S8^. A specific 3-repetition trial was performed before each test^S9^ in order to reduce the “familiarization” effect. Then, after a 60-second rest interval^S10^ concentric muscular contractions were tested considering 5 repetitions for knee flexion and knee extension at 60°.s-1 (1.05 rad_s-1). The test was conducted with a real-time visual feedback given by the screen of the dynamometer^S8^. Data were collected using a sampling rate of 100Hz and were analyzed with the software *Acqknowledge*, version 4.1 (Biopac Systems, Inc., Goleta, CA, USA). Each individual curve was inspected in order to consider true isokinetic torques within 95% confidence interval of the angular velocity of 60°.s-1.

**Supplementary References**

S1. de Souto Barreto P, Guyonnet S, Ader I, Andrieu S, Casteilla L, Davezac N *et al.* The INSPIRE Research Initiative: A Program for GeroScience and Healthy Aging Research Going from Animal Models to Humans and the Healthcare System. *J Frailty Aging* 2021;**10**:86–93.

S2. Guyonnet S, Rolland Y, Takeda C, Ousset P-J, Ader I, Davezac N *et al.* The INSPIRE Bio-resource Research Platform for Healthy Aging and Geroscience: Focus on the Human Translational Research Cohort (The INSPIRE-T Cohort). *J Frailty Aging* 2020;1–11.

S3. Guralnik JM, Simonsick EM, Ferrucci L, Glynn RJ, Berkman LF, Blazer DG *et al.* A short physical performance battery assessing lower extremity function: association with self-reported disability and prediction of mortality and nursing home admission. *J Gerontol* 1994;**49**:M85-94.

S4. Roberts HC, Denison HJ, Martin HJ, Patel HP, Syddall H, Cooper C *et al.* A review of the measurement of grip strength in clinical and epidemiological studies: towards a standardised approach. *Age Ageing* 2011;**40**:423–429.

S5. Wasserman K. Principles of exercise testing and interpretation : including pathophysiology and clinical applications. *(No Title)*https://cir.nii.ac.jp/crid/1130282270541976448. Accessed 20 September 2023.

S6. Drouin JM, Valovich-mcLeod TC, Shultz SJ, Gansneder BM, Perrin DH. Reliability and validity of the Biodex system 3 pro isokinetic dynamometer velocity, torque and position measurements. *Eur J Appl Physiol* 2004;**91**:22–29.

S7. Snyder-Mackler L. Isokinetics in Human Performance. *Medicine & Science in Sports & Exercise* 2000;**32**:2153.

S8. Baltzopoulos V, Williams JG, Brodie DA. Sources of error in isokinetic dynamometry: effects of visual feedback on maximum torque. *J Orthop Sports Phys Ther* 1991;**13**:138–142.

S9. De Ste Croix MBA, Deighan MA, Ratel S, Armstrong N. Age- and sex-associated differences in isokinetic knee muscle endurance between young children and adults. *Appl Physiol Nutr Metab* 2009;**34**:725–731.

S10. Perrin DH. *Isokinetic exercise and assessment*. Human Kinetics Publishers: Champaign, IL; 1993http://books.google.com/books?id=mgNtAAAAMAAJ. Accessed 28 July 2023.

S11. Suetta C, Haddock B, Alcazar J, Noerst T, Hansen OM, Ludvig H *et al.* The Copenhagen Sarcopenia Study: lean mass, strength, power, and physical function in a Danish cohort aged 20-93 years. *J Cachexia Sarcopenia Muscle* 2019;**10**:1316–1329.

S12. Bohannon RW, Wang Y-C. Four-meter Gait Speed: Normative Values and Reliability Determined for Adults Participating in the NIH Toolbox Study. *Arch Phys Med Rehabil* 2019;**100**:509–513.

S13. Bohannon RW, Williams Andrews A. Normal walking speed: a descriptive meta-analysis. *Physiotherapy* 2011;**97**:182–189.

S14. Mayhew AJ, So HY, Ma J, Beauchamp MK, Griffith LE, Kuspinar A *et al.* Normative values for grip strength, gait speed, timed up and go, single leg balance, and chair rise derived from the Canadian longitudinal study on ageing. *Age and Ageing* 2023;**52**:afad054.

S15. Spruit MA, Sillen MJH, Groenen MTJ, Wouters EFM, Franssen FME. New Normative Values for Handgrip Strength: Results From the UK Biobank. *Journal of the American Medical Directors Association* 2013;**14**:775.e5-775.e11.

S16. Amaral CA, Amaral TLM, Monteiro GTR, Vasconcellos MTL, Portela MC. Hand grip strength: Reference values for adults and elderly people of Rio Branco, Acre, Brazil. *PLoS ONE* 2019;**14**:e0211452.

S17. Bohannon RW, Bubela DJ, Magasi SR, Wang Y-C, Gershon RC. Sit-to-stand test: Performance and determinants across the age-span. *Isokinetics and Exercise Science* 2010;**18**:235–240.

S18. Schindler IFSR, Pontes SS, Bertoni MBM, Junior GF, Júnior BRN, de Jesus FLA *et al.* A Systematic Review of Isokinetic Muscle Strength in a Healthy Population With Special Reference to Age and Gender. *Sports Health* 2023;**15**:328–332.

S19. van der Steeg GE, Takken T. Reference values for maximum oxygen uptake relative to body mass in Dutch/Flemish subjects aged 6–65 years: the LowLands Fitness Registry. *Eur J Appl Physiol* 2021;**121**:1189–1196.

S20. Shvartz E, Reibold RC. Aerobic fitness norms for males and females aged 6 to 75 years: a review. *Aviat Space Environ Med* 1990;**61**:3–11.

S21. Alcazar J, Alegre LM, Van Roie E, Magalhães JP, Nielsen BR, González-Gross M *et al.* Relative sit-to-stand power: aging trajectories, functionally relevant cut-off points, and normative data in a large European cohort. *J Cachexia Sarcopenia Muscle* 2021;**12**:921–932.

S22. Kim M, Won CW, Kim M. Muscular grip strength normative values for a Korean population from the Korea National Health and Nutrition Examination Survey, 2014-2015. *PLoS One* 2018;**13**:e0201275.

S23. Crawley MJ. *The R book*. Second edition. Wiley: Chichester, West Sussex, UK; 2013.

S24. Fitzgerald MD, Tanaka H, Tran ZV, Seals DR. Age-related declines in maximal aerobic capacity in regularly exercising vs. sedentary women: a meta-analysis. *J Appl Physiol (1985)* 1997;**83**:160–165.

S25. Tanaka H, Desouza CA, Jones PP, Stevenson ET, Davy KP, Seals DR. Greater rate of decline in maximal aerobic capacity with age in physically active vs. sedentary healthy women. *J Appl Physiol (1985)* 1997;**83**:1947–1953.

S26. Chodzko-Zajko WJ, Proctor DN, Fiatarone Singh MA, Minson CT, Nigg CR, Salem GJ *et al.* Exercise and Physical Activity for Older Adults. *Medicine & Science in Sports & Exercise* 2009;**41**:1510.

S27. Le Roux E, De Jong NP, Blanc S, Simon C, Bessesen DH, Bergouignan A. Physiology of physical inactivity, sedentary behaviours and non‐exercise activity: insights from the space bedrest model. *J Physiol* 2021;JP281064.

S28. Júdice PB, Magalhães JP, Rosa GB, Correia IR, Ekelund U, Sardinha LB. Sedentary behavior compensation to 1-year exercise RCT in patients with type 2 diabetes. *TRANSLATIONAL SPORTS MEDICINE* 2020;**3**:154–163.

| **Supplementary Table 1a. Characteristics of the participants according to each decade (1/7)** | | | |
| --- | --- | --- | --- |
|  | **20-29** | | |
| **Variable (unit)** | **Sample size** | **Statistic** | **min-max** |
| Age (years) | 55 | 24 (22 - 27) | 20 - 29 |
| Female | 55 | 35 (63.6%) |  |
| Education | 55 | 55 (100%) |  |
| No education |  | 0 (0%) |  |
| Primary school certificate |  | 0 (0%) |  |
| Secondary education diploma |  | 1 (1.8%) |  |
| High school diploma |  | 0 (0%) |  |
| University degree |  | 54 (98.2%) |  |
| Socio-professional category | 55 | 55 (100%) |  |
| Farmers |  | 0 (0%) |  |
| Artisans, shopkeepers, CEOs |  | 2 (3.6%) |  |
| Executives and intellectual professionals |  | 18 (32.7%) |  |
| Intermediate professions |  | 10 (18.2%) |  |
| Employees |  | 1 (1.8%) |  |
| Workers |  | 0 (0%) |  |
| Unemployed |  | 0 (0%) |  |
| Others |  | 24 (43.6%) |  |
| Family Income | 53 | 53 (100%) |  |
| < 1000 € - 1500 € |  | 15 (28.3%) |  |
| 1500 € - 2800 € |  | 13 (24.5%) |  |
| 2800 € - 4200+ € |  | 15 (28.3%) |  |
| Cannot or do not wish to answer |  | 10 (18.9%) |  |
| Height (m) | 55 | 1.7 (1.6 - 1.7) | 1.5 - 1.8 |
| Body weight (kg) | 55 | 65 (59.5 - 75) | 39 - 95 |
| Body mass index (kg/m²) | 55 | 22.6 (20.8 - 24.8) | 16.4 - 34.5 |
| Waist-to-hip ratio | 55 | 0.8 (0.7 - 0.8) | 0.7 - 1.3 |
| Heart rate (bpm) | 55 | 71 (63 - 81) | 38 - 100 |
| Systolic blood pressure (mmHg) | 55 | 119 (108 - 125) | 96 - 139 |
| Diastolic blood pressure (mmHg) | 55 | 70 (62 - 75) | 50 - 88 |
| Number of drugs (n) | 55 | 1 (1 - 1) | 1 - 3 |
| Cardiovascular disease | 55 | 1 (1.8%) |  |
| Chronic pulmonary disease | 55 | 1 (1.8%) |  |
| Cancer | 55 | 0 (0%) |  |
| Diabetes | 55 | 0 (0%) |  |
| Hypertension | 55 | 1 (1.8%) |  |
| Hypercholesterolemia | 55 | 1 (1.8%) |  |
| Depression | 55 | 2 (3.6%) |  |
| Osteoarthritis | 55 | 0 (0%) |  |
| Walking aid |  | 0 (0%) |  |
| MVPA (min/d) | 55 | 22.1 (14 - 30.9) | 1.6 - 65.5 |
| SB (min/d) | 55 | 412.7 (328.6 - 483.4) | 138 - 676.2 |
| Gait speed (m/s) | 55 | 1.3 (1.2 - 1.4) | 0.8 - 1.8 |
| Handgrip strength (kg) | 55 | 36 (30.5 - 46) | 22 - 68 |
| 5-time sit-to-stand (n) | 55 | 7.2 (5.5 - 8.4) | 4.7 - 11.9 |
| V̇O_2_max (ml/kg/min) | 6 | 41.5 (40 - 53) | 20 - 57 |
| Isokinetic Strength (kg) | 6 | 120 (104 - 164) | 100 - 170 |
| Mini mental state examination score | 55 | 30 (29 - 30) | 25 - 30 |
| Short physical performance battery score | 55 | 12 (12 - 12) | 11 - 12 |

Continuous variables are presented as median (Q1-Q3) along with minimum and maximum values. Categorical variables are presented as n (%).

| **Supplementary Table 1a. Characteristics of the participants according to each decade (2/7)** | | | |
| --- | --- | --- | --- |
|  | **30-39** | | |
| **Variable (unit)** | **Sample size** | **Statistic** | **min-max** |
| Age (years) | 51 | 33 (31 - 37) | 30 - 39 |
| Female | 51 | 36 (70.6%) |  |
| Education | 51 | 51 (100%) |  |
| No education |  | 0 (0%) |  |
| Primary school certificate |  | 0 (0%) |  |
| Secondary education diploma |  | 0 (0%) |  |
| High school diploma |  | 1 (2%) |  |
| University degree |  | 50 (98%) |  |
| Socio-professional category | 51 | 51 (100%) |  |
| Farmers |  | 0 (0%) |  |
| Artisans, shopkeepers, CEOs |  | 3 (5.9%) |  |
| Executives and intellectual professionals |  | 15 (29.4%) |  |
| Intermediate professions |  | 26 (51%) |  |
| Employees |  | 2 (3.9%) |  |
| Workers |  | 0 (0%) |  |
| Unemployed |  | 1 (2%) |  |
| Others |  | 4 (7.8%) |  |
| Family Income | 51 | 51 (100%) |  |
| < 1000 € - 1500 € |  | 3 (5.9%) |  |
| 1500 € - 2800 € |  | 13 (25.5%) |  |
| 2800 € - 4200+ € |  | 35 (68.6%) |  |
| Cannot or do not wish to answer |  | 0 (0%) |  |
| Height (m) | 51 | 1.7 (1.6 - 1.7) | 1.5 - 1.9 |
| Body weight (kg) | 51 | 66 (58 - 80) | 39.5 - 142 |
| Body mass index (kg/m²) | 51 | 23.3 (21.3 - 27.5) | 15.6 - 40.4 |
| Waist-to-hip ratio | 51 | 0.8 (0.8 - 0.9) | 0.7 - 1 |
| Heart rate (bpm) | 51 | 66 (58 - 73) | 45 - 97 |
| Systolic blood pressure (mmHg) | 51 | 117 (106 - 122) | 87 - 154 |
| Diastolic blood pressure (mmHg) | 51 | 68 (60 - 76) | 50 - 93 |
| Number of drugs (n) | 51 | 1 (1 - 1) | 1 - 4 |
| Cardiovascular disease | 51 | 0 (0%) |  |
| Chronic pulmonary disease | 51 | 3 (5.9%) |  |
| Cancer | 51 | 0 (0%) |  |
| Diabetes | 51 | 0 (0%) |  |
| Hypertension | 51 | 0 (0%) |  |
| Hypercholesterolemia | 51 | 1 (2%) |  |
| Depression | 51 | 9 (17.6%) |  |
| Osteoarthritis | 51 | 0 (0%) |  |
| Walking aid | 51 | 0 (0%) |  |
| MVPA (min/d) | 51 | 26 (10.9 - 42.2) | 0.5 - 88.1 |
| SB (min/d) | 51 | 384.6 (314 - 462.4) | 237.8 - 708.7 |
| Gait speed (m/s) | 51 | 1.3 (1.2 - 1.4) | 0.9 - 1.8 |
| Handgrip strength (kg) | 51 | 36 (32 - 44) | 22 - 62 |
| 5-time sit-to-stand (n) | 51 | 6.6 (5.7 - 8.5) | 3.9 - 16 |
| V̇O_2_max (ml/kg/min) | 16 | 24.5 (22 - 34.5) | 20 - 39 |
| Isokinetic Strength (kg) | 17 | 131 (101 - 146) | 78 - 244 |
| Mini mental state examination score | 51 | 29 (29 - 30) | 26 - 30 |
| Short physical performance battery score | 51 | 12 (12 - 12) | 10 - 12 |

Continuous variables are presented as median (Q1-Q3) along with minimum and maximum values. Categorical variables are presented as n (%).

| **Supplementary Table 1a. Characteristics of the participants according to each decade (3/7)** | | | |
| --- | --- | --- | --- |
|  | **40-49** | | |
| **Variable (unit)** | **Sample size** | **Statistic** | **min-max** |
| Age (years) | 46 | 45 (44 - 48) | 40 - 49 |
| Female | 46 | 32 (69.6%) |  |
| Education | 46 | 46 (100%) |  |
| No education |  | 0 (0%) |  |
| Primary school certificate |  | 0 (0%) |  |
| Secondary education diploma |  | 0 (0%) |  |
| High school diploma |  | 2 (4.3%) |  |
| University degree |  | 44 (95.7%) |  |
| Socio-professional category | 46 | 46 (100%) |  |
| Farmers |  | 1 (2.2%) |  |
| Artisans, shopkeepers, CEOs |  | 1 (2.2%) |  |
| Executives and intellectual professionals |  | 23 (50%) |  |
| Intermediate professions |  | 17 (37%) |  |
| Employees |  | 3 (6.5%) |  |
| Workers |  | 0 (0%) |  |
| Unemployed |  | 0 (0%) |  |
| Others |  | 1 (2.2%) |  |
| Family Income | 46 | 46 (100%) |  |
| < 1000 € - 1500 € |  | 3 (6.5%) |  |
| 1500 € - 2800 € |  | 8 (17.4%) |  |
| 2800 € - 4200+ € |  | 33 (71.7%) |  |
| Cannot or do not wish to answer |  | 2 (4.3%) |  |
| Height (m) | 46 | 1.7 (1.6 - 1.7) | 1.6 - 1.9 |
| Body weight (kg) | 46 | 61 (56 - 69) | 47 - 96 |
| Body mass index (kg/m²) | 46 | 21.9 (20.9 - 25.9) | 16.3 - 32 |
| Waist-to-hip ratio | 46 | 0.8 (0.8 - 0.9) | 0.7 - 1 |
| Heart rate (bpm) | 46 | 66 (61 - 73) | 50 - 87 |
| Systolic blood pressure (mmHg) | 46 | 116.5 (107 - 125) | 90 - 155 |
| Diastolic blood pressure (mmHg) | 46 | 71.5 (62 - 79) | 53 - 92 |
| Number of drugs (n) | 46 | 1 (1 - 1) | 1 - 3 |
| Cardiovascular disease | 46 | 1 (2.2%) |  |
| Chronic pulmonary disease | 46 | 2 (4.3%) |  |
| Cancer | 46 | 2 (4.3%) |  |
| Diabetes | 46 | 0 (0%) |  |
| Hypertension | 46 | 1 (2.2%) |  |
| Hypercholesterolemia | 46 | 1 (2.2%) |  |
| Depression | 46 | 4 (8.7%) |  |
| Osteoarthritis | 46 | 0 (0%) |  |
| Walking aid | 46 | 0 (0%) |  |
| MVPA (min/d) | 46 | 29.9 (14.1 - 48.5) | 5.9 - 93.4 |
| SB (min/d) | 46 | 399.8 (348.3 - 458.1) | 200.6 - 657.6 |
| Gait speed (m/s) | 46 | 1.3 (1.2 - 1.5) | 0.8 - 1.6 |
| Handgrip strength (kg) | 46 | 34 (30 - 44) | 13.5 - 63 |
| 5-time sit-to-stand (n) | 46 | 7.9 (6.4 - 9.2) | 4 - 16 |
| V̇O_2_max (ml/kg/min) | 12 | 28.5 (25.5 - 36) | 18 - 48 |
| Isokinetic Strength (kg) | 13 | 120 (85 - 132) | 42 - 198 |
| Mini mental state examination score | 46 | 29.5 (29 - 30) | 26 - 30 |
| Short physical performance battery score | 46 | 12 (12 - 12) | 10 - 12 |

Continuous variables are presented as median (Q1-Q3) along with minimum and maximum values. Categorical variables are presented as n (%).

| **Supplementary Table 1a. Characteristics of the participants according to each decade (4/7)** | | | |
| --- | --- | --- | --- |
|  | **50-59** | | |
| **Variable (unit)** | **Sample size** | **Statistic** | **min-max** |
| Age (years) | 67 | 54 (52 - 57) | 50 - 59 |
| Female | 67 | 53 (79.1%) |  |
| Education | 67 | 67 (100%) |  |
| No education |  | 0 (0%) |  |
| Primary school certificate |  | 0 (0%) |  |
| Secondary education diploma |  | 5 (7.5%) |  |
| High school diploma |  | 8 (11.9%) |  |
| University degree |  | 54 (80.6%) |  |
| Socio-professional category | 67 | 67 (100%) |  |
| Farmers |  | 1 (1.5%) |  |
| Artisans, shopkeepers, CEOs |  | 2 (3%) |  |
| Executives and intellectual professionals |  | 23 (34.3%) |  |
| Intermediate professions |  | 23 (34.3%) |  |
| Employees |  | 14 (20.9%) |  |
| Workers |  | 0 (0%) |  |
| Unemployed |  | 0 (0%) |  |
| Others |  | 4 (6%) |  |
| Family Income | 67 | 67 (100%) |  |
| < 1000 € - 1500 € |  | 6 (9%) |  |
| 1500 € - 2800 € |  | 13 (19.4%) |  |
| 2800 € - 4200+ € |  | 47 (70.1%) |  |
| Cannot or do not wish to answer |  | 1 (1.5%) |  |
| Height (m) | 67 | 1.7 (1.6 - 1.7) | 1.5 - 1.9 |
| Body weight (kg) | 67 | 67 (58 - 78) | 50.5 - 102 |
| Body mass index (kg/m²) | 67 | 24.1 (21.7 - 26.8) | 18.6 - 36.8 |
| Waist-to-hip ratio | 67 | 0.8 (0.8 - 0.9) | 0.7 - 1 |
| Heart rate (bpm) | 67 | 65 (59 - 72) | 47 - 86 |
| Systolic blood pressure (mmHg) | 67 | 119 (110 - 128) | 84 - 168 |
| Diastolic blood pressure (mmHg) | 67 | 73 (67 - 78) | 50 - 103 |
| Number of drugs (n) | 67 | 1 (1 - 2) | 1 - 6 |
| Cardiovascular disease | 67 | 2 (3%) |  |
| Chronic pulmonary disease | 67 | 6 (9%) |  |
| Cancer | 67 | 6 (9%) |  |
| Diabetes | 67 | 1 (1.5%) |  |
| Hypertension | 67 | 8 (11.9%) |  |
| Hypercholesterolemia | 67 | 5 (7.5%) |  |
| Depression | 67 | 12 (17.9%) |  |
| Osteoarthritis | 67 | 1 (1.5%) |  |
| Walking aid | 67 | 0 (0%) |  |
| MVPA (min/d) | 67 | 21.9 (8.7 - 39.9) | 3.4 - 74.8 |
| SB (min/d) | 67 | 436.4 (362.3 - 501.7) | 110.4 - 735.5 |
| Gait speed (m/s) | 67 | 1.4 (1.2 - 1.5) | 0.9 - 1.7 |
| Handgrip strength (kg) | 67 | 32 (28 - 38) | 19.5 - 66 |
| 5-time sit-to-stand (n) | 67 | 8.2 (7 - 9.1) | 4.7 - 13 |
| V̇O_2_max (ml/kg/min) | 22 | 30 (25 - 33) | 21 - 48 |
| Isokinetic Strength (kg) | 22 | 109.5 (86 - 115) | 61 - 222 |
| Mini mental state examination score | 67 | 30 (29 - 30) | 23 - 30 |
| Short physical performance battery score | 66 | 12 (12 - 12) | 11 - 12 |

Continuous variables are presented as median (Q1-Q3) along with minimum and maximum values. Categorical variables are presented as n (%).

| **Supplementary Table 1a. Characteristics of the participants according to each decade (5/7)** | | | |
| --- | --- | --- | --- |
|  | **60-69** | | |
| **Variable (unit)** | **Sample size** | **Statistic** | **min-max** |
| Age (years) | 95 | 64 (62 - 67) | 60 - 69 |
| Female | 95 | 61 (64.2%) |  |
| Education | 95 | 95 (100%) |  |
| No education |  | 0 (0%) |  |
| Primary school certificate |  | 2 (2.1%) |  |
| Secondary education diploma |  | 8 (8.4%) |  |
| High school diploma |  | 21 (22.1%) |  |
| University degree |  | 64 (67.4%) |  |
| Socio-professional category | 95 | 95 (100%) |  |
| Farmers |  | 1 (1.1%) |  |
| Artisans, shopkeepers, CEOs |  | 6 (6.3%) |  |
| Executives and intellectual professionals |  | 37 (38.9%) |  |
| Intermediate professions |  | 29 (30.5%) |  |
| Employees |  | 17 (17.9%) |  |
| Workers |  | 0 (0%) |  |
| Unemployed |  | 3 (3.2%) |  |
| Others |  | 2 (2.1%) |  |
| Family Income | 95 | 95 (100%) |  |
| < 1000 € - 1500 € |  | 5 (5.3%) |  |
| 1500 € - 2800 € |  | 26 (27.4%) |  |
| 2800 € - 4200+ € |  | 63 (66.3%) |  |
| Cannot or do not wish to answer |  | 1 (1.1%) |  |
| Height (m) | 95 | 1.7 (1.6 - 1.7) | 1.5 - 2 |
| Body weight (kg) | 95 | 67 (57.2 - 79) | 42.5 - 111 |
| Body mass index (kg/m²) | 95 | 24.7 (21.7 - 27.6) | 17.9 - 37.5 |
| Waist-to-hip ratio | 95 | 0.9 (0.8 - 0.9) | 0.6 - 1.1 |
| Heart rate (bpm) | 94 | 66 (60 - 72) | 43 - 98 |
| Systolic blood pressure (mmHg) | 94 | 129 (116 - 143) | 98 - 180 |
| Diastolic blood pressure (mmHg) | 94 | 74 (63 - 82) | 53 - 112 |
| Number of drugs (n) | 95 | 1 (1 - 3) | 1 - 15 |
| Cardiovascular disease | 95 | 15 (15.8%) |  |
| Chronic pulmonary disease | 95 | 4 (4.2%) |  |
| Cancer | 95 | 2 (2.1%) |  |
| Diabetes | 95 | 2 (2.1%) |  |
| Hypertension | 95 | 18 (18.9%) |  |
| Hypercholesterolemia | 95 | 17 (17.9%) |  |
| Depression | 95 | 12 (12.6%) |  |
| Osteoarthritis | 95 | 9 (9.5%) |  |
| Walking aid | 93 | 0 (0%) |  |
| MVPA (min/d) | 95 | 25.5 (12.2 - 41.8) | 0.4 - 126.1 |
| SB (min/d) | 95 | 354.2 (301.9 - 438.1) | 165.1 - 637.3 |
| Gait speed (m/s) | 95 | 1.3 (1.2 - 1.4) | 0.9 - 1.9 |
| Handgrip strength (kg) | 95 | 32 (26 - 40) | 8 - 63 |
| 5-time sit-to-stand (n) | 95 | 9 (7.7 - 10) | 5.2 - 17 |
| V̇O_2_max (ml/kg/min) | 45 | 25 (21 - 28) | 17 - 42 |
| Isokinetic Strength (kg) | 47 | 94 (72 - 114) | 47 - 199 |
| Mini mental state examination score | 95 | 29 (28 - 30) | 24 - 30 |
| Short physical performance battery score | 94 | 12 (12 - 12) | 9 - 12 |

Continuous variables are presented as median (Q1-Q3) along with minimum and maximum values. Categorical variables are presented as n (%).

| **Supplementary Table 1a. Characteristics of the participants according to each decade (6/7)** | | | |
| --- | --- | --- | --- |
|  | **70-80** | | |
| **Variable (unit)** | **Sample size** | **Statistic** | **min-max** |
| Age (years) | 108 | 73 (71 - 77) | 70 - 79 |
| Female | 108 | 60 (55.6%) |  |
| Education | 108 | 108 (100%) |  |
| No education |  | 0 (0%) |  |
| Primary school certificate |  | 5 (4.6%) |  |
| Secondary education diploma |  | 9 (8.3%) |  |
| High school diploma |  | 29 (26.9%) |  |
| University degree |  | 65 (60.2%) |  |
| Socio-professional category | 108 | 108 (100%) |  |
| Farmers |  | 1 (0.9%) |  |
| Artisans, shopkeepers, CEOs |  | 5 (4.6%) |  |
| Executives and intellectual professionals |  | 41 (38%) |  |
| Intermediate professions |  | 28 (25.9%) |  |
| Employees |  | 21 (19.4%) |  |
| Workers |  | 4 (3.7%) |  |
| Unemployed |  | 1 (0.9%) |  |
| Others |  | 7 (6.5%) |  |
| Family Income | 107 | 107 (100%) |  |
| < 1000 € - 1500 € |  | 8 (7.5%) |  |
| 1500 € - 2800 € |  | 28 (26.2%) |  |
| 2800 € - 4200+ € |  | 67 (62.6%) |  |
| Cannot or do not wish to answer |  | 4 (3.7%) |  |
| Height (m) | 108 | 1.7 (1.6 - 1.7) | 1.4 - 1.9 |
| Body weight (kg) | 107 | 67 (58 - 77) | 45 - 109 |
| Body mass index (kg/m²) | 107 | 24.8 (22.7 - 28.3) | 18.8 - 36.6 |
| Waist-to-hip ratio | 108 | 0.9 (0.8 - 1) | 0.7 - 1.2 |
| Heart rate (bpm) | 108 | 67 (62 - 73.5) | 44 - 95 |
| Systolic blood pressure (mmHg) | 108 | 140 (126.5 - 153.5) | 108 - 193 |
| Diastolic blood pressure (mmHg) | 108 | 74 (67 - 79) | 54 - 102 |
| Number of drugs (n) | 108 | 2 (1 - 4) | 1 - 14 |
| Cardiovascular disease | 108 | 22 (20.4%) |  |
| Chronic pulmonary disease | 108 | 11 (10.2%) |  |
| Cancer | 108 | 16 (14.8%) |  |
| Diabetes | 108 | 5 (4.6%) |  |
| Hypertension | 108 | 43 (39.8%) |  |
| Hypercholesterolemia | 108 | 32 (29.6%) |  |
| Depression | 108 | 21 (19.4%) |  |
| Osteoarthritis | 108 | 14 (13%) |  |
| Walking aid | 103 | 3 (2.9%) |  |
| MVPA (min/d) | 108 | 18.8 (10.1 - 31.8) | 0.9 - 89 |
| SB (min/d) | 108 | 359.9 (303.3 - 418.8) | 120.8 - 692.5 |
| Gait speed (m/s) | 108 | 1.2 (1.1 - 1.3) | 0.7 - 2.1 |
| Handgrip strength (kg) | 108 | 28 (23 - 37.8) | 12 - 56 |
| 5-time sit-to-stand (n) | 108 | 9.5 (8 - 10.8) | 5 - 16 |
| V̇O_2_max (ml/kg/min) | 52 | 20 (17.5 - 23) | 13 - 34 |
| Isokinetic Strength (kg) | 51 | 90 (67 - 117) | 45 - 191 |
| Mini mental state examination score | 107 | 29 (28 - 30) | 20 - 30 |
| Short physical performance battery score | 106 | 12 (12 - 12) | 9 - 12 |

Continuous variables are presented as median (Q1-Q3) along with minimum and maximum values. Categorical variables are presented as n (%).

| **Supplementary Table 1a. Characteristics of the participants according to each decade (7/7)** | | | |
| --- | --- | --- | --- |
|  | **80+** | | |
| **Variable (unit)** | **Sample size** | **Statistic** | **min-max** |
| Age (years) | 77 | 83 (81 - 86) | 80 - 92 |
| Female | 77 | 38 (49.4%) |  |
| Education | 77 | 77 (100%) |  |
| No education |  | 1 (1.3%) |  |
| Primary school certificate |  | 12 (15.6%) |  |
| Secondary education diploma |  | 13 (16.9%) |  |
| High school diploma |  | 9 (11.7%) |  |
| University degree |  | 42 (54.5%) |  |
| Socio-professional category | 77 | 77 (100%) |  |
| Farmers |  | 1 (1.3%) |  |
| Artisans, shopkeepers, CEOs |  | 6 (7.8%) |  |
| Executives and intellectual professionals |  | 19 (24.7%) |  |
| Intermediate professions |  | 31 (40.3%) |  |
| Employees |  | 18 (23.4%) |  |
| Workers |  | 0 (0%) |  |
| Unemployed |  | 1 (1.3%) |  |
| Others |  | 1 (1.3%) |  |
| Family Income | 77 | 77 (100%) |  |
| < 1000 € - 1500 € |  | 5 (6.5%) |  |
| 1500 € - 2800 € |  | 35 (45.5%) |  |
| 2800 € - 4200+ € |  | 32 (41.6%) |  |
| Cannot or do not wish to answer |  | 5 (6.5%) |  |
| Height (m) | 77 | 1.6 (1.6 - 1.7) | 1.3 - 1.8 |
| Body weight (kg) | 77 | 65 (57.5 - 72) | 39 - 104 |
| Body mass index (kg/m²) | 77 | 24.6 (22.3 - 26.9) | 17.3 - 36.2 |
| Waist-to-hip ratio | 77 | 0.9 (0.9 - 1) | 0.7 - 1.1 |
| Heart rate (bpm) | 77 | 68 (59 - 76) | 48 - 100 |
| Systolic blood pressure (mmHg) | 77 | 145 (132 - 158) | 103 - 217 |
| Diastolic blood pressure (mmHg) | 77 | 72 (65 - 81) | 56 - 100 |
| Number of drugs (n) | 77 | 4 (2 - 7) | 1 - 13 |
| Cardiovascular disease | 77 | 31 (40.3%) |  |
| Chronic pulmonary disease | 77 | 7 (9.1%) |  |
| Cancer | 77 | 17 (22.1%) |  |
| Diabetes | 77 | 6 (7.8%) |  |
| Hypertension | 77 | 53 (68.8%) |  |
| Hypercholesterolemia | 77 | 26 (33.8%) |  |
| Depression | 77 | 12 (15.6%) |  |
| Osteoarthritis | 77 | 16 (20.8%) |  |
| Walking aid | 76 | 9 (11.8%) |  |
| MVPA (min/d) | 77 | 15.3 (4.4 - 28.1) | 0 - 127.9 |
| SB (min/d) | 77 | 416.3 (336.3 - 511) | 120.8 - 809.6 |
| Gait speed (m/s) | 77 | 1.1 (0.9 - 1.3) | 0.4 - 1.9 |
| Handgrip strength (kg) | 77 | 26 (20 - 32) | 6 - 50 |
| 5-time sit-to-stand (n) | 74 | 9.7 (8.4 - 11.4) | 4.5 - 16.1 |
| V̇O_2_max (ml/kg/min) | 12 | 20.5 (17 - 22) | 14 - 25 |
| Isokinetic Strength (kg) | 12 | 57.5 (35 - 71) | 29 - 110 |
| Mini mental state examination score | 77 | 29 (27 - 30) | 16 - 30 |
| Short physical performance battery score | 76 | 12 (11 - 12) | 2 - 12 |

Continuous variables are presented as median (Q1-Q3) along with minimum and maximum values. Categorical variables are presented as n (%).

| **Supplementary Table 1b. Characteristics of the participants according to each decade in men (1/8)** | | | |
| --- | --- | --- | --- |
|  | **Total** | | |
| **Variable (unit)** | **Sample size** | **Statistic** | **min-max** |
| Age (years) | 184 | 67 (47.5 - 78) | 20 - 90 |
| Education | 184 | 184 (100%) |  |
| No education |  | 1 (0.5%) |  |
| Primary school certificate |  | 10 (5.4%) |  |
| Secondary education diploma |  | 11 (6%) |  |
| High school diploma |  | 24 (13%) |  |
| University degree |  | 138 (75%) |  |
| Socio-professional category | 184 | 184 (100%) |  |
| Farmers |  | 2 (1.1%) |  |
| Artisans, shopkeepers, CEOs |  | 15 (8.2%) |  |
| Executives and intellectual professionals |  | 85 (46.2%) |  |
| Intermediate professions |  | 52 (28.3%) |  |
| Employees |  | 11 (6%) |  |
| Workers |  | 1 (0.5%) |  |
| Unemployed |  | 0 (0%) |  |
| Others |  | 18 (9.8%) |  |
| Family Income | 182 | 182 (100%) |  |
| < 1000 € - 1500 € |  | 10 (5.5%) |  |
| 1500 € - 2800 € |  | 42 (23.1%) |  |
| 2800 € - 4200+ € |  | 123 (67.6%) |  |
| Cannot or do not wish to answer |  | 7 (3.8%) |  |
| Height (m) | 184 | 1.7 (1.7 - 1.8) | 1.5 - 2 |
| Body weight (kg) | 184 | 77 (68.9 - 86.7) | 54.5 - 142 |
| Body mass index (kg/m²) | 184 | 25.6 (23.2 - 28.4) | 18.3 - 39.8 |
| Waist-to-hip ratio | 184 | 0.9 (0.9 - 1) | 0.7 - 1.2 |
| Heart rate (bpm) | 183 | 63 (58 - 72) | 38 - 100 |
| Systolic blood pressure (mmHg) | 183 | 135 (122 - 153) | 99 - 217 |
| Diastolic blood pressure (mmHg) | 183 | 75 (68 - 82) | 56 - 100 |
| Number of drugs (n) | 184 | 1 (1 - 3) | 1 - 13 |
| Cardiovascular disease | 184 | 34 (18.5%) |  |
| Chronic pulmonary disease | 184 | 19 (10.3%) |  |
| Cancer | 184 | 17 (9.2%) |  |
| Diabetes | 184 | 10 (5.4%) |  |
| Hypertension | 184 | 65 (35.3%) |  |
| Hypercholesterolemia | 184 | 44 (23.9%) |  |
| Depression | 184 | 20 (10.9%) |  |
| Osteoarthritis | 184 | 18 (9.8%) |  |
| Walking aid | 179 | 7 (3.9%) |  |
| MVPA (min/d) | 184 | 21.3 (11 - 38.8) | 0.2 - 127.9 |
| SB (min/d) | 184 | 418.1 (334 - 504.2) | 120.8 - 809.6 |
| Gait speed (m/s) | 184 | 1.3 (1.1 - 1.4) | 0.5 - 2.1 |
| Handgrip strength (kg) | 184 | 42 (34 - 49.5) | 19 - 68 |
| 5-time sit-to-stand (n) | 182 | 9 (7.2 - 10) | 3.9 - 16.1 |
| V̇O_2_max (ml/kg/min) | 69 | 27 (22 - 34) | 13 - 57 |
| Isokinetic Strength (kg) | 73 | 119 (97 - 142) | 52 - 244 |
| Mini mental state examination score | 184 | 29 (28 - 30) | 23 - 30 |
| Short physical performance battery score | 183 | 12 (12 - 12) | 4 - 12 |

Continuous variables are presented as median (Q1-Q3) along with minimum and maximum values. Categorical variables are presented as n (%).

| **Supplementary Table 1b. Characteristics of the participants according to each decade in men (2/8)** | | | |
| --- | --- | --- | --- |
|  | **20-29** | | |
| **Variable (unit)** | **Sample size** | **Statistic** | **min-max** |
| Age (years) | 20 | 24.5 (22.5 - 27) | 20 - 28 |
| Education | 20 | 20 (100%) |  |
| No education |  | 0 (0%) |  |
| Primary school certificate |  | 0 (0%) |  |
| Secondary education diploma |  | 1 (5%) |  |
| High school diploma |  | 0 (0%) |  |
| University degree |  | 19 (95%) |  |
| Socio-professional category | 20 | 20 (100%) |  |
| Farmers |  | 0 (0%) |  |
| Artisans, shopkeepers, CEOs |  | 1 (5%) |  |
| Executives and intellectual professionals |  | 7 (35%) |  |
| Intermediate professions |  | 2 (10%) |  |
| Employees |  | 1 (5%) |  |
| Workers |  | 0 (0%) |  |
| Unemployed |  | 0 (0%) |  |
| Others |  | 9 (45%) |  |
| Family Income | 19 | 19 (100%) |  |
| < 1000 € - 1500 € |  | 4 (21.1%) |  |
| 1500 € - 2800 € |  | 5 (26.3%) |  |
| 2800 € - 4200+ € |  | 8 (42.1%) |  |
| Cannot or do not wish to answer |  | 2 (10.5%) |  |
| Height (m) | 20 | 1.7 (1.7 - 1.8) | 1.6 - 1.8 |
| Body weight (kg) | 20 | 73.8 (66.8 - 81) | 60 - 95 |
| Body mass index (kg/m²) | 20 | 23.5 (21.7 - 25.7) | 20.4 - 34.5 |
| Waist-to-hip ratio | 20 | 0.8 (0.8 - 0.9) | 0.7 - 1 |
| Heart rate (bpm) | 20 | 65.5 (57 - 74.5) | 38 - 98 |
| Systolic blood pressure (mmHg) | 20 | 124.5 (116.5 - 131.5) | 102 - 139 |
| Diastolic blood pressure (mmHg) | 20 | 71 (62 - 75) | 56 - 81 |
| Number of drugs (n) | 20 | 1 (1 - 1) | 1 - 1 |
| Cardiovascular disease | 20 | 1 (5%) |  |
| Chronic pulmonary disease | 20 | 1 (5%) |  |
| Cancer | 20 | 0 (0%) |  |
| Diabetes | 20 | 0 (0%) |  |
| Hypertension | 20 | 0 (0%) |  |
| Hypercholesterolemia | 20 | 0 (0%) |  |
| Depression | 20 | 1 (5%) |  |
| Osteoarthritis | 20 | 0 (0%) |  |
| Walking aid | 20 | 0 (0%) |  |
| MVPA (min/d) | 20 | 23 (14.6 - 33.9) | 1.6 - 57.2 |
| SB (min/d) | 20 | 438.9 (344.9 - 555.8) | 138 - 635.5 |
| Gait speed (m/s) | 20 | 1.4 (1.3 - 1.4) | 1 - 1.5 |
| Handgrip strength (kg) | 20 | 47 (42 - 55) | 33 - 68 |
| 5-time sit-to-stand (n) | 20 | 7.1 (5.3 - 8.5) | 4.7 - 11 |
| V̇O_2_max (ml/kg/min) | 4 | 47 (40.5 - 55) | 40 - 57 |
| Isokinetic Strength (kg) | 4 | 148 (118 - 167) | 104 - 170 |
| Mini mental state examination score | 20 | 30 (29.5 - 30) | 28 - 30 |
| Short physical performance battery score | 20 | 12 (12 - 12) | 12 - 12 |

Continuous variables are presented as median (Q1-Q3) along with minimum and maximum values. Categorical variables are presented as n (%).

| **Supplementary Table 1b. Characteristics of the participants according to each decade in men (3/8)** | | | |
| --- | --- | --- | --- |
|  | **30-39** | | |
| **Variable (unit)** | **Sample size** | **Statistic** | **min-max** |
| Age (years) | 15 | 36 (31 - 39) | 30 - 39 |
| Education | 15 | 15 (100%) |  |
| No education |  | 0 (0%) |  |
| Primary school certificate |  | 0 (0%) |  |
| Secondary education diploma |  | 0 (0%) |  |
| High school diploma |  | 0 (0%) |  |
| University degree |  | 15 (100%) |  |
| Socio-professional category | 15 | 15 (100%) |  |
| Farmers |  | 0 (0%) |  |
| Artisans, shopkeepers, CEOs |  | 1 (6.7%) |  |
| Executives and intellectual professionals |  | 4 (26.7%) |  |
| Intermediate professions |  | 8 (53.3%) |  |
| Employees |  | 0 (0%) |  |
| Workers |  | 0 (0%) |  |
| Unemployed |  | 0 (0%) |  |
| Others |  | 2 (13.3%) |  |
| Family Income | 15 | 15 (100%) |  |
| < 1000 € - 1500 € |  | 1 (6.7%) |  |
| 1500 € - 2800 € |  | 4 (26.7%) |  |
| 2800 € - 4200+ € |  | 10 (66.7%) |  |
| Cannot or do not wish to answer |  | 0 (0%) |  |
| Height (m) | 15 | 1.8 (1.7 - 1.8) | 1.7 - 1.9 |
| Body weight (kg) | 15 | 81 (68.8 - 86) | 58 - 142 |
| Body mass index (kg/m²) | 15 | 24.3 (22.3 - 28.3) | 20.6 - 39.8 |
| Waist-to-hip ratio | 15 | 0.9 (0.8 - 0.9) | 0.8 - 1 |
| Heart rate (bpm) | 15 | 63 (57 - 81) | 52 - 97 |
| Systolic blood pressure (mmHg) | 15 | 117 (115 - 126) | 99 - 147 |
| Diastolic blood pressure (mmHg) | 15 | 71 (61 - 83) | 56 - 93 |
| Number of drugs (n) | 15 | 1 (1 - 1) | 1 - 3 |
| Cardiovascular disease | 15 | 0 (0%) |  |
| Chronic pulmonary disease | 15 | 0 (0%) |  |
| Cancer | 15 | 0 (0%) |  |
| Diabetes | 15 | 0 (0%) |  |
| Hypertension | 15 | 0 (0%) |  |
| Hypercholesterolemia | 15 | 0 (0%) |  |
| Depression | 15 | 4 (26.7%) |  |
| Osteoarthritis | 15 | 0 (0%) |  |
| Walking aid | 15 | 0 (0%) |  |
| MVPA (min/d) | 15 | 37 (12.3 - 44.1) | 0.5 - 70.5 |
| SB (min/d) | 15 | 411.8 (314 - 478.3) | 237.8 - 616.9 |
| Gait speed (m/s) | 15 | 1.3 (1.2 - 1.4) | 1.1 - 1.7 |
| Handgrip strength (kg) | 15 | 52 (45 - 60) | 39.5 - 62 |
| 5-time sit-to-stand (n) | 15 | 6.6 (5.2 - 10) | 3.9 - 13.1 |
| V̇O_2_max (ml/kg/min) | 4 | 29.5 (23 - 35) | 21 - 36 |
| Isokinetic Strength (kg) | 5 | 172 (135 - 212) | 134 - 244 |
| Mini mental state examination score | 15 | 29 (28 - 30) | 26 - 30 |
| Short physical performance battery score | 15 | 12 (12 - 12) | 11 - 12 |

Continuous variables are presented as median (Q1-Q3) along with minimum and maximum values. Categorical variables are presented as n (%).

| **Supplementary Table 1b. Characteristics of the participants according to each decade in men (4/8)** | | | |
| --- | --- | --- | --- |
|  | **40-49** | | |
| **Variable (unit)** | **Sample size** | **Statistic** | **min-max** |
| Age (years) | 14 | 45 (42 - 47) | 40 - 49 |
| Education | 14 | 14 (100%) |  |
| No education |  | 0 (0%) |  |
| Primary school certificate |  | 0 (0%) |  |
| Secondary education diploma |  | 0 (0%) |  |
| High school diploma |  | 0 (0%) |  |
| University degree |  | 14 (100%) |  |
| Socio-professional category | 14 | 14 (100%) |  |
| Farmers |  | 0 (0%) |  |
| Artisans, shopkeepers, CEOs |  | 1 (7.1%) |  |
| Executives and intellectual professionals |  | 8 (57.1%) |  |
| Intermediate professions |  | 4 (28.6%) |  |
| Employees |  | 1 (7.1%) |  |
| Workers |  | 0 (0%) |  |
| Unemployed |  | 0 (0%) |  |
| Others |  | 0 (0%) |  |
| Family Income | 14 | 14 (100%) |  |
| < 1000 € - 1500 € |  | 1 (7.1%) |  |
| 1500 € - 2800 € |  | 1 (7.1%) |  |
| 2800 € - 4200+ € |  | 11 (78.6%) |  |
| Cannot or do not wish to answer |  | 1 (7.1%) |  |
| Height (m) | 14 | 1.8 (1.7 - 1.8) | 1.7 - 1.9 |
| Body weight (kg) | 14 | 76 (66.5 - 89) | 58 - 96 |
| Body mass index (kg/m²) | 14 | 24.6 (22 - 28.9) | 18.3 - 30 |
| Waist-to-hip ratio | 14 | 0.9 (0.9 - 0.9) | 0.8 - 1 |
| Heart rate (bpm) | 14 | 62.5 (56 - 65) | 50 - 75 |
| Systolic blood pressure (mmHg) | 14 | 123.5 (116 - 139) | 109 - 155 |
| Diastolic blood pressure (mmHg) | 14 | 78.5 (70 - 90) | 62 - 92 |
| Number of drugs (n) | 14 | 1 (1 - 1) | 1 - 3 |
| Cardiovascular disease | 14 | 0 (0%) |  |
| Chronic pulmonary disease | 14 | 2 (14.3%) |  |
| Cancer | 14 | 0 (0%) |  |
| Diabetes | 14 | 0 (0%) |  |
| Hypertension | 14 | 1 (7.1%) |  |
| Hypercholesterolemia | 14 | 1 (7.1%) |  |
| Depression | 14 | 0 (0%) |  |
| Osteoarthritis | 14 | 0 (0%) |  |
| Walking aid | 14 | 0 (0%) |  |
| MVPA (min/d) | 14 | 32.6 (17 - 53.6) | 8.3 - 93.4 |
| SB (min/d) | 14 | 420 (381.6 - 491.2) | 321 - 581.4 |
| Gait speed (m/s) | 14 | 1.3 (1.1 - 1.4) | 1 - 1.6 |
| Handgrip strength (kg) | 14 | 50 (44 - 52) | 26 - 63 |
| 5-time sit-to-stand (n) | 14 | 9.2 (7.3 - 9.7) | 5.8 - 10.4 |
| V̇O_2_max (ml/kg/min) | 5 | 29 (28 - 44) | 27 - 48 |
| Isokinetic Strength (kg) | 6 | 127.5 (120 - 135) | 85 - 198 |
| Mini mental state examination score | 14 | 29.5 (29 - 30) | 28 - 30 |
| Short physical performance battery score | 14 | 12 (12 - 12) | 12 - 12 |

Continuous variables are presented as median (Q1-Q3) along with minimum and maximum values. Categorical variables are presented as n (%).

| **Supplementary Table 1b. Characteristics of the participants according to each decade in men (5/8)** | | | |
| --- | --- | --- | --- |
|  | **50-59** | | |
| **Variable (unit)** | **Sample size** | **Statistic** | **min-max** |
| Age (years) | 14 | 52.5 (51 - 56) | 50 - 58 |
| Education | 14 | 14 (100%) |  |
| No education |  | 0 (0%) |  |
| Primary school certificate |  | 0 (0%) |  |
| Secondary education diploma |  | 0 (0%) |  |
| High school diploma |  | 2 (14.3%) |  |
| University degree |  | 12 (85.7%) |  |
| Socio-professional category | 14 | 14 (100%) |  |
| Farmers |  | 0 (0%) |  |
| Artisans, shopkeepers, CEOs |  | 2 (14.3%) |  |
| Executives and intellectual professionals |  | 4 (28.6%) |  |
| Intermediate professions |  | 3 (21.4%) |  |
| Employees |  | 2 (14.3%) |  |
| Workers |  | 0 (0%) |  |
| Unemployed |  | 0 (0%) |  |
| Others |  | 3 (21.4%) |  |
| Family Income | 14 | 14 (100%) |  |
| < 1000 € - 1500 € |  | 1 (7.1%) |  |
| 1500 € - 2800 € |  | 1 (7.1%) |  |
| 2800 € - 4200+ € |  | 11 (78.6%) |  |
| Cannot or do not wish to answer |  | 1 (7.1%) |  |
| Height (m) | 14 | 1.8 (1.8 - 1.8) | 1.7 - 1.9 |
| Body weight (kg) | 14 | 86.5 (77 - 91) | 71 - 97 |
| Body mass index (kg/m²) | 14 | 25.9 (24.1 - 27.8) | 23.5 - 29.3 |
| Waist-to-hip ratio | 14 | 0.9 (0.9 - 0.9) | 0.8 - 1 |
| Heart rate (bpm) | 14 | 63.5 (56 - 71) | 51 - 74 |
| Systolic blood pressure (mmHg) | 14 | 121.5 (114 - 133) | 104 - 168 |
| Diastolic blood pressure (mmHg) | 14 | 78.5 (72 - 85) | 58 - 97 |
| Number of drugs (n) | 14 | 1 (1 - 1) | 1 - 3 |
| Cardiovascular disease | 14 | 0 (0%) |  |
| Chronic pulmonary disease | 14 | 2 (14.3%) |  |
| Cancer | 14 | 1 (7.1%) |  |
| Diabetes | 14 | 1 (7.1%) |  |
| Hypertension | 14 | 1 (7.1%) |  |
| Hypercholesterolemia | 14 | 2 (14.3%) |  |
| Depression | 14 | 2 (14.3%) |  |
| Osteoarthritis | 14 | 0 (0%) |  |
| Walking aid | 14 | 0 (0%) |  |
| MVPA (min/d) | 14 | 13.7 (11.2 - 46.9) | 4.6 - 74.8 |
| SB (min/d) | 14 | 462.2 (415.5 - 508.4) | 360.3 - 590.5 |
| Gait speed (m/s) | 14 | 1.3 (1.2 - 1.4) | 1.1 - 1.6 |
| Handgrip strength (kg) | 14 | 51 (44 - 62) | 37 - 66 |
| 5-time sit-to-stand (n) | 14 | 8.2 (7 - 9) | 7 - 12 |
| V̇O_2_max (ml/kg/min) | 8 | 34 (31.5 - 38.5) | 28 - 48 |
| Isokinetic Strength (kg) | 8 | 114.5 (111.5 - 181.5) | 109 - 222 |
| Mini mental state examination score | 14 | 30 (29 - 30) | 27 - 30 |
| Short physical performance battery score | 14 | 12 (12 - 12) | 11 - 12 |

Continuous variables are presented as median (Q1-Q3) along with minimum and maximum values. Categorical variables are presented as n (%).

| **Supplementary Table 1b. Characteristics of the participants according to each decade in men (6/8)** | | | |
| --- | --- | --- | --- |
|  | **60-69** | | |
| **Variable (unit)** | **Sample size** | **Statistic** | **min-max** |
| Age (years) | 34 | 64.5 (62 - 67) | 60 - 68 |
| Education | 34 | 34 (100%) |  |
| No education |  | 0 (0%) |  |
| Primary school certificate |  | 2 (5.9%) |  |
| Secondary education diploma |  | 3 (8.8%) |  |
| High school diploma |  | 9 (26.5%) |  |
| University degree |  | 20 (58.8%) |  |
| Socio-professional category | 34 | 34 (100%) |  |
| Farmers |  | 1 (2.9%) |  |
| Artisans, shopkeepers, CEOs |  | 4 (11.8%) |  |
| Executives and intellectual professionals |  | 20 (58.8%) |  |
| Intermediate professions |  | 7 (20.6%) |  |
| Employees |  | 2 (5.9%) |  |
| Workers |  | 0 (0%) |  |
| Unemployed |  | 0 (0%) |  |
| Others |  | 0 (0%) |  |
| Family Income | 34 | 34 (100%) |  |
| < 1000 € - 1500 € |  | 0 (0%) |  |
| 1500 € - 2800 € |  | 8 (23.5%) |  |
| 2800 € - 4200+ € |  | 26 (76.5%) |  |
| Cannot or do not wish to answer |  | 0 (0%) |  |
| Height (m) | 34 | 1.7 (1.7 - 1.8) | 1.7 - 2 |
| Body weight (kg) | 34 | 80.5 (73.5 - 86.8) | 60 - 111 |
| Body mass index (kg/m²) | 34 | 25.8 (23.6 - 29.1) | 19.8 - 37.5 |
| Waist-to-hip ratio | 34 | 1 (0.9 - 1) | 0.9 - 1.1 |
| Heart rate (bpm) | 33 | 63 (57 - 70) | 43 - 82 |
| Systolic blood pressure (mmHg) | 33 | 138 (125 - 155) | 106 - 180 |
| Diastolic blood pressure (mmHg) | 33 | 80 (74 - 85) | 58 - 98 |
| Number of drugs (n) | 34 | 1 (1 - 3) | 1 - 7 |
| Cardiovascular disease | 34 | 6 (17.6%) |  |
| Chronic pulmonary disease | 34 | 3 (8.8%) |  |
| Cancer | 34 | 1 (2.9%) |  |
| Diabetes | 34 | 2 (5.9%) |  |
| Hypertension | 34 | 11 (32.4%) |  |
| Hypercholesterolemia | 34 | 7 (20.6%) |  |
| Depression | 34 | 2 (5.9%) |  |
| Osteoarthritis | 34 | 3 (8.8%) |  |
| Walking aid | 34 | 0 (0%) |  |
| MVPA (min/d) | 34 | 25.5 (11.4 - 39.7) | 3.5 - 94.5 |
| SB (min/d) | 34 | 364 (306.2 - 453.1) | 165.1 - 637.3 |
| Gait speed (m/s) | 34 | 1.3 (1.2 - 1.4) | 1 - 1.9 |
| Handgrip strength (kg) | 34 | 44.5 (40 - 49) | 20 - 63 |
| 5-time sit-to-stand (n) | 34 | 9 (8.2 - 10) | 5.2 - 13.1 |
| V̇O_2_max (ml/kg/min) | 18 | 30 (25 - 35) | 20 - 42 |
| Isokinetic Strength (kg) | 20 | 122.5 (96 - 141.5) | 66 - 199 |
| Mini mental state examination score | 34 | 29 (28 - 30) | 26 - 30 |
| Short physical performance battery score | 34 | 12 (12 - 12) | 11 - 12 |

Continuous variables are presented as median (Q1-Q3) along with minimum and maximum values. Categorical variables are presented as n (%).

| **Supplementary Table 1b. Characteristics of the participants according to each decade in men (7/8)** | | | |
| --- | --- | --- | --- |
|  | **70-80** | | |
| **Variable (unit)** | **Sample size** | **Statistic** | **min-max** |
| Age (years) | 48 | 73 (72 - 77) | 70 - 79 |
| Education | 48 | 48 (100%) |  |
| No education |  | 0 (0%) |  |
| Primary school certificate |  | 2 (4.2%) |  |
| Secondary education diploma |  | 2 (4.2%) |  |
| High school diploma |  | 9 (18.8%) |  |
| University degree |  | 35 (72.9%) |  |
| Socio-professional category | 48 | 48 (100%) |  |
| Farmers |  | 1 (2.1%) |  |
| Artisans, shopkeepers, CEOs |  | 2 (4.2%) |  |
| Executives and intellectual professionals |  | 28 (58.3%) |  |
| Intermediate professions |  | 11 (22.9%) |  |
| Employees |  | 1 (2.1%) |  |
| Workers |  | 1 (2.1%) |  |
| Unemployed |  | 0 (0%) |  |
| Others |  | 4 (8.3%) |  |
| Family Income | 47 | 47 (100%) |  |
| < 1000 € - 1500 € |  | 2 (4.3%) |  |
| 1500 € - 2800 € |  | 8 (17%) |  |
| 2800 € - 4200+ € |  | 35 (74.5%) |  |
| Cannot or do not wish to answer |  | 2 (4.3%) |  |
| Height (m) | 48 | 1.7 (1.7 - 1.8) | 1.5 - 1.9 |
| Body weight (kg) | 48 | 76.5 (69.3 - 88.5) | 57.5 - 109 |
| Body mass index (kg/m²) | 48 | 26.2 (24.1 - 29.4) | 19.4 - 36 |
| Waist-to-hip ratio | 48 | 1 (0.9 - 1) | 0.8 - 1.2 |
| Heart rate (bpm) | 48 | 65 (60 - 74) | 44 - 95 |
| Systolic blood pressure (mmHg) | 48 | 141.5 (132 - 153.5) | 110 - 193 |
| Diastolic blood pressure (mmHg) | 48 | 75 (70 - 81) | 56 - 98 |
| Number of drugs (n) | 48 | 2 (1 - 4) | 1 - 10 |
| Cardiovascular disease | 48 | 9 (18.8%) |  |
| Chronic pulmonary disease | 48 | 7 (14.6%) |  |
| Cancer | 48 | 8 (16.7%) |  |
| Diabetes | 48 | 2 (4.2%) |  |
| Hypertension | 48 | 21 (43.8%) |  |
| Hypercholesterolemia | 48 | 17 (35.4%) |  |
| Depression | 48 | 5 (10.4%) |  |
| Osteoarthritis | 48 | 7 (14.6%) |  |
| Walking aid | 44 | 3 (6.8%) |  |
| MVPA (min/d) | 48 | 18.6 (12.2 - 36.1) | 1.5 - 89 |
| SB (min/d) | 48 | 382.6 (311.8 - 461.2) | 157.1 - 567.5 |
| Gait speed (m/s) | 48 | 1.2 (1.1 - 1.4) | 0.9 - 2.1 |
| Handgrip strength (kg) | 48 | 39 (33.5 - 46) | 20 - 56 |
| 5-time sit-to-stand (n) | 48 | 9.3 (8 - 10.8) | 5 - 14.8 |
| V̇O_2_max (ml/kg/min) | 24 | 22 (19 - 27.5) | 13 - 34 |
| Isokinetic Strength (kg) | 24 | 115 (96.5 - 135.5) | 65 - 191 |
| Mini mental state examination score | 48 | 29 (28 - 29.5) | 27 - 30 |
| Short physical performance battery score | 48 | 12 (12 - 12) | 9 - 12 |

Continuous variables are presented as median (Q1-Q3) along with minimum and maximum values. Categorical variables are presented as n (%).

| **Supplementary Table 1b. Characteristics of the participants according to each decade in men (8/8)** | | | |
| --- | --- | --- | --- |
|  | **80+** | | |
| **Variable (unit)** | **Sample size** | **Statistic** | **min-max** |
| Age (years) | 39 | 83 (81 - 86) | 80 - 90 |
| Education | 39 | 39 (100%) |  |
| No education |  | 1 (2.6%) |  |
| Primary school certificate |  | 6 (15.4%) |  |
| Secondary education diploma |  | 5 (12.8%) |  |
| High school diploma |  | 4 (10.3%) |  |
| University degree |  | 23 (59%) |  |
| Socio-professional category | 39 | 39 (100%) |  |
| Farmers |  | 0 (0%) |  |
| Artisans, shopkeepers, CEOs |  | 4 (10.3%) |  |
| Executives and intellectual professionals |  | 14 (35.9%) |  |
| Intermediate professions |  | 17 (43.6%) |  |
| Employees |  | 4 (10.3%) |  |
| Workers |  | 0 (0%) |  |
| Unemployed |  | 0 (0%) |  |
| Others |  | 0 (0%) |  |
| Family Income | 39 | 39 (100%) |  |
| < 1000 € - 1500 € |  | 1 (2.6%) |  |
| 1500 € - 2800 € |  | 15 (38.5%) |  |
| 2800 € - 4200+ € |  | 22 (56.4%) |  |
| Cannot or do not wish to answer |  | 1 (2.6%) |  |
| Height (m) | 39 | 1.7 (1.7 - 1.7) | 1.5 - 1.8 |
| Body weight (kg) | 39 | 70.5 (65 - 80) | 54.5 - 104 |
| Body mass index (kg/m²) | 39 | 24.9 (23.3 - 27.1) | 21.4 - 34.8 |
| Waist-to-hip ratio | 39 | 1 (0.9 - 1.1) | 0.8 - 1.1 |
| Heart rate (bpm) | 39 | 62 (55 - 73) | 48 - 100 |
| Systolic blood pressure (mmHg) | 39 | 153 (128 - 162) | 103 - 217 |
| Diastolic blood pressure (mmHg) | 39 | 73 (67 - 81) | 57 - 100 |
| Number of drugs (n) | 39 | 4 (3 - 7) | 1 - 13 |
| Cardiovascular disease | 39 | 18 (46.2%) |  |
| Chronic pulmonary disease | 39 | 4 (10.3%) |  |
| Cancer | 39 | 7 (17.9%) |  |
| Diabetes | 39 | 5 (12.8%) |  |
| Hypertension | 39 | 31 (79.5%) |  |
| Hypercholesterolemia | 39 | 17 (43.6%) |  |
| Depression | 39 | 6 (15.4%) |  |
| Osteoarthritis | 39 | 8 (20.5%) |  |
| Walking aid | 38 | 4 (10.5%) |  |
| MVPA (min/d) | 39 | 17.9 (3.9 - 30.3) | 0.2 - 127.9 |
| SB (min/d) | 39 | 485.3 (364.6 - 547.2) | 120.8 - 809.6 |
| Gait speed (m/s) | 39 | 1.1 (0.9 - 1.2) | 0.5 - 1.9 |
| Handgrip strength (kg) | 39 | 32 (28 - 40) | 19 - 50 |
| 5-time sit-to-stand (n) | 37 | 9.7 (8.9 - 11.1) | 4.5 - 16.1 |
| V̇O_2_max (ml/kg/min) | 6 | 21 (20 - 23) | 16 - 25 |
| Isokinetic Strength (kg) | 6 | 69 (63 - 79) | 52 - 110 |
| Mini mental state examination score | 39 | 28 (27 - 30) | 23 - 30 |
| Short physical performance battery score | 38 | 12 (11 - 12) | 4 - 12 |

Continuous variables are presented as median (Q1-Q3) along with minimum and maximum values. Categorical variables are presented as n (%).

| **Supplementary Table 1c. Characteristics of the participants according to each decade in women (1/8)** | | | |
| --- | --- | --- | --- |
|  | **Total** | | |
| **Variable (unit)** | **Sample size** | **Statistic** | **min-max** |
| Age (years) | 315 | 60 (44 - 72) | 20 - 92 |
| Education | 315 | 315 (100%) |  |
| No education |  | 0 (0%) |  |
| Primary school certificate |  | 9 (2.9%) |  |
| Secondary education diploma |  | 25 (7.9%) |  |
| High school diploma |  | 46 (14.6%) |  |
| University degree |  | 235 (74.6%) |  |
| Socio-professional category | 315 | 315 (100%) |  |
| Farmers |  | 3 (1%) |  |
| Artisans, shopkeepers, CEOs |  | 10 (3.2%) |  |
| Executives and intellectual professionals |  | 91 (28.9%) |  |
| Intermediate professions |  | 112 (35.6%) |  |
| Employees |  | 65 (20.6%) |  |
| Workers |  | 3 (1%) |  |
| Unemployed |  | 6 (1.9%) |  |
| Others |  | 25 (7.9%) |  |
| Family Income | 314 | 314 (100%) |  |
| < 1000 € - 1500 € |  | 35 (11.1%) |  |
| 1500 € - 2800 € |  | 94 (29.9%) |  |
| 2800 € - 4200+ € |  | 169 (53.8%) |  |
| Cannot or do not wish to answer |  | 16 (5.1%) |  |
| Height (m) | 315 | 1.6 (1.6 - 1.7) | 1.3 - 1.8 |
| Body weight (kg) | 314 | 61 (55 - 67) | 39 - 106 |
| Body mass index (kg/m²) | 314 | 23.1 (21.2 - 26.3) | 15.6 - 40.4 |
| Waist-to-hip ratio | 315 | 0.8 (0.8 - 0.9) | 0.6 - 1.3 |
| Heart rate (bpm) | 315 | 68 (62 - 75) | 45 - 100 |
| Systolic blood pressure (mmHg) | 315 | 122 (112 - 137) | 84 - 185 |
| Diastolic blood pressure (mmHg) | 315 | 70 (62 - 77) | 50 - 112 |
| Number of drugs (n) | 315 | 1 (1 - 3) | 1 - 15 |
| Cardiovascular disease | 315 | 38 (12.1%) |  |
| Chronic pulmonary disease | 315 | 15 (4.8%) |  |
| Cancer | 315 | 26 (8.3%) |  |
| Diabetes | 315 | 4 (1.3%) |  |
| Hypertension | 315 | 59 (18.7%) |  |
| Hypercholesterolemia | 315 | 39 (12.4%) |  |
| Depression | 315 | 52 (16.5%) |  |
| Osteoarthritis | 315 | 22 (7%) |  |
| Walking aid | 312 | 5 (1.6%) |  |
| MVPA (min/d) | 315 | 21.4 (10.1 - 36.3) | 0 - 126.1 |
| SB (min/d) | 315 | 380.6 (316.9 - 448.8) | 110.4 - 735.5 |
| Gait speed (m/s) | 315 | 1.3 (1.1 - 1.4) | 0.4 - 1.9 |
| Handgrip strength (kg) | 315 | 28 (24 - 33) | 6 - 49 |
| 5-time sit-to-stand (n) | 314 | 8.4 (7 - 10) | 4 - 17 |
| V̇O_2_max (ml/kg/min) | 96 | 22 (19 - 26) | 14 - 42 |
| Isokinetic Strength (kg) | 95 | 82 (62 - 101) | 29 - 177 |
| Mini mental state examination score | 314 | 29 (28 - 30) | 16 - 30 |
| Short physical performance battery score | 311 | 12 (12 - 12) | 2 - 12 |

Continuous variables are presented as median (Q1-Q3) along with minimum and maximum values. Categorical variables are presented as n (%).

| **Supplementary Table 1c. Characteristics of the participants according to each decade in women (2/8)** | | | |
| --- | --- | --- | --- |
|  | **20-29** | | |
| **Variable (unit)** | **Sample size** | **Statistic** | **min-max** |
| Age (years) | 35 | 24 (22 - 26) | 20 - 29 |
| Education | 35 | 35 (100%) |  |
| No education |  | 0 (0%) |  |
| Primary school certificate |  | 0 (0%) |  |
| Secondary education diploma |  | 0 (0%) |  |
| High school diploma |  | 0 (0%) |  |
| University degree |  | 35 (100%) |  |
| Socio-professional category | 35 | 35 (100%) |  |
| Farmers |  | 0 (0%) |  |
| Artisans, shopkeepers, CEOs |  | 1 (2.9%) |  |
| Executives and intellectual professionals |  | 11 (31.4%) |  |
| Intermediate professions |  | 8 (22.9%) |  |
| Employees |  | 0 (0%) |  |
| Workers |  | 0 (0%) |  |
| Unemployed |  | 0 (0%) |  |
| Others |  | 15 (42.9%) |  |
| Family Income | 34 | 34 (100%) |  |
| < 1000 € - 1500 € |  | 11 (32.4%) |  |
| 1500 € - 2800 € |  | 8 (23.5%) |  |
| 2800 € - 4200+ € |  | 7 (20.6%) |  |
| Cannot or do not wish to answer |  | 8 (23.5%) |  |
| Height (m) | 35 | 1.7 (1.6 - 1.7) | 1.5 - 1.8 |
| Body weight (kg) | 35 | 61 (57 - 66.5) | 39 - 83 |
| Body mass index (kg/m²) | 35 | 22.3 (20.6 - 24.7) | 16.4 - 30.5 |
| Waist-to-hip ratio | 35 | 0.8 (0.7 - 0.8) | 0.7 - 1.3 |
| Heart rate (bpm) | 35 | 75 (66 - 83) | 47 - 100 |
| Systolic blood pressure (mmHg) | 35 | 115 (105 - 121) | 96 - 134 |
| Diastolic blood pressure (mmHg) | 35 | 68 (62 - 76) | 50 - 88 |
| Number of drugs (n) | 35 | 1 (1 - 1) | 1 - 3 |
| Cardiovascular disease | 35 | 0 (0%) |  |
| Chronic pulmonary disease | 35 | 0 (0%) |  |
| Cancer | 35 | 0 (0%) |  |
| Diabetes | 35 | 0 (0%) |  |
| Hypertension | 35 | 1 (2.9%) |  |
| Hypercholesterolemia | 35 | 1 (2.9%) |  |
| Depression | 35 | 1 (2.9%) |  |
| Osteoarthritis | 35 | 0 (0%) |  |
| Walking aid | 35 | 0 (0%) |  |
| MVPA (min/d) | 35 | 20.1 (12.5 - 30.9) | 4.2 - 65.5 |
| SB (min/d) | 35 | 409.1 (328.6 - 464.5) | 197.2 - 676.2 |
| Gait speed (m/s) | 35 | 1.3 (1.2 - 1.4) | 0.8 - 1.8 |
| Handgrip strength (kg) | 35 | 32 (28 - 36) | 22 - 49 |
| 5-time sit-to-stand (n) | 35 | 7.3 (5.5 - 8.4) | 4.8 - 11.9 |
| V̇O_2_max (ml/kg/min) | 2 | 31 (20 - 42) | 20 - 42 |
| Isokinetic Strength (kg) | 2 | 104 (100 - 108) | 100 - 108 |
| Mini mental state examination score | 35 | 30 (29 - 30) | 25 - 30 |
| Short physical performance battery score | 35 | 12 (12 - 12) | 11 - 12 |

Continuous variables are presented as median (Q1-Q3) along with minimum and maximum values. Categorical variables are presented as n (%).

| **Supplementary Table 1c. Characteristics of the participants according to each decade in women (3/8)** | | | |
| --- | --- | --- | --- |
|  | **30-39** | | |
| **Variable (unit)** | **Sample size** | **Statistic** | **min-max** |
| Age (years) | 36 | 33 (31 - 37) | 30 - 39 |
| Education | 36 | 36 (100%) |  |
| No education |  | 0 (0%) |  |
| Primary school certificate |  | 0 (0%) |  |
| Secondary education diploma |  | 0 (0%) |  |
| High school diploma |  | 1 (2.8%) |  |
| University degree |  | 35 (97.2%) |  |
| Socio-professional category | 36 | 36 (100%) |  |
| Farmers |  | 0 (0%) |  |
| Artisans, shopkeepers, CEOs |  | 2 (5.6%) |  |
| Executives and intellectual professionals |  | 11 (30.6%) |  |
| Intermediate professions |  | 18 (50%) |  |
| Employees |  | 2 (5.6%) |  |
| Workers |  | 0 (0%) |  |
| Unemployed |  | 1 (2.8%) |  |
| Others |  | 2 (5.6%) |  |
| Family Income | 36 | 36 (100%) |  |
| < 1000 € - 1500 € |  | 2 (5.6%) |  |
| 1500 € - 2800 € |  | 9 (25%) |  |
| 2800 € - 4200+ € |  | 25 (69.4%) |  |
| Cannot or do not wish to answer |  | 0 (0%) |  |
| Height (m) | 36 | 1.6 (1.6 - 1.7) | 1.5 - 1.8 |
| Body weight (kg) | 36 | 63 (54.5 - 74) | 39.5 - 106 |
| Body mass index (kg/m²) | 36 | 23 (21.3 - 26.7) | 15.6 - 40.4 |
| Waist-to-hip ratio | 36 | 0.8 (0.7 - 0.8) | 0.7 - 0.9 |
| Heart rate (bpm) | 36 | 66.5 (59 - 73) | 45 - 92 |
| Systolic blood pressure (mmHg) | 36 | 116 (105 - 121.5) | 87 - 154 |
| Diastolic blood pressure (mmHg) | 36 | 68 (59 - 74) | 50 - 93 |
| Number of drugs (n) | 36 | 1 (1 - 1) | 1 - 4 |
| Cardiovascular disease | 36 | 0 (0%) |  |
| Chronic pulmonary disease | 36 | 3 (8.3%) |  |
| Cancer | 36 | 0 (0%) |  |
| Diabetes | 36 | 0 (0%) |  |
| Hypertension | 36 | 0 (0%) |  |
| Hypercholesterolemia | 36 | 1 (2.8%) |  |
| Depression | 36 | 5 (13.9%) |  |
| Osteoarthritis | 36 | 0 (0%) |  |
| Walking aid | 36 | 0 (0%) |  |
| MVPA (min/d) | 36 | 25.8 (10.1 - 37.8) | 3.2 - 88.1 |
| SB (min/d) | 36 | 377.5 (322.1 - 454.2) | 255.4 - 708.7 |
| Gait speed (m/s) | 36 | 1.3 (1.2 - 1.5) | 0.9 - 1.8 |
| Handgrip strength (kg) | 36 | 34 (29.5 - 36) | 22 - 41 |
| 5-time sit-to-stand (n) | 36 | 6.4 (6 - 8.1) | 4.5 - 16 |
| V̇O_2_max (ml/kg/min) | 12 | 24 (22 - 33) | 20 - 39 |
| Isokinetic Strength (kg) | 12 | 112.5 (90.5 - 137) | 78 - 152 |
| Mini mental state examination score | 36 | 29 (29 - 30) | 27 - 30 |
| Short physical performance battery score | 36 | 12 (12 - 12) | 10 - 12 |

Continuous variables are presented as median (Q1-Q3) along with minimum and maximum values. Categorical variables are presented as n (%).

| **Supplementary Table 1c. Characteristics of the participants according to each decade in women (4/8)** | | | |
| --- | --- | --- | --- |
|  | **40-49** | | |
| **Variable (unit)** | **Sample size** | **Statistic** | **min-max** |
| Age (years) | 32 | 45 (44.5 - 48) | 41 - 49 |
| Education | 32 | 32 (100%) |  |
| No education |  | 0 (0%) |  |
| Primary school certificate |  | 0 (0%) |  |
| Secondary education diploma |  | 0 (0%) |  |
| High school diploma |  | 2 (6.3%) |  |
| University degree |  | 30 (93.8%) |  |
| Socio-professional category | 32 | 32 (100%) |  |
| Farmers |  | 1 (3.1%) |  |
| Artisans, shopkeepers, CEOs |  | 0 (0%) |  |
| Executives and intellectual professionals |  | 15 (46.9%) |  |
| Intermediate professions |  | 13 (40.6%) |  |
| Employees |  | 2 (6.3%) |  |
| Workers |  | 0 (0%) |  |
| Unemployed |  | 0 (0%) |  |
| Others |  | 1 (3.1%) |  |
| Family Income | 32 | 32 (100%) |  |
| < 1000 € - 1500 € |  | 2 (6.3%) |  |
| 1500 € - 2800 € |  | 7 (21.9%) |  |
| 2800 € - 4200+ € |  | 22 (68.8%) |  |
| Cannot or do not wish to answer |  | 1 (3.1%) |  |
| Height (m) | 32 | 1.6 (1.6 - 1.7) | 1.6 - 1.8 |
| Body weight (kg) | 32 | 58 (54 - 61.8) | 47 - 86 |
| Body mass index (kg/m²) | 32 | 21.7 (20.8 - 22.6) | 16.3 - 32 |
| Waist-to-hip ratio | 32 | 0.8 (0.8 - 0.8) | 0.7 - 0.9 |
| Heart rate (bpm) | 32 | 69.5 (62.5 - 73.5) | 51 - 87 |
| Systolic blood pressure (mmHg) | 32 | 112 (105.5 - 122.5) | 90 - 137 |
| Diastolic blood pressure (mmHg) | 32 | 68.5 (61 - 78.5) | 53 - 92 |
| Number of drugs (n) | 32 | 1 (1 - 1) | 1 - 3 |
| Cardiovascular disease | 32 | 1 (3.1%) |  |
| Chronic pulmonary disease | 32 | 0 (0%) |  |
| Cancer | 32 | 2 (6.3%) |  |
| Diabetes | 32 | 0 (0%) |  |
| Hypertension | 32 | 0 (0%) |  |
| Hypercholesterolemia | 32 | 0 (0%) |  |
| Depression | 32 | 4 (12.5%) |  |
| Osteoarthritis | 32 | 0 (0%) |  |
| Walking aid | 32 | 0 (0%) |  |
| MVPA (min/d) | 32 | 26.9 (14 - 44) | 5.9 - 89.2 |
| SB (min/d) | 32 | 394.3 (343.6 - 455.6) | 200.6 - 657.6 |
| Gait speed (m/s) | 32 | 1.4 (1.3 - 1.5) | 0.8 - 1.6 |
| Handgrip strength (kg) | 32 | 32 (29.5 - 34) | 13.5 - 44 |
| 5-time sit-to-stand (n) | 32 | 7.3 (6.2 - 8.3) | 4 - 16 |
| V̇O_2_max (ml/kg/min) | 7 | 28 (24 - 35) | 18 - 37 |
| Isokinetic Strength (kg) | 7 | 97 (63 - 123) | 42 - 145 |
| Mini mental state examination score | 32 | 29.5 (29 - 30) | 26 - 30 |
| Short physical performance battery score | 32 | 12 (12 - 12) | 10 - 12 |

Continuous variables are presented as median (Q1-Q3) along with minimum and maximum values. Categorical variables are presented as n (%).

| **Supplementary Table 1c. Characteristics of the participants according to each decade in women (5/8)** | | | |
| --- | --- | --- | --- |
|  | **50-59** | | |
| **Variable (unit)** | **Sample size** | **Statistic** | **min-max** |
| Age (years) | 53 | 55 (52 - 57) | 50 - 59 |
| Education | 53 | 53 (100%) |  |
| No education |  | 0 (0%) |  |
| Primary school certificate |  | 0 (0%) |  |
| Secondary education diploma |  | 5 (9.4%) |  |
| High school diploma |  | 6 (11.3%) |  |
| University degree |  | 42 (79.2%) |  |
| Socio-professional category | 53 | 53 (100%) |  |
| Farmers |  | 1 (1.9%) |  |
| Artisans, shopkeepers, CEOs |  | 0 (0%) |  |
| Executives and intellectual professionals |  | 19 (35.8%) |  |
| Intermediate professions |  | 20 (37.7%) |  |
| Employees |  | 12 (22.6%) |  |
| Workers |  | 0 (0%) |  |
| Unemployed |  | 0 (0%) |  |
| Others |  | 1 (1.9%) |  |
| Family Income | 53 | 53 (100%) |  |
| < 1000 € - 1500 € |  | 5 (9.4%) |  |
| 1500 € - 2800 € |  | 12 (22.6%) |  |
| 2800 € - 4200+ € |  | 36 (67.9%) |  |
| Cannot or do not wish to answer |  | 0 (0%) |  |
| Height (m) | 53 | 1.6 (1.6 - 1.7) | 1.5 - 1.8 |
| Body weight (kg) | 53 | 63 (57 - 69.5) | 50.5 - 102 |
| Body mass index (kg/m²) | 53 | 23.3 (21.3 - 26.8) | 18.6 - 36.8 |
| Waist-to-hip ratio | 53 | 0.8 (0.8 - 0.9) | 0.7 - 1 |
| Heart rate (bpm) | 53 | 65 (60 - 72) | 47 - 86 |
| Systolic blood pressure (mmHg) | 53 | 118 (110 - 127) | 84 - 145 |
| Diastolic blood pressure (mmHg) | 53 | 71 (65 - 76) | 50 - 103 |
| Number of drugs (n) | 53 | 1 (1 - 2) | 1 - 6 |
| Cardiovascular disease | 53 | 2 (3.8%) |  |
| Chronic pulmonary disease | 53 | 4 (7.5%) |  |
| Cancer | 53 | 5 (9.4%) |  |
| Diabetes | 53 | 0 (0%) |  |
| Hypertension | 53 | 7 (13.2%) |  |
| Hypercholesterolemia | 53 | 3 (5.7%) |  |
| Depression | 53 | 10 (18.9%) |  |
| Osteoarthritis | 53 | 1 (1.9%) |  |
| Walking aid | 53 | 0 (0%) |  |
| MVPA (min/d) | 53 | 23.2 (7.8 - 37.7) | 3.4 - 73.1 |
| SB (min/d) | 53 | 421.5 (352.5 - 486.2) | 110.4 - 735.5 |
| Gait speed (m/s) | 53 | 1.4 (1.2 - 1.5) | 0.9 - 1.7 |
| Handgrip strength (kg) | 53 | 30 (28 - 34) | 19.5 - 43 |
| 5-time sit-to-stand (n) | 53 | 8.2 (7 - 9.1) | 4.7 - 13 |
| V̇O_2_max (ml/kg/min) | 14 | 25 (23 - 30) | 21 - 33 |
| Isokinetic Strength (kg) | 14 | 94 (70 - 110) | 61 - 128 |
| Mini mental state examination score | 53 | 30 (28 - 30) | 23 - 30 |
| Short physical performance battery score | 52 | 12 (12 - 12) | 11 - 12 |

Continuous variables are presented as median (Q1-Q3) along with minimum and maximum values. Categorical variables are presented as n (%).

| **Supplementary Table 1c. Characteristics of the participants according to each decade in women (6/8)** | | | |
| --- | --- | --- | --- |
|  | **60-69** | | |
| **Variable (unit)** | **Sample size** | **Statistic** | **min-max** |
| Age (years) | 61 | 64 (62 - 66) | 60 - 69 |
| Education | 61 | 61 (100%) |  |
| No education |  | 0 (0%) |  |
| Primary school certificate |  | 0 (0%) |  |
| Secondary education diploma |  | 5 (8.2%) |  |
| High school diploma |  | 12 (19.7%) |  |
| University degree |  | 44 (72.1%) |  |
| Socio-professional category | 61 | 61 (100%) |  |
| Farmers |  | 0 (0%) |  |
| Artisans, shopkeepers, CEOs |  | 2 (3.3%) |  |
| Executives and intellectual professionals |  | 17 (27.9%) |  |
| Intermediate professions |  | 22 (36.1%) |  |
| Employees |  | 15 (24.6%) |  |
| Workers |  | 0 (0%) |  |
| Unemployed |  | 3 (4.9%) |  |
| Others |  | 2 (3.3%) |  |
| Family Income | 61 | 61 (100%) |  |
| < 1000 € - 1500 € |  | 5 (8.2%) |  |
| 1500 € - 2800 € |  | 18 (29.5%) |  |
| 2800 € - 4200+ € |  | 37 (60.7%) |  |
| Cannot or do not wish to answer |  | 1 (1.6%) |  |
| Height (m) | 61 | 1.6 (1.6 - 1.6) | 1.5 - 1.7 |
| Body weight (kg) | 61 | 62 (55 - 68) | 42.5 - 92 |
| Body mass index (kg/m²) | 61 | 23.5 (20.9 - 26.6) | 17.9 - 36.4 |
| Waist-to-hip ratio | 61 | 0.8 (0.8 - 0.9) | 0.6 - 1 |
| Heart rate (bpm) | 61 | 68 (62 - 74) | 50 - 98 |
| Systolic blood pressure (mmHg) | 61 | 127 (115 - 136) | 98 - 180 |
| Diastolic blood pressure (mmHg) | 61 | 71 (62 - 76) | 53 - 112 |
| Number of drugs (n) | 61 | 1 (1 - 2) | 1 - 15 |
| Cardiovascular disease | 61 | 9 (14.8%) |  |
| Chronic pulmonary disease | 61 | 1 (1.6%) |  |
| Cancer | 61 | 1 (1.6%) |  |
| Diabetes | 61 | 0 (0%) |  |
| Hypertension | 61 | 7 (11.5%) |  |
| Hypercholesterolemia | 61 | 10 (16.4%) |  |
| Depression | 61 | 10 (16.4%) |  |
| Osteoarthritis | 61 | 6 (9.8%) |  |
| Walking aid | 59 | 0 (0%) |  |
| MVPA (min/d) | 61 | 25.5 (12.5 - 41.8) | 0.4 - 126.1 |
| SB (min/d) | 61 | 345 (301.9 - 436) | 167.6 - 560.1 |
| Gait speed (m/s) | 61 | 1.3 (1.2 - 1.4) | 0.9 - 1.6 |
| Handgrip strength (kg) | 61 | 29 (24.5 - 32) | 8 - 35 |
| 5-time sit-to-stand (n) | 61 | 9.1 (7.7 - 10.5) | 6 - 17 |
| V̇O_2_max (ml/kg/min) | 27 | 22 (19 - 26) | 17 - 31 |
| Isokinetic Strength (kg) | 27 | 78 (61 - 99) | 47 - 114 |
| Mini mental state examination score | 61 | 29 (28 - 30) | 24 - 30 |
| Short physical performance battery score | 60 | 12 (12 - 12) | 9 - 12 |

Continuous variables are presented as median (Q1-Q3) along with minimum and maximum values. Categorical variables are presented as n (%).

| **Supplementary Table 1c. Characteristics of the participants according to each decade in women (7/8)** | | | |
| --- | --- | --- | --- |
|  | **70-80** | | |
| **Variable (unit)** | **Sample size** | **Statistic** | **min-max** |
| Age (years) | 60 | 73 (71 - 76) | 70 - 79 |
| Education | 60 | 60 (100%) |  |
| No education |  | 0 (0%) |  |
| Primary school certificate |  | 3 (5%) |  |
| Secondary education diploma |  | 7 (11.7%) |  |
| High school diploma |  | 20 (33.3%) |  |
| University degree |  | 30 (50%) |  |
| Socio-professional category | 60 | 60 (100%) |  |
| Farmers |  | 0 (0%) |  |
| Artisans, shopkeepers, CEOs |  | 3 (5%) |  |
| Executives and intellectual professionals |  | 13 (21.7%) |  |
| Intermediate professions |  | 17 (28.3%) |  |
| Employees |  | 20 (33.3%) |  |
| Workers |  | 3 (5%) |  |
| Unemployed |  | 1 (1.7%) |  |
| Others |  | 3 (5%) |  |
| Family Income | 60 | 60 (100%) |  |
| < 1000 € - 1500 € |  | 6 (10%) |  |
| 1500 € - 2800 € |  | 20 (33.3%) |  |
| 2800 € - 4200+ € |  | 32 (53.3%) |  |
| Cannot or do not wish to answer |  | 2 (3.3%) |  |
| Height (m) | 60 | 1.6 (1.6 - 1.6) | 1.4 - 1.7 |
| Body weight (kg) | 59 | 62 (55 - 66) | 45 - 92 |
| Body mass index (kg/m²) | 59 | 24.1 (21.7 - 27.2) | 18.8 - 36.6 |
| Waist-to-hip ratio | 60 | 0.8 (0.8 - 0.9) | 0.7 - 1.1 |
| Heart rate (bpm) | 60 | 68.5 (63 - 73) | 48 - 94 |
| Systolic blood pressure (mmHg) | 60 | 138.5 (121.5 - 153) | 108 - 184 |
| Diastolic blood pressure (mmHg) | 60 | 72 (63.5 - 77.5) | 54 - 102 |
| Number of drugs (n) | 60 | 2 (1 - 4.5) | 1 - 14 |
| Cardiovascular disease | 60 | 13 (21.7%) |  |
| Chronic pulmonary disease | 60 | 4 (6.7%) |  |
| Cancer | 60 | 8 (13.3%) |  |
| Diabetes | 60 | 3 (5%) |  |
| Hypertension | 60 | 22 (36.7%) |  |
| Hypercholesterolemia | 60 | 15 (25%) |  |
| Depression | 60 | 16 (26.7%) |  |
| Osteoarthritis | 60 | 7 (11.7%) |  |
| Walking aid | 59 | 0 (0%) |  |
| MVPA (min/d) | 60 | 18.9 (8.4 - 30.8) | 0.9 - 57.6 |
| SB (min/d) | 60 | 348.7 (289.4 - 407.2) | 120.8 - 692.5 |
| Gait speed (m/s) | 60 | 1.2 (1.1 - 1.3) | 0.7 - 1.9 |
| Handgrip strength (kg) | 60 | 24 (20 - 27) | 12 - 37 |
| 5-time sit-to-stand (n) | 60 | 9.6 (8 - 10.6) | 6 - 16 |
| V̇O_2_max (ml/kg/min) | 28 | 19.5 (16 - 22) | 14 - 30 |
| Isokinetic Strength (kg) | 27 | 71 (59 - 90) | 45 - 177 |
| Mini mental state examination score | 59 | 29 (28 - 30) | 20 - 30 |
| Short physical performance battery score | 58 | 12 (12 - 12) | 9 - 12 |

Continuous variables are presented as median (Q1-Q3) along with minimum and maximum values. Categorical variables are presented as n (%).

| **Supplementary Table 1c. Characteristics of the participants according to each decade in women (8/8)** | | | |
| --- | --- | --- | --- |
|  | **80+** | | |
| **Variable (unit)** | **Sample size** | **Statistic** | **min-max** |
| Age (years) | 38 | 83 (82 - 86) | 80 - 92 |
| Education | 38 | 38 (100%) |  |
| No education |  | 0 (0%) |  |
| Primary school certificate |  | 6 (15.8%) |  |
| Secondary education diploma |  | 8 (21.1%) |  |
| High school diploma |  | 5 (13.2%) |  |
| University degree |  | 19 (50%) |  |
| Socio-professional category | 38 | 38 (100%) |  |
| Farmers |  | 1 (2.6%) |  |
| Artisans, shopkeepers, CEOs |  | 2 (5.3%) |  |
| Executives and intellectual professionals |  | 5 (13.2%) |  |
| Intermediate professions |  | 14 (36.8%) |  |
| Employees |  | 14 (36.8%) |  |
| Workers |  | 0 (0%) |  |
| Unemployed |  | 1 (2.6%) |  |
| Others |  | 1 (2.6%) |  |
| Family Income | 38 | 38 (100%) |  |
| < 1000 € - 1500 € |  | 4 (10.5%) |  |
| 1500 € - 2800 € |  | 20 (52.6%) |  |
| 2800 € - 4200+ € |  | 10 (26.3%) |  |
| Cannot or do not wish to answer |  | 4 (10.5%) |  |
| Height (m) | 38 | 1.6 (1.5 - 1.6) | 1.3 - 1.7 |
| Body weight (kg) | 38 | 58.3 (50 - 64.2) | 39 - 83 |
| Body mass index (kg/m²) | 38 | 23.5 (21.1 - 26.4) | 17.3 - 36.2 |
| Waist-to-hip ratio | 38 | 0.9 (0.8 - 0.9) | 0.7 - 1.1 |
| Heart rate (bpm) | 38 | 71 (63 - 83) | 55 - 90 |
| Systolic blood pressure (mmHg) | 38 | 143 (132 - 155) | 103 - 185 |
| Diastolic blood pressure (mmHg) | 38 | 70.5 (63 - 78) | 56 - 97 |
| Number of drugs (n) | 38 | 4 (2 - 6) | 1 - 11 |
| Cardiovascular disease | 38 | 13 (34.2%) |  |
| Chronic pulmonary disease | 38 | 3 (7.9%) |  |
| Cancer | 38 | 10 (26.3%) |  |
| Diabetes | 38 | 1 (2.6%) |  |
| Hypertension | 38 | 22 (57.9%) |  |
| Hypercholesterolemia | 38 | 9 (23.7%) |  |
| Depression | 38 | 6 (15.8%) |  |
| Osteoarthritis | 38 | 8 (21.1%) |  |
| Walking aid | 38 | 5 (13.2%) |  |
| MVPA (min/d) | 38 | 14.4 (4.7 - 28) | 0 - 65.6 |
| SB (min/d) | 38 | 385.2 (313.1 - 439.7) | 176.2 - 680 |
| Gait speed (m/s) | 38 | 1.1 (0.9 - 1.3) | 0.4 - 1.6 |
| Handgrip strength (kg) | 38 | 20 (16 - 24) | 6 - 32 |
| 5-time sit-to-stand (n) | 37 | 9.8 (8.4 - 11.4) | 5.5 - 14.9 |
| V̇O_2_max (ml/kg/min) | 6 | 17 (17 - 21) | 14 - 23 |
| Isokinetic Strength (kg) | 6 | 35 (32 - 40) | 29 - 70 |
| Mini mental state examination score | 38 | 29 (28 - 30) | 16 - 30 |
| Short physical performance battery score | 38 | 11.5 (10 - 12) | 2 - 12 |

Continuous variables are presented as median (Q1-Q3) along with minimum and maximum values. Categorical variables are presented as n (%).

Supplementary Table 2. Contrast analyses on the independent and interactive associations of age, MVPA and SB with physical function and performance across the adult lifespan (1/2)

| **I.V** | **Age range of significance†** | **B** | **S.E** | **p** |
| --- | --- | --- | --- | --- |
| **D.V: Gait speed (m/s) - N = 486** | | | | |
| **age** | [20, 43] / [58, 92] | [7E-03, 2E-03] / [-2E-03, -1E-02] | [2E-03, 9E-04] / [7E-04, 3E-03] | [0.001, 0.034] / [0.028, 0.000] |
| **age²** | constant | -1E-04 | 3E-05 | 0.000 |
| **MVPA** | [80, 92] | [2E-03, 4E-03] | [8E-04, 2E-03] | [0.042, 0.015] |
| **MVPA × age** | [64, 92] | [6E-05, 2E-04] | [3E-05, 9E-05] | [0.049, 0.023] |
| **MVPA × age²** | constant | 3E-06 | 1E-06 | 0.032 |
| **SB** | [54, 71] | [-3E-04, -2E-04] | [1E-04, 1E-04] | [0.048, 0.045] |
| **SB × age** | n.s | n.s | n.s | n.s |
| **SB × age²** | n.s | n.s | n.s | n.s |
| **MVPA × SB** | n.s | n.s | n.s | n.s |
| **MVPA × SB × age** | n.s | n.s | n.s | n.s |
| **MVPA × SB × age²** | n.s | n.s | n.s | n.s |
| **D.V: Handgrip strength (square root of kg) - N = 494** | | | | |
| **age** | [42, 92] | [-5E-03, -3E-02] | [3E-03, 7E-03] | [0.037, 0.000] |
| **age²** | constant | -2E-04 | 9E-05 | 0.012 |
| **MVPA** | n.s | n.s | n.s | n.s |
| **MVPA × age** | n.s | n.s | n.s | n.s |
| **MVPA × age²** | n.s | n.s | n.s | n.s |
| **SB** | n.s | n.s | n.s | n.s |
| **SB × age** | n.s | n.s | n.s | n.s |
| **SB × age²** | n.s | n.s | n.s | n.s |
| **MVPA × SB** | [77, 92] | [-3E-05, -9E-05] | [1E-05, 3E-05] | [0.049, 0.006] |
| **MVPA × SB × age** | [20, 21] / [64, 92] | [5E-06, 5E-06] / [-1E-06, -5E-06] | [2E-06, 2E-06] / [7E-07, 2E-06] | [0.048, 0.049] / [0.044, 0.016] |
| **MVPA × SB × age²** | constant | -7E-08 | 3E-08 | 0.024 |

**†**Numbers within square brackets represent the age range (lower and upper limit) in which the I.V was significant, along with the corresponding B, S.E and p values.
D.V: Dependent variable; I.V: Independent variable; MVPA: Moderate to vigorous physical activity (min/d); n.s: Not significant; SB: Sedentary behavior (min/d); S.E: Standard error
*The effects of the single terms (age, age², MVPA, SB) and second order interaction terms (age × MVPA, age × SB, age² × MVPA, age² × SB) were computed while fixing any moderators (either MVPA, SB or both) at their median levels.

Supplementary Table 2. Contrast analyses on the independent and interactive associations of age, MVPA and SB with physical function and performance across the adult lifespan (2/2)

| **I.V** | **Age range of significance†** | **B** | **S.E** | **p** |
| --- | --- | --- | --- | --- |
| **D.V: 5-time chair-rise (square root of s) - N = 491** | | | | |
| **age** | constant | 9E-03 | 1E-03 | 0.000 |
| **MVPA** | [58, 92] | [-2E-03, -5E-03] | [8E-04, 2E-03] | [0.039, 0.005] |
| **MVPA × age** | constant | -9E-05 | 4E-05 | 0.043 |
| **SB** | [20, 36] | [-7E-04, -5E-04] | [3E-04, 2E-04] | [0.035, 0.048] |
| **SB × age** | constant | 1E-05 | 7E-06 | 0.045 |
| **MVPA × SB** | n.s | n.s | n.s | n.s |
| **MVPA × SB × age** | n.s | n.s | n.s | n.s |
| **D.V: V̇O_2_max (square root of ml/kg/min) - N = 162** | | | | |
| **age** | constant | -2E-02 | 3E-03 | 0.000 |
| **MVPA** | [21, 70] | [3E-02, 5E-03] | [7E-03, 3E-03] | [0.000, 0.046] |
| **MVPA × age** | constant | -5E-04 | 2E-04 | 0.003 |
| **SB** | n.s | n.s | n.s | n.s |
| **SB × age** | n.s | n.s | n.s | n.s |
| **MVPA × SB** | n.s | n.s | n.s | n.s |
| **MVPA × SB × age** | n.s | n.s | n.s | n.s |
| **D.V: Isokinetic strength (square root of N⋅m) - N = 165** | | | | |
| **age** | constant | -4E-02 | 1E-02 | 0.000 |
| **MVPA** | n.s | n.s | n.s | n.s |
| **MVPA × age** | n.s | n.s | n.s | n.s |
| **SB** | [21, 21] / [87, 87] | [6E-03, 6E-03] / [-4E-03, -4E-03] | [3E-03, 3E-03] / [2E-03, 2E-03] | [0.050, 0.050] / [0.048, 0.048] |
| **SB × age** | constant | -1E-04 | 6E-05 | 0.025 |
| **MVPA × SB** | n.s | n.s | n.s | n.s |
| **MVPA × SB × age** | n.s | n.s | n.s | n.s |

**†**Numbers within square brackets represent the age range (lower and upper limit) in which the I.V was significant, along with the corresponding B, S.E and p values.
D.V: Dependent variable; I.V: Independent variable; MVPA: Moderate to vigorous physical activity (min/d); n.s: Not significant; SB: Sedentary behavior (min/d); S.E: Standard error
*The effects of the single terms (age, age², MVPA, SB) and second order interaction terms (age × MVPA, age × SB, age² × MVPA, age² × SB) were computed while fixing any moderators (either MVPA, SB or both) at their median levels.

**Supplementary Table 3a.** **Sensitivity analyses on the independent and interactive associations of age, MVPA and SB with VO2max and lower limb isokinetic strength across the adult lifespan after removing individuals with cardiovascular disease.**

|  | **Isokinetic strength (square root of N⋅m) N = 150** | | |  | **V̇O_2_max (square root of ml/kg/min) N = 148** | | |
| --- | --- | --- | --- | --- | --- | --- | --- |
| **I.V*** | **B** | **S.E** | **p** |  | **B** | **S.E** | **p** |
| **age** | -4E-02 | 1E-02 | **<0.001** |  | -2E-02 | 4E-03 | **<0.001** |
| **MVPA** | 3E-03 | 2E-02 | 0.894 |  | 3E-02 | 8E-03 | **<0.001** |
| **MVPA × age** | -9E-05 | 5E-04 | 0.854 |  | -5E-04 | 2E-04 | **0.013** |
| **SB** | 6E-03 | 3E-03 | **0.050** |  | 3E-04 | 1E-03 | 0.794 |
| **SB × age** | -1E-04 | 6E-05 | **0.025** |  | -5E-06 | 2E-05 | 0.825 |
| **MVPA × SB** | -2E-04 | 2E-04 | 0.278 |  | -1E-04 | 7E-05 | 0.065 |
| **MVPA × SB × age** | 4E-06 | 4E-06 | 0.389 |  | 3E-06 | 2E-06 | 0.088 |

I.V: Independent variable; MVPA: Moderate to vigorous physical activity (min/d); SB: Sedentary behavior (min/d); S.E: Standard error
*The effects of the single terms (age, MVPA, SB) and second order interaction terms (age × MVPA, age × SB) were computed while fixing any moderators (either MVPA, SB or both) at their median levels. In addition, the effects of MVPA, SB, and MVPA × SB were provided for the age of 21.

Supplementary Table 3b. Independent and interactive associations of age, MVPA and SB with physical function and performance across the adult lifespan in men

|  | **Gait speed  (m/s) N = 177** | | |  | **Handgrip strength (square root of kg) N = 182** | | |  | **5-time chair-rise (square root of s) N = 180** | | |  | **Isokinetic strength (square root of N⋅m) N = 71** | | |  | **V̇O_2_max (square root of ml/kg/min) N = 67** | | |
| --- | --- | --- | --- | --- | --- | --- | --- | --- | --- | --- | --- | --- | --- | --- | --- | --- | --- | --- | --- |
| **I.V*** | **B** | **S.E** | **p** |  | **B** | **S.E** | **p** |  | **B** | **S.E** | **p** |  | **B** | **S.E** | **p** |  | **B** | **S.E** | **p** |
| **age** | 5E-03 | 4E-03 | 0.222 |  | 2E-02 | 1E-02 | 0.211 |  | 8E-03 | 2E-03 | **<0.001** |  | -4E-02 | 2E-02 | **0.025** |  | -2E-02 | 6E-03 | **0.003** |
| **age²** | -9E-05 | 6E-05 | 0.142 |  | -4E-04 | 2E-04 | **0.035** |  | - | - | - |  | - | - | - |  | - | - | - |
| **MVPA** | 2E-03 | 3E-03 | 0.477 |  | -2E-03 | 1E-02 | 0.876 |  | 6E-03 | 3E-03 | 0.079 |  | -2E-02 | 3E-02 | 0.540 |  | 4E-02 | 1E-02 | **<0.001** |
| **MVPA × age** | -2E-04 | 2E-04 | 0.201 |  | -2E-04 | 6E-04 | 0.748 |  | -2E-04 | 7E-05 | **0.015** |  | 5E-04 | 7E-04 | 0.519 |  | -8E-04 | 3E-04 | **0.006** |
| **MVPA × age²** | 4E-06 | 2E-06 | 0.086 |  | 4E-06 | 7E-06 | 0.603 |  | - | - | - |  | - | - | - |  | - | - | - |
| **SB** | 4E-04 | 4E-04 | 0.430 |  | -4E-04 | 1E-03 | 0.745 |  | -1E-03 | 5E-04 | **0.019** |  | 4E-03 | 5E-03 | 0.418 |  | 2E-03 | 2E-03 | 0.243 |
| **SB × age** | -2E-05 | 3E-05 | 0.558 |  | -1E-05 | 1E-04 | 0.921 |  | 2E-05 | 1E-05 | **0.040** |  | -7E-05 | 1E-04 | 0.533 |  | -3E-05 | 4E-05 | 0.435 |
| **SB × age²** | 2E-07 | 5E-07 | 0.717 |  | 3E-07 | 1E-06 | 0.827 |  | - | - | - |  | - | - | - |  | - | - | - |
| **MVPA × SB** | 3E-05 | 3E-05 | 0.409 |  | -1E-05 | 1E-04 | 0.889 |  | -3E-05 | 3E-05 | 0.242 |  | -3E-04 | 3E-04 | 0.378 |  | -3E-04 | 1E-04 | **0.012** |
| **MVPA × SB × age** | -1E-06 | 2E-06 | 0.524 |  | 3E-06 | 5E-06 | 0.610 |  | 4E-07 | 6E-07 | 0.469 |  | 6E-06 | 9E-06 | 0.474 |  | 7E-06 | 3E-06 | **0.030** |
| **MVPA × SB × age²** | 1E-08 | 2E-08 | 0.622 |  | -5E-08 | 6E-08 | 0.467 |  | - | - | - |  | - | - | - |  | - | - | - |

I.V: Independent variable; MVPA: Moderate to vigorous physical activity (min/d); SB: Sedentary behavior (min/d); S.E: Standard error
*The effects of the single terms (age, MVPA, SB) and second order interaction terms (age × MVPA, age × SB) were computed while fixing any moderators (either MVPA, SB or both) at their median levels. In addition, the effects of MVPA, SB, and MVPA × SB were provided for the lower limit of age (20 for gait speed, handgrip strength and chair-rise test; and 25 for Isokinetic strength and V̇O_2_max). The same limits were chosen to compute the effects of age, age × MVPA, age × SB, and age × MVPA × SB in quadratic models (Gait speed, and handgrip strength).

Supplementary Table 3c. Independent and interactive associations of age, MVPA and SB with physical function and performance across the adult lifespan in women

|  | **Gait speed  (m/s) N = 309** | | |  | **Handgrip strength (square root of kg) N = 312** | | |  | **5-time chair-rise (square root of s) N = 311** | | |  | **Isokinetic strength (square root of N⋅m) N = 94** | | |  | **V̇O_2_max (square root of ml/kg/min) N = 95** | | |
| --- | --- | --- | --- | --- | --- | --- | --- | --- | --- | --- | --- | --- | --- | --- | --- | --- | --- | --- | --- |
| **I.V*** | **B** | **S.E** | **p** |  | **B** | **S.E** | **p** |  | **B** | **S.E** | **p** |  | **B** | **S.E** | **p** |  | **B** | **S.E** | **p** |
| **age** | 7E-03 | 3E-03 | **0.007** |  | 1E-03 | 7E-03 | 0.867 |  | 9E-03 | 1E-03 | **<0.001** |  | -3E-02 | 1E-02 | **0.023** |  | -2E-02 | 5E-03 | **<0.001** |
| **age²** | -1E-04 | 4E-05 | **0.002** |  | -2E-04 | 1E-04 | 0.080 |  | - | - | - |  | - | - | - |  | - | - | - |
| **MVPA** | 1E-03 | 2E-03 | 0.495 |  | 7E-03 | 5E-03 | 0.144 |  | 1E-03 | 2E-03 | 0.588 |  | 1E-02 | 3E-02 | 0.640 |  | 1E-02 | 1E-02 | 0.188 |
| **MVPA × age** | -7E-05 | 1E-04 | 0.572 |  | -1E-04 | 3E-04 | 0.682 |  | -6E-05 | 6E-05 | 0.274 |  | -4E-04 | 8E-04 | 0.564 |  | -2E-04 | 2E-04 | 0.438 |
| **MVPA × age²** | 8E-07 | 2E-06 | 0.638 |  | -7E-07 | 4E-06 | 0.882 |  | - | - | - |  | - | - | - |  | - | - | - |
| **SB** | -6E-05 | 4E-04 | 0.864 |  | -2E-03 | 9E-04 | 0.082 |  | -2E-04 | 4E-04 | 0.647 |  | 6E-03 | 4E-03 | 0.099 |  | -3E-04 | 1E-03 | 0.825 |
| **SB × age** | -2E-05 | 2E-05 | 0.473 |  | 8E-05 | 6E-05 | 0.178 |  | 7E-06 | 1E-05 | 0.493 |  | -2E-04 | 8E-05 | 0.068 |  | 1E-06 | 3E-05 | 0.958 |
| **SB × age²** | 3E-07 | 3E-07 | 0.396 |  | -9E-07 | 8E-07 | 0.273 |  | - | - | - |  | - | - | - |  | - | - | - |
| **MVPA × SB** | 5E-06 | 2E-05 | 0.798 |  | -8E-05 | 5E-05 | 0.103 |  | 6E-05 | 2E-05 | **0.007** |  | -1E-04 | 2E-04 | 0.542 |  | -1E-05 | 7E-05 | 0.861 |
| **MVPA × SB × age** | 3E-07 | 1E-06 | 0.810 |  | 6E-06 | 3E-06 | **0.044** |  | -2E-06 | 6E-07 | **0.001** |  | 3E-06 | 5E-06 | 0.634 |  | 2E-07 | 2E-06 | 0.907 |
| **MVPA × SB × age²** | -5E-09 | 2E-08 | 0.749 |  | -1E-07 | 4E-08 | **0.019** |  | - | - | - |  | - | - | - |  | - | - | - |

D.V: Dependent variable; MVPA: Moderate to vigorous physical activity (min/d); SB: Sedentary behavior (min/d); S.E: Standard error
*The effects of the single terms (age, MVPA, SB) and second order interaction terms (age × MVPA, age × SB) were computed while fixing any moderators (either MVPA, SB or both) at their median levels. In addition, the effects of MVPA, SB, and MVPA × SB were provided for the lower limit of age (20 for gait speed, handgrip strength and chair-rise test; and 21 for Isokinetic strength and V̇O_2_max). The same limits were chosen to compute the effects of age, age × MVPA, age × SB, and age × MVPA × SB in quadratic models (Gait speed, and handgrip strength).

Supplementary Table 3d. Contrast analyses on the independent and interactive associations of age, MVPA and SB with physical function and performance across the adult lifespan in men (1/2)

| **I.V** | **Age range of significance†** | **B** | **S.E** | **p** |
| --- | --- | --- | --- | --- |
| **D.V: Gait speed (m/s) - N = 177** | | | | |
| **age** | n.s | n.s | n.s | n.s |
| **age²** | n.s | n.s | n.s | n.s |
| **MVPA** | [78, 90] | [2E-03, 6E-03] | [1E-03, 2E-03] | [0.042, 0.012] |
| **MVPA × age** | [61, 90] | [1E-04, 3E-04] | [5E-05, 2E-04] | [0.042, 0.037] |
| **MVPA × age²** | n.s | n.s | n.s | n.s |
| **SB** | n.s | n.s | n.s | n.s |
| **SB × age** | n.s | n.s | n.s | n.s |
| **SB × age²** | constant | 2E-07 | 2E-07 | 0.000 |
| **MVPA × SB** | n.s | n.s | n.s | n.s |
| **MVPA × SB × age** | n.s | n.s | n.s | n.s |
| **MVPA × SB × age²** | n.s | n.s | n.s | n.s |
| **D.V: Handgrip strength (square root of kg) - N = 182** | | | | |
| **age** | [51, 90] | [-8E-03, -4E-02] | [4E-03, 1E-02] | [0.043, 0.008] |
| **age²** | constant | -4E-04 | 2E-04 | 0.035 |
| **MVPA** | n.s | n.s | n.s | n.s |
| **MVPA × age** | n.s | n.s | n.s | n.s |
| **MVPA × age²** | n.s | n.s | n.s | n.s |
| **SB** | n.s | n.s | n.s | n.s |
| **SB × age** | n.s | n.s | n.s | n.s |
| **SB × age²** | n.s | n.s | n.s | n.s |
| **MVPA × SB** | n.s | n.s | n.s | n.s |
| **MVPA × SB × age** | n.s | n.s | n.s | n.s |
| **MVPA × SB × age²** | n.s | n.s | n.s | n.s |

**†**Numbers within square brackets represent the age range (lower and upper limit) in which the I.V was significant, along with the corresponding B, S.E and p values.
D.V: Dependent variable; I.V: Independent variable; MVPA: Moderate to vigorous physical activity (min/d); n.s: Not significant; SB: Sedentary behavior (min/d); S.E: Standard error
*The effects of the single terms (age, age², MVPA, SB) and second order interaction terms (age × MVPA, age × SB, age² × MVPA, age² × SB) were computed while fixing any moderators (either MVPA, SB or both) at their median levels.

Supplementary Table 3d. Contrast analyses on the independent and interactive associations of age, MVPA and SB with physical function and performance across the adult lifespan in men (2/2)

| **I.V** | **Age range of significance†** | **B** | **S.E** | **p** |
| --- | --- | --- | --- | --- |
| **D.V: 5-time chair-rise (square root of s) - N = 180** | | | | |
| **age** | constant | 8E-03 | 2E-03 | 0.000 |
| **MVPA** | [69, 90] | [-3E-03, -7E-03] | [1E-03, 2E-03] | [0.044, 0.008] |
| **MVPA × age** | constant | -2E-04 | 7E-05 | 0.015 |
| **SB** | [20, 51] | [-1E-03, -5E-04] | [5E-04, 3E-04] | [0.019, 0.046] |
| **SB × age** | constant | 2E-05 | 1E-05 | 0.040 |
| **MVPA × SB** | n.s | n.s | n.s | n.s |
| **MVPA × SB × age** | n.s | n.s | n.s | n.s |
| **D.V: V̇O_2_max (square root of ml/kg/min) - N = 67** | | | | |
| **age** | constant | -2E-02 | 6E-03 | 0.003 |
| **MVPA** | [25, 67] | [4E-02, 9E-03] | [1E-02, 4E-03] | [0.000, 0.034] |
| **MVPA × age** | constant | -8E-04 | 3E-04 | 0.006 |
| **SB** | n.s | n.s | n.s | n.s |
| **SB × age** | n.s | n.s | n.s | n.s |
| **MVPA × SB** | [25, 60] | [-3E-04, -8E-05] | [1E-04, 4E-05] | [0.012, 0.041] |
| **MVPA × SB × age** | constant | 7E-06 | 3E-06 | 0.030 |
| **D.V: Isokinetic strength (square root of N⋅m) - N = 71** | | | | |
| **age** | constant | -4E-02 | 2E-02 | 0.025 |
| **MVPA** | n.s | n.s | n.s | n.s |
| **MVPA × age** | n.s | n.s | n.s | n.s |
| **SB** | n.s | n.s | n.s | n.s |
| **SB × age** | n.s | n.s | n.s | n.s |
| **MVPA × SB** | n.s | n.s | n.s | n.s |
| **MVPA × SB × age** | n.s | n.s | n.s | n.s |

**†**Numbers within square brackets represent the age range (lower and upper limit) in which the I.V was significant, along with the corresponding B, S.E and p values.
D.V: Dependent variable; I.V: Independent variable; MVPA: Moderate to vigorous physical activity (min/d); n.s: Not significant; SB: Sedentary behavior (min/d); S.E: Standard error
*The effects of the single terms (age, age², MVPA, SB) and second order interaction terms (age × MVPA, age × SB, age² × MVPA, age² × SB) were computed while fixing any moderators (either MVPA, SB or both) at their median levels.

Supplementary Table 3e. Contrast analyses on the independent and interactive associations of age, MVPA and SB with physical function and performance across the adult lifespan in women (1/2)

| **I.V** | **Age range of significance†** | **B** | **S.E** | **p** |
| --- | --- | --- | --- | --- |
| **D.V: Gait speed (m/s) - N = 309** | | | | |
| **age** | [20, 39] / [58, 92] | [7E-03, 3E-03] / [-2E-03, -1E-02] | [3E-03, 1E-03] / [9E-04, 3E-03] | [0.007, 0.043] / [0.041, 0.002] |
| **age²** | constant | -1E-04 | 4E-05 | 0.002 |
| **MVPA** | n.s | n.s | n.s | n.s |
| **MVPA × age** | n.s | n.s | n.s | n.s |
| **MVPA × age²** | n.s | n.s | n.s | n.s |
| **SB** | n.s | n.s | n.s | n.s |
| **SB × age** | n.s | n.s | n.s | n.s |
| **SB × age²** | n.s | n.s | n.s | n.s |
| **MVPA × SB** | n.s | n.s | n.s | n.s |
| **MVPA × SB × age** | n.s | n.s | n.s | n.s |
| **MVPA × SB × age²** | n.s | n.s | n.s | n.s |
| **D.V: Handgrip strength (square root of kg) - N = 312** | | | | |
| **age** | [41, 92] | [-6E-03, -2E-02] | [3E-03, 8E-03] | [0.043, 0.004] |
| **age²** | n.s | n.s | n.s | n.s |
| **MVPA** | [29, 42] | [6E-03, 4E-03] | [3E-03, 2E-03] | [0.046, 0.048] |
| **MVPA × age** | [52, 57] | [-2E-04, -2E-04] | [8E-05, 9E-05] | [0.044, 0.047] |
| **MVPA × age²** | n.s | n.s | n.s | n.s |
| **SB** | n.s | n.s | n.s | n.s |
| **SB × age** | n.s | n.s | n.s | n.s |
| **SB × age²** | n.s | n.s | n.s | n.s |
| **MVPA × SB** | [72, 92] | [-4E-05, -2E-04] | [2E-05, 6E-05] | [0.041, 0.004] |
| **MVPA × SB × age** | [20, 23] / [59, 92] | [6E-06, 5E-06] / [-2E-06, -9E-06] | [3E-06, 3E-06] / [9E-07, 3E-06] | [0.044, 0.049] / [0.040, 0.012] |
| **MVPA × SB × age²** | constant | -1E-07 | 4E-08 | 0.019 |

**†**Numbers within square brackets represent the age range (lower and upper limit) in which the I.V was significant, along with the corresponding B, S.E and p values.
D.V: Dependent variable; I.V: Independent variable; MVPA: Moderate to vigorous physical activity (min/d); n.s: Not significant; SB: Sedentary behavior (min/d); S.E: Standard error
*The effects of the single terms (age, age², MVPA, SB) and second order interaction terms (age × MVPA, age × SB, age² × MVPA, age² × SB) were computed while fixing any moderators (either MVPA, SB or both) at their median levels.

Supplementary Table 3e. Contrast analyses on the independent and interactive associations of age, MVPA and SB with physical function and performance across the adult lifespan in women (2/2)

| **I.V** | **Age range of significance†** | **B** | **S.E** | **p** |
| --- | --- | --- | --- | --- |
| **D.V: 5-time chair-rise (square root of s) - N = 311** | | | | |
| **age** | constant | 9E-03 | 1E-03 | 0.000 |
| **MVPA** | n.s | n.s | n.s | n.s |
| **MVPA × age** | n.s | n.s | n.s | n.s |
| **SB** | n.s | n.s | n.s | n.s |
| **SB × age** | n.s | n.s | n.s | n.s |
| **MVPA × SB** | [20, 39] / [67, 92] | [6E-05, 3E-05] / [-2E-05, -7E-05] | [2E-05, 1E-05] / [1E-05, 2E-05] | [0.007, 0.046] / [0.049, 0.002] |
| **MVPA × SB × age** | constant | -2E-06 | 6E-07 | 0.001 |
| **D.V: V̇O_2_max (square root of ml/kg/min) - N = 95** | | | | |
| **age** | constant | -2E-02 | 5E-03 | 0.000 |
| **MVPA** | [49, 60] | [8E-03, 6E-03] | [4E-03, 3E-03] | [0.049, 0.048] |
| **MVPA × age** | n.s | n.s | n.s | n.s |
| **SB** | n.s | n.s | n.s | n.s |
| **SB × age** | n.s | n.s | n.s | n.s |
| **MVPA × SB** | n.s | n.s | n.s | n.s |
| **MVPA × SB × age** | n.s | n.s | n.s | n.s |
| **D.V: Isokinetic strength (square root of N⋅m) - N = 94** | | | | |
| **age** | constant | -3E-02 | 1E-02 | 0.023 |
| **MVPA** | n.s | n.s | n.s | n.s |
| **MVPA × age** | n.s | n.s | n.s | n.s |
| **SB** | n.s | n.s | n.s | n.s |
| **SB × age** | n.s | n.s | n.s | n.s |
| **MVPA × SB** | n.s | n.s | n.s | n.s |
| **MVPA × SB × age** | n.s | n.s | n.s | n.s |

**†**Numbers within square brackets represent the age range (lower and upper limit) in which the I.V was significant, along with the corresponding B, S.E and p values.
D.V: Dependent variable; I.V: Independent variable; MVPA: Moderate to vigorous physical activity (min/d); n.s: Not significant; SB: Sedentary behavior (min/d); S.E: Standard error
*The effects of the single terms (age, age², MVPA, SB) and second order interaction terms (age × MVPA, age × SB, age² × MVPA, age² × SB) were computed while fixing any moderators (either MVPA, SB or both) at their median levels.

Supplementary Table 4a. Estimated marginal mean values of physical function and performance across the adult lifespan

| **Age** | **MEAN (95% CI)** | **Inter-decade difference (%)** |
| --- | --- | --- |
| **Gait speed (m/s)** | | |
| 20 | 1.14 (1.04, 1.24) |  |
| 30 | 1.2 (1.10, 1.30) | 5% |
| 40 | 1.24 (1.13, 1.34) | 3% |
| 50 | 1.25 (1.14, 1.36) | 1% |
| 60 | 1.24 (1.14, 1.34) | -1% |
| 70 | 1.21 (1.11, 1.31) | -2% |
| 80 | 1.15 (1.05, 1.25) | -5% |
| 90 | 1.07 (0.96, 1.18) | -7% |
| Average inter-decade difference* | | -4% |
| **Handgrip strength (kg)** | | |
| 20 | 36.03 (32.78, 39.45) |  |
| 30 | 36.27 (33.09, 39.60) | 1% |
| 40 | 36 (32.70, 39.45) | -1% |
| 50 | 35.22 (31.86, 38.74) | -2% |
| 60 | 33.94 (30.68, 37.36) | -4% |
| 70 | 32.2 (29.15, 35.41) | -5% |
| 80 | 30.04 (27.14, 33.09) | -7% |
| 90 | 27.5 (24.44, 30.75) | -8% |
| Average inter-decade difference* | | -4% |
| **5-time chair rise test (s)** | | |
| 20 | 7.52 ( 6.48, 8.63) |  |
| 30 | 8.01 ( 7.00, 9.08) | 7% |
| 40 | 8.51 ( 7.52, 9.56) | 6% |
| 50 | 9.03 ( 8.05, 10.07) | 6% |
| 60 | 9.56 ( 8.57, 10.60) | 6% |
| 70 | 10.11 ( 9.10, 11.18) | 6% |
| 80 | 10.67 ( 9.62, 11.79) | 6% |
| 90 | 11.25 (10.13, 12.43) | 5% |
| Average inter-decade difference* | | 6% |
| **V̇O_2_max (ml/kg/min)** | | |
| 20 | 32.54 (27.04, 38.55) |  |
| 30 | 30.49 (25.49, 35.94) | -6% |
| 40 | 28.51 (23.91, 33.51) | -6% |
| 50 | 26.6 (22.30, 31.27) | -7% |
| 60 | 24.75 (20.64, 29.22) | -7% |
| 70 | 22.97 (18.96, 27.35) | -7% |
| 80 | 21.25 (17.27, 25.65) | -7% |
| 90 | 19.6 (15.58, 24.09) | -8% |
| Average inter-decade difference* | | -7% |
| **Isokinetic strength (N-m)** | | |
| 20 | 152.19 (117.12, 191.85) |  |
| 30 | 142.8 (111.14, 178.43) | -6% |
| 40 | 133.72 (104.91, 166.01) | -6% |
| 50 | 124.93 ( 98.38, 154.64) | -7% |
| 60 | 116.43 ( 91.52, 144.34) | -7% |
| 70 | 108.24 ( 84.35, 135.10) | -7% |
| 80 | 100.35 ( 76.94, 126.86) | -7% |
| 90 | 92.75 ( 69.39, 119.50) | -8% |
| Average inter-decade difference* | | -7% |

*The average difference was calculated from the 6^th^ decade for gait speed, the 5^th^ decade for handgrip strength, and the 3^rd^ decade for the other outcomes. Note: No “between-group” comparisons could be performed as age was a continuous variable. The reader may consult Supplementary Tables 2, 3d and 3e to obtain the age-ranges in which significant differences were observed.

Supplementary Table 4b. Estimated marginal mean values of physical function and performance across the adult lifespan in men

| **Age** | **MEAN (95% CI)** | **Inter-decade difference (%)** |
| --- | --- | --- |
| **Gait speed (m/s)** | | |
| 20 | 1.27 (1.07, 1.46) |  |
| 30 | 1.31 (1.13, 1.48) | 3% |
| 40 | 1.33 (1.16, 1.50) | 2% |
| 50 | 1.34 (1.16, 1.51) | 1% |
| 60 | 1.32 (1.16, 1.49) | -1% |
| 70 | 1.3 (1.14, 1.46) | -2% |
| 80 | 1.25 (1.09, 1.41) | -4% |
| 90 | 1.19 (0.99, 1.38) | -5% |
| Average inter-decade difference* | | -3% |
| **Handgrip strength (kg)** | | |
| 20 | 45.98 (38.28, 54.38) |  |
| 30 | 47.58 (40.52, 55.21) | 3% |
| 40 | 48.15 (41.13, 55.72) | 1% |
| 50 | 47.66 (40.64, 55.23) | -1% |
| 60 | 46.12 (39.37, 53.41) | -3% |
| 70 | 43.6 (37.26, 50.43) | -5% |
| 80 | 40.17 (33.96, 46.90) | -8% |
| 90 | 35.96 (29.01, 43.65) | -10% |
| Average inter-decade difference* | | -4% |
| **5-time chair rise test (s)** | | |
| 20 | 7.18 (5.61, 8.94) |  |
| 30 | 7.61 (6.11, 9.28) | 6% |
| 40 | 8.06 (6.61, 9.65) | 6% |
| 50 | 8.52 (7.11, 10.05) | 6% |
| 60 | 8.99 (7.58, 10.51) | 6% |
| 70 | 9.47 (8.04, 11.02) | 5% |
| 80 | 9.97 (8.46, 11.59) | 5% |
| 90 | 10.48 (8.86, 12.23) | 5% |
| Average inter-decade difference* | | 6% |
| **V̇O_2_max (ml/kg/min)** | | |
| 20 | 35.39 (29.05, 42.35) |  |
| 30 | 32.92 (27.18, 39.22) | -7% |
| 40 | 30.55 (25.27, 36.33) | -7% |
| 50 | 28.26 (23.32, 33.67) | -7% |
| 60 | 26.06 (21.35, 31.24) | -8% |
| 70 | 23.95 (19.36, 29.03) | -8% |
| 80 | 21.93 (17.38, 27.01) | -8% |
| 90 | 20 (15.42, 25.17) | -9% |
| Average inter-decade difference* | | -8% |
| **Isokinetic strength (N-m)** | | |
| 20 | 143.2 (84.93, 216.59) |  |
| 30 | 134.14 (81.24, 200.23) | -6% |
| 40 | 125.37 (77.06, 185.39) | -7% |
| 50 | 116.91 (72.33, 172.14) | -7% |
| 60 | 108.73 (67.01, 160.50) | -7% |
| 70 | 100.86 (61.14, 150.46) | -7% |
| 80 | 93.28 (54.80, 141.93) | -8% |
| 90 | 85.99 (48.14, 134.75) | -8% |
| Average inter-decade difference* | | -7% |

*The average difference was calculated from the 6^th^ decade for gait speed, the 5^th^ decade for handgrip strength, and the 3^rd^ decade for the other outcomes. Note: No “between-group” comparisons could be performed as age was a continuous variable. The reader may consult Supplementary Tables 2, 3d and 3e to obtain the age-ranges in which significant differences were observed.

Supplementary Table 4c. Estimated marginal mean values of physical function and performance across the adult lifespan in women

| **Age** | **MEAN (95% CI)** | **Inter-decade difference (%)** |
| --- | --- | --- |
| **Gait speed (m/s)** | | |
| 20 | 1.05 (0.94, 1.15) |  |
| 30 | 1.11 (1.00, 1.21) | 6% |
| 40 | 1.14 (1.03, 1.25) | 3% |
| 50 | 1.15 (1.04, 1.27) | 1% |
| 60 | 1.14 (1.03, 1.26) | -1% |
| 70 | 1.11 (0.99, 1.22) | -3% |
| 80 | 1.05 (0.93, 1.16) | -5% |
| 90 | 0.96 (0.83, 1.10) | -9% |
| Average inter-decade difference* | | -4% |
| **Handgrip strength (kg)** | | |
| 20 | 29.01 (26.10, 32.06) |  |
| 30 | 28.94 (26.15, 31.87) | 0% |
| 40 | 28.51 (25.59, 31.58) | -1% |
| 50 | 27.72 (24.72, 30.90) | -3% |
| 60 | 26.6 (23.66, 29.70) | -4% |
| 70 | 25.15 (22.37, 28.08) | -5% |
| 80 | 23.41 (20.71, 26.27) | -7% |
| 90 | 21.41 (18.45, 24.59) | -9% |
| Average inter-decade difference* | | -5% |
| **5-time chair rise test (s)** | | |
| 20 | 7.23 (5.90, 8.70) |  |
| 30 | 7.74 (6.41, 9.18) | 7% |
| 40 | 8.26 (6.93, 9.70) | 7% |
| 50 | 8.8 (7.46, 10.25) | 7% |
| 60 | 9.36 (7.99, 10.83) | 6% |
| 70 | 9.93 (8.51, 11.46) | 6% |
| 80 | 10.52 (9.04, 12.12) | 6% |
| 90 | 11.13 (9.56, 12.82) | 6% |
| Average inter-decade difference* | | 6% |
| **V̇O_2_max (ml/kg/min)** | | |
| 20 | 36 (29.46, 43.20) |  |
| 30 | 33.43 (27.53, 39.92) | -7% |
| 40 | 30.96 (25.56, 36.87) | -7% |
| 50 | 28.58 (23.57, 34.08) | -8% |
| 60 | 26.3 (21.55, 31.52) | -8% |
| 70 | 24.11 (19.51, 29.19) | -8% |
| 80 | 22.02 (17.47, 27.08) | -9% |
| 90 | 20.02 (15.47, 25.15) | -9% |
| Average inter-decade difference* | | -8% |
| **Isokinetic strength (N-m)** | | |
| 20 | 122.64 (80.18, 174.09) |  |
| 30 | 115.33 (76.41, 162.24) | -6% |
| 40 | 108.25 (72.12, 151.70) | -6% |
| 50 | 101.39 (67.28, 142.48) | -6% |
| 60 | 94.76 (61.91, 134.58) | -7% |
| 70 | 88.35 (56.10, 127.89) | -7% |
| 80 | 82.17 (50.00, 122.29) | -7% |
| 90 | 76.21 (43.76, 117.59) | -7% |
| Average inter-decade difference* | | -7% |

*The average difference was calculated from the 6^th^ decade for gait speed, the 5^th^ decade for handgrip strength, and the 3^rd^ decade for the other outcomes. Note: No “between-group” comparisons could be performed as age was a continuous variable. The reader may consult Supplementary Tables 2, 3d and 3e to obtain the age-ranges in which significant differences were observed.

Supplementary Table 5a. Estimated marginal mean values of physical function and performance across the adult lifespan according to the levels of MVPA

|  | **Low MVPA** | |  | **High MVPA** | |
| --- | --- | --- | --- | --- | --- |
| **Age** | **MEAN (95% CI)** | **Inter-decade difference (%)** |  | **MEAN (95% CI)** | **Inter-decade difference (%)** |
| **Gait speed (m/s)** | | | | | |
| 20 | 1.11 (0.98, 1.25) |  |  | 1.17 (1.07, 1.28) |  |
| 30 | 1.19 (1.09, 1.29) | 7% |  | 1.21 (1.11, 1.31) | 3% |
| 40 | 1.24 (1.13, 1.34) | 4% |  | 1.23 (1.13, 1.34) | 2% |
| 50 | 1.26 (1.15, 1.36) | 2% |  | 1.24 (1.14, 1.35) | 1% |
| 60 | 1.24 (1.14, 1.35) | -2% |  | 1.23 (1.13, 1.34) | -1% |
| 70 | 1.2 (1.10, 1.31) | -3% |  | 1.21 (1.11, 1.31) | -2% |
| 80 | 1.13 (1.03, 1.24) | -6% |  | 1.18 (1.08, 1.28) | -2% |
| 90 | 1.03 (0.92, 1.15) | -9% |  | 1.12 (1.01, 1.24) | -5% |
| Average inter-decade difference* | | -5% |  |  | -2% |
| **Handgrip strength (kg)** | | | | | |
| 20 | 35.24 (31.38, 39.33) |  |  | 37.2 (33.67, 40.91) |  |
| 30 | 35.74 (32.46, 39.18) | 1% |  | 37.06 (33.79, 40.47) | 0% |
| 40 | 35.69 (32.31, 39.24) | 0% |  | 36.45 (33.17, 39.88) | -2% |
| 50 | 35.09 (31.64, 38.72) | -2% |  | 35.4 (32.10, 38.86) | -3% |
| 60 | 33.96 (30.60, 37.49) | -3% |  | 33.92 (30.72, 37.27) | -4% |
| 70 | 32.32 (29.18, 35.61) | -5% |  | 32.04 (29.03, 35.20) | -6% |
| 80 | 30.21 (27.23, 33.34) | -7% |  | 29.8 (26.88, 32.87) | -7% |
| 90 | 27.68 (24.46, 31.11) | -8% |  | 27.25 (24.06, 30.63) | -9% |
| Average inter-decade difference* | | -4% |  |  | -5% |
| **5-time chair rise test (s)** | | | | | |
| 20 | 7.42 ( 6.34, 8.59) |  |  | 7.67 (6.63, 8.78) |  |
| 30 | 7.96 ( 6.91, 9.08) | 7% |  | 8.08 (7.07, 9.15) | 5% |
| 40 | 8.51 ( 7.50, 9.59) | 7% |  | 8.5 (7.52, 9.55) | 5% |
| 50 | 9.09 ( 8.09, 10.15) | 7% |  | 8.94 (7.96, 9.97) | 5% |
| 60 | 9.69 ( 8.69, 10.74) | 7% |  | 9.38 (8.39, 10.43) | 5% |
| 70 | 10.3 ( 9.27, 11.38) | 6% |  | 9.84 (8.82, 10.92) | 5% |
| 80 | 10.93 ( 9.86, 12.06) | 6% |  | 10.3 (9.23, 11.44) | 5% |
| 90 | 11.58 (10.43, 12.80) | 6% |  | 10.78 (9.63, 12.00) | 5% |
| Average inter-decade difference* | | 7% |  |  | 5% |
| **V̇O_2_max (ml/kg/min)** | | | | | |
| 20 | 28.16 (22.54, 34.41) |  |  | 38.74 (32.35, 45.70) |  |
| 30 | 26.94 (21.92, 32.47) | -4% |  | 35.47 (29.81, 41.62) | -8% |
| 40 | 25.74 (21.21, 30.70) | -4% |  | 32.34 (27.27, 37.86) | -9% |
| 50 | 24.57 (20.39, 29.14) | -5% |  | 29.36 (24.72, 34.41) | -9% |
| 60 | 23.43 (19.43, 27.79) | -5% |  | 26.53 (22.17, 31.28) | -10% |
| 70 | 22.31 (18.35, 26.66) | -5% |  | 23.84 (19.64, 28.44) | -10% |
| 80 | 21.22 (17.16, 25.72) | -5% |  | 21.29 (17.15, 25.88) | -11% |
| 90 | 20.16 (15.89, 24.94) | -5% |  | 18.88 (14.74, 23.54) | -11% |
| Average inter-decade difference* | | -5% |  |  | -10% |
| **Isokinetic strength (N-m)** | | | | | |
| 20 | 153.95 (115.23, 198.26) |  |  | 149.91 (114.28, 190.37) |  |
| 30 | 143.75 (109.76, 182.32) | -7% |  | 141.57 (109.49, 177.78) | -6% |
| 40 | 133.9 (103.87, 167.74) | -7% |  | 133.47 (104.29, 166.25) | -6% |
| 50 | 124.4 ( 97.44, 154.65) | -7% |  | 125.61 ( 98.63, 155.86) | -6% |
| 60 | 115.25 ( 90.39, 143.13) | -7% |  | 117.99 ( 92.45, 146.64) | -6% |
| 70 | 106.45 ( 82.70, 133.20) | -8% |  | 110.6 ( 85.78, 138.57) | -6% |
| 80 | 98 ( 74.48, 124.75) | -8% |  | 103.46 ( 78.72, 131.57) | -6% |
| 90 | 89.9 ( 65.96, 117.55) | -8% |  | 96.55 ( 71.39, 125.49) | -7% |
| Average inter-decade difference* | | -7% |  |  | -6% |

*The average difference was calculated from the 6^th^ decade for gait speed, the 5^th^ decade for handgrip strength, and the 3^rd^ decade for the other outcomes. Low and high MVPA levels were defined using the first and third quartile values of the distribution, which roughly equal to 10 min/d and 37 min/d, respectively. Note: No “between-group” comparisons could be performed as age and PA were continuous variables. The reader may consult Supplementary Tables 2, 3d and 3e to obtain the age-ranges in which significant differences were observed.

Supplementary Table 5b. Estimated marginal mean values of physical function and performance across the adult lifespan according to the levels of MVPA in men

|  | **Low MVPA** | |  | **High MVPA** | |
| --- | --- | --- | --- | --- | --- |
| **Age** | **MEAN (95% CI)** | **Inter-decade difference (%)** |  | **MEAN (95% CI)** | **Inter-decade difference (%)** |
| **Gait speed (m/s)** | | | | | |
| 20 | 1.24 (1.01, 1.48) |  |  | 1.31 (1.10, 1.51) |  |
| 30 | 1.3 (1.12, 1.49) | 5% |  | 1.31 (1.14, 1.49) | 0% |
| 40 | 1.34 (1.16, 1.51) | 3% |  | 1.32 (1.15, 1.48) | 1% |
| 50 | 1.35 (1.17, 1.52) | 1% |  | 1.32 (1.15, 1.49) | 0% |
| 60 | 1.33 (1.16, 1.50) | -1% |  | 1.31 (1.15, 1.48) | -1% |
| 70 | 1.29 (1.13, 1.45) | -3% |  | 1.31 (1.15, 1.47) | 0% |
| 80 | 1.22 (1.05, 1.39) | -5% |  | 1.3 (1.13, 1.47) | -1% |
| 90 | 1.13 (0.92, 1.34) | -7% |  | 1.29 (1.09, 1.49) | -1% |
| Average inter-decade difference* | | -4% |  |  | -1% |
| **Handgrip strength (kg)** | | | | | |
| 20 | 46.2 (37.71, 55.55) |  |  | 45.59 (37.52, 54.46) |  |
| 30 | 48 (40.48, 56.17) | 4% |  | 46.83 (39.81, 54.43) | 3% |
| 40 | 48.67 (41.36, 56.57) | 1% |  | 47.22 (40.37, 54.62) | 1% |
| 50 | 48.16 (40.92, 55.99) | -1% |  | 46.75 (39.86, 54.19) | -1% |
| 60 | 46.51 (39.58, 53.99) | -3% |  | 45.43 (38.71, 52.69) | -3% |
| 70 | 43.76 (37.33, 50.70) | -6% |  | 43.3 (36.91, 50.20) | -5% |
| 80 | 40.03 (33.72, 46.88) | -9% |  | 40.42 (34.07, 47.31) | -7% |
| 90 | 35.46 (28.23, 43.51) | -11% |  | 36.87 (29.64, 44.89) | -9% |
| Average inter-decade difference* | | -5% |  |  | -4% |
| **5-time chair rise test (s)** | | | | | |
| 20 | 6.89 (5.26, 8.74) |  |  | 7.72 (6.12, 9.50) |  |
| 30 | 7.4 (5.85, 9.14) | 7% |  | 7.99 (6.48, 9.66) | 3% |
| 40 | 7.94 (6.46, 9.57) | 7% |  | 8.27 (6.82, 9.86) | 4% |
| 50 | 8.5 (7.06, 10.06) | 7% |  | 8.55 (7.14, 10.09) | 3% |
| 60 | 9.07 (7.65, 10.61) | 7% |  | 8.84 (7.42, 10.38) | 3% |
| 70 | 9.66 (8.22, 11.22) | 7% |  | 9.13 (7.67, 10.72) | 3% |
| 80 | 10.27 (8.75, 11.92) | 6% |  | 9.43 (7.89, 11.11) | 3% |
| 90 | 10.9 (9.25, 12.69) | 6% |  | 9.73 (8.06, 11.55) | 3% |
| Average inter-decade difference* | | 7% |  |  | 3% |
| **V̇O_2_max (ml/kg/min)** | | | | | |
| 20 | 30.47 (23.94, 37.78) |  |  | 42.26 (34.92, 50.29) |  |
| 30 | 28.94 (23.14, 35.38) | -5% |  | 38.43 (31.93, 45.52) | -9% |
| 40 | 27.45 (22.24, 33.20) | -5% |  | 34.78 (28.95, 41.14) | -9% |
| 50 | 25.99 (21.19, 31.28) | -5% |  | 31.31 (25.97, 37.15) | -10% |
| 60 | 24.58 (20.00, 29.63) | -5% |  | 28.03 (23.01, 33.53) | -10% |
| 70 | 23.21 (18.66, 28.25) | -6% |  | 24.92 (20.10, 30.27) | -11% |
| 80 | 21.87 (17.20, 27.10) | -6% |  | 22 (17.26, 27.32) | -12% |
| 90 | 20.58 (15.68, 26.14) | -6% |  | 19.26 (14.54, 24.65) | -12% |
| Average inter-decade difference* | | -5% |  |  | -11% |
| **Isokinetic strength (N-m)** | | | | | |
| 20 | 150.15 (85.49, 232.89) |  |  | 134.5 (76.36, 209.00) |  |
| 30 | 139.26 (82.19, 211.29) | -7% |  | 127.69 (74.87, 194.53) | -5% |
| 40 | 128.78 (78.27, 191.79) | -8% |  | 121.06 (72.55, 181.93) | -5% |
| 50 | 118.71 (73.61, 174.54) | -8% |  | 114.6 (69.25, 171.33) | -5% |
| 60 | 109.05 (68.07, 159.65) | -8% |  | 108.32 (64.92, 162.78) | -5% |
| 70 | 99.8 (61.60, 147.18) | -8% |  | 102.22 (59.63, 156.22) | -6% |
| 80 | 90.96 (54.30, 137.03) | -9% |  | 96.3 (53.57, 151.47) | -6% |
| 90 | 82.53 (46.44, 128.93) | -9% |  | 90.55 (47.01, 148.24) | -6% |
| Average inter-decade difference* | | -8% |  |  | -5% |

*The average difference was calculated from the 6^th^ decade for gait speed, the 5^th^ decade for handgrip strength, and the 3^rd^ decade for the other outcomes. Low and high MVPA levels were defined using the first and third quartile values of the distribution, which roughly equal to 11 min/d and 39 min/d, respectively. Note: No “between-group” comparisons could be performed as age and PA were continuous variables. The reader may consult Supplementary Tables 2, 3d and 3e to obtain the age-ranges in which significant differences were observed.

Supplementary Table 5c. Estimated marginal mean values of physical function and performance across the adult lifespan according to the levels of MVPA in women

|  | **Low MVPA** | |  | **High MVPA** | |
| --- | --- | --- | --- | --- | --- |
| **Age** | **MEAN (95% CI)** | **Inter-decade difference (%)** |  | **MEAN (95% CI)** | **Inter-decade difference (%)** |
| **Gait speed (m/s)** | | | | | |
| 20 | 1.03 (0.85, 1.21) |  |  | 1.07 (0.94, 1.19) |  |
| 30 | 1.1 (0.99, 1.21) | 7% |  | 1.12 (1.01, 1.23) | 5% |
| 40 | 1.14 (1.02, 1.25) | 4% |  | 1.15 (1.03, 1.26) | 3% |
| 50 | 1.15 (1.03, 1.27) | 1% |  | 1.15 (1.04, 1.27) | 0% |
| 60 | 1.14 (1.02, 1.26) | -1% |  | 1.14 (1.03, 1.25) | -1% |
| 70 | 1.11 (0.99, 1.22) | -3% |  | 1.11 (0.99, 1.22) | -3% |
| 80 | 1.04 (0.93, 1.16) | -6% |  | 1.05 (0.93, 1.17) | -5% |
| 90 | 0.96 (0.82, 1.10) | -8% |  | 0.97 (0.83, 1.12) | -8% |
| Average inter-decade difference* | | -4% |  |  | -4% |
| **Handgrip strength (kg)** | | | | | |
| 20 | 28.16 (23.94, 32.72) |  |  | 30.18 (26.86, 33.69) |  |
| 30 | 28.25 (25.44, 31.21) | 0% |  | 29.89 (26.91, 33.03) | -1% |
| 40 | 28 (25.04, 31.12) | -1% |  | 29.22 (26.24, 32.36) | -2% |
| 50 | 27.4 (24.32, 30.67) | -2% |  | 28.17 (25.18, 31.33) | -4% |
| 60 | 26.47 (23.43, 29.70) | -3% |  | 26.76 (23.88, 29.80) | -5% |
| 70 | 25.23 (22.35, 28.29) | -5% |  | 25.03 (22.32, 27.90) | -6% |
| 80 | 23.7 (20.88, 26.71) | -6% |  | 23.01 (20.28, 25.91) | -8% |
| 90 | 21.91 (18.71, 25.37) | -8% |  | 20.74 (17.55, 24.19) | -10% |
| Average inter-decade difference* | | -4% |  |  | -6% |
| **5-time chair rise test (s)** | | | | | |
| 20 | 7.15 (5.77, 8.68) |  |  | 7.33 (5.98, 8.82) |  |
| 30 | 7.7 (6.34, 9.18) | 8% |  | 7.79 (6.46, 9.25) | 6% |
| 40 | 8.26 (6.91, 9.72) | 7% |  | 8.26 (6.93, 9.71) | 6% |
| 50 | 8.84 (7.49, 10.30) | 7% |  | 8.74 (7.40, 10.20) | 6% |
| 60 | 9.44 (8.06, 10.93) | 7% |  | 9.24 (7.87, 10.72) | 6% |
| 70 | 10.06 (8.63, 11.60) | 7% |  | 9.75 (8.33, 11.29) | 6% |
| 80 | 10.7 (9.19, 12.33) | 6% |  | 10.28 (8.78, 11.89) | 5% |
| 90 | 11.36 (9.74, 13.11) | 6% |  | 10.82 (9.22, 12.54) | 5% |
| Average inter-decade difference* | | 7% |  |  | 6% |
| **V̇O_2_max (ml/kg/min)** | | | | | |
| 20 | 31.88 (25.26, 39.28) |  |  | 43.12 (35.45, 51.53) |  |
| 30 | 30.1 (24.20, 36.64) | -6% |  | 39.14 (32.40, 46.51) | -9% |
| 40 | 28.36 (23.05, 34.23) | -6% |  | 35.35 (29.36, 41.90) | -10% |
| 50 | 26.68 (21.79, 32.06) | -6% |  | 31.76 (26.33, 37.69) | -10% |
| 60 | 25.05 (20.42, 30.16) | -6% |  | 28.35 (23.31, 33.90) | -11% |
| 70 | 23.47 (18.93, 28.50) | -6% |  | 25.14 (20.32, 30.48) | -11% |
| 80 | 21.94 (17.36, 27.07) | -7% |  | 22.13 (17.41, 27.41) | -12% |
| 90 | 20.47 (15.73, 25.82) | -7% |  | 19.3 (14.62, 24.64) | -13% |
| Average inter-decade difference* | | -6% |  |  | -11% |
| **Isokinetic strength (N-m)** | | | | | |
| 20 | 119.41 (74.35, 175.09) |  |  | 127.9 (83.22, 182.15) |  |
| 30 | 113.13 (72.67, 162.50) | -5% |  | 118.91 (78.43, 167.77) | -7% |
| 40 | 107.02 (70.14, 151.65) | -5% |  | 110.24 (73.18, 154.87) | -7% |
| 50 | 101.08 (66.61, 142.70) | -6% |  | 101.9 (67.42, 143.49) | -8% |
| 60 | 95.3 (62.03, 135.70) | -6% |  | 93.89 (61.18, 133.59) | -8% |
| 70 | 89.7 (56.52, 130.51) | -6% |  | 86.21 (54.55, 125.09) | -8% |
| 80 | 84.27 (50.33, 126.90) | -6% |  | 78.86 (47.68, 117.83) | -9% |
| 90 | 79.01 (43.79, 124.55) | -6% |  | 71.83 (40.77, 111.63) | -9% |
| Average inter-decade difference* | | -6% |  |  | -8% |

*The average difference was calculated from the 6^th^ decade for gait speed, the 5^th^ decade for handgrip strength, and the 3^rd^ decade for the other outcomes. Low and high MVPA levels were defined using the first and third quartile values of the distribution, which roughly equal to 10 min/d and 36 min/d, respectively. Note: No “between-group” comparisons could be performed as age and PA were continuous variables. The reader may consult Supplementary Tables 2, 3d and 3e to obtain the age-ranges in which significant differences were observed.

Supplementary Table 6a. Estimated marginal mean values of physical function and performance across the adult lifespan according to the levels of SB

|  | **High SB** | |  | **Low SB** | |
| --- | --- | --- | --- | --- | --- |
| **Age** | **MEAN (95% CI)** | **Inter-decade difference (%)** |  | **MEAN (95% CI)** | **Inter-decade difference (%)** |
| **Gait speed (m/s)** | | | | | |
| 20 | 1.15 (1.05, 1.26) |  |  | 1.12 (0.99, 1.26) |  |
| 30 | 1.2 (1.10, 1.30) | 4% |  | 1.2 (1.10, 1.30) | 7% |
| 40 | 1.22 (1.12, 1.33) | 2% |  | 1.25 (1.14, 1.35) | 4% |
| 50 | 1.23 (1.12, 1.34) | 1% |  | 1.27 (1.16, 1.37) | 2% |
| 60 | 1.22 (1.11, 1.33) | -1% |  | 1.26 (1.15, 1.36) | -1% |
| 70 | 1.19 (1.09, 1.29) | -2% |  | 1.22 (1.12, 1.32) | -3% |
| 80 | 1.14 (1.04, 1.24) | -4% |  | 1.16 (1.06, 1.26) | -5% |
| 90 | 1.08 (0.96, 1.19) | -5% |  | 1.07 (0.95, 1.18) | -8% |
| Average inter-decade difference* | | -3% |  |  | -4% |
| **Handgrip strength (kg)** | | | | | |
| 20 | 34.95 (31.69, 38.37) |  |  | 37.07 (33.10, 41.28) |  |
| 30 | 35.66 (32.50, 38.97) | 2% |  | 36.86 (33.51, 40.36) | -1% |
| 40 | 35.74 (32.44, 39.19) | 0% |  | 36.25 (32.86, 39.80) | -2% |
| 50 | 35.17 (31.79, 38.73) | -2% |  | 35.26 (31.83, 38.85) | -3% |
| 60 | 33.99 (30.68, 37.47) | -3% |  | 33.9 (30.60, 37.37) | -4% |
| 70 | 32.21 (29.10, 35.48) | -5% |  | 32.2 (29.14, 35.41) | -5% |
| 80 | 29.89 (26.93, 33.01) | -7% |  | 30.18 (27.26, 33.26) | -6% |
| 90 | 27.1 (23.97, 30.42) | -9% |  | 27.89 (24.63, 31.35) | -8% |
| Average inter-decade difference* | | -4% |  |  | -5% |
| **5-time chair rise test (s)** | | | | | |
| 20 | 7.25 ( 6.23, 8.35) |  |  | 7.78 (6.68, 8.96) |  |
| 30 | 7.79 ( 6.79, 8.85) | 7% |  | 8.22 (7.16, 9.35) | 6% |
| 40 | 8.34 ( 7.36, 9.39) | 7% |  | 8.67 (7.65, 9.75) | 5% |
| 50 | 8.92 ( 7.94, 9.96) | 7% |  | 9.13 (8.13, 10.19) | 5% |
| 60 | 9.51 ( 8.52, 10.56) | 7% |  | 9.61 (8.61, 10.66) | 5% |
| 70 | 10.13 ( 9.10, 11.22) | 7% |  | 10.09 (9.08, 11.16) | 5% |
| 80 | 10.76 ( 9.67, 11.91) | 6% |  | 10.59 (9.53, 11.70) | 5% |
| 90 | 11.42 (10.24, 12.65) | 6% |  | 11.1 (9.98, 12.28) | 5% |
| Average inter-decade difference* | | 7% |  |  | 5% |
| **V̇O_2_max (ml/kg/min)** | | | | | |
| 20 | 32.57 (27.05, 38.61) |  |  | 32.51 (26.58, 39.04) |  |
| 30 | 30.5 (25.51, 35.94) | -6% |  | 30.48 (25.18, 36.30) | -6% |
| 40 | 28.5 (23.92, 33.49) | -7% |  | 28.52 (23.72, 33.76) | -6% |
| 50 | 26.57 (22.27, 31.24) | -7% |  | 26.62 (22.21, 31.43) | -7% |
| 60 | 24.7 (20.57, 29.20) | -7% |  | 24.79 (20.63, 29.32) | -7% |
| 70 | 22.9 (18.84, 27.36) | -7% |  | 23.02 (19.00, 27.43) | -7% |
| 80 | 21.17 (17.08, 25.70) | -8% |  | 21.32 (17.32, 25.73) | -7% |
| 90 | 19.51 (15.33, 24.18) | -8% |  | 19.68 (15.63, 24.20) | -8% |
| Average inter-decade difference* | | -7% |  |  | -7% |
| **Isokinetic strength (N-m)** | | | | | |
| 20 | 163.8 (127.12, 205.12) |  |  | 142.92 (107.04, 183.97) |  |
| 30 | 151.29 (118.52, 188.05) | -8% |  | 135.97 (103.74, 172.55) | -5% |
| 40 | 139.28 (109.74, 172.33) | -8% |  | 129.2 (100.06, 162.06) | -5% |
| 50 | 127.76 (100.74, 158.00) | -8% |  | 122.6 ( 95.89, 152.59) | -5% |
| 60 | 116.75 ( 91.50, 145.07) | -9% |  | 116.18 ( 91.17, 144.20) | -5% |
| 70 | 106.23 ( 82.09, 133.47) | -9% |  | 109.92 ( 85.87, 136.94) | -5% |
| 80 | 96.2 ( 72.61, 123.11) | -9% |  | 103.84 ( 80.04, 130.75) | -6% |
| 90 | 86.67 ( 63.22, 113.82) | -10% |  | 97.94 ( 73.78, 125.52) | -6% |
| Average inter-decade difference* | | -9% |  |  | -5% |

*The average difference was calculated from the 6^th^ decade for gait speed, the 5^th^ decade for handgrip strength, and the 3^rd^ decade for the other outcomes. Low and high SB levels were defined using the first and third quartile values of the distribution, which roughly equal to 5.4h/d and 7.7h/d, respectively. Note: No “between-group” comparisons could be performed as age and SB were continuous variables. The reader may consult Supplementary Tables 2, 3d and 3e to obtain the age-ranges in which significant differences were observed.

Supplementary Table 6b. Estimated marginal mean values of physical function and performance across the adult lifespan according to the levels of SB in men

|  | **High SB** | |  | **Low SB** | |
| --- | --- | --- | --- | --- | --- |
| **Age** | **MEAN (95% CI)** | **Inter-decade difference (%)** |  | **MEAN (95% CI)** | **Inter-decade difference (%)** |
| **Gait speed (m/s)** | | | | | |
| 20 | 1.3 (1.10, 1.49) |  |  | 1.24 (1.00, 1.47) |  |
| 30 | 1.32 (1.15, 1.50) | 2% |  | 1.29 (1.11, 1.47) | 4% |
| 40 | 1.33 (1.16, 1.51) | 1% |  | 1.33 (1.15, 1.50) | 3% |
| 50 | 1.33 (1.16, 1.50) | 0% |  | 1.34 (1.16, 1.52) | 1% |
| 60 | 1.31 (1.14, 1.48) | -2% |  | 1.34 (1.16, 1.51) | 0% |
| 70 | 1.28 (1.11, 1.44) | -2% |  | 1.31 (1.15, 1.47) | -2% |
| 80 | 1.23 (1.06, 1.40) | -4% |  | 1.27 (1.10, 1.43) | -3% |
| 90 | 1.17 (0.97, 1.38) | -5% |  | 1.2 (0.98, 1.42) | -6% |
| Average inter-decade difference* | | -3% |  |  | -3% |
| **Handgrip strength (kg)** | | | | | |
| 20 | 45.44 (37.57, 54.06) |  |  | 46.49 (37.98, 55.88) |  |
| 30 | 46.95 (39.86, 54.62) | 3% |  | 48.19 (40.71, 56.29) | 4% |
| 40 | 47.5 (40.44, 55.13) | 1% |  | 48.76 (41.37, 56.76) | 1% |
| 50 | 47.08 (39.97, 54.77) | -1% |  | 48.2 (40.78, 56.24) | -1% |
| 60 | 45.7 (38.81, 53.16) | -3% |  | 46.52 (39.45, 54.17) | -3% |
| 70 | 43.4 (36.92, 50.41) | -5% |  | 43.78 (37.33, 50.74) | -6% |
| 80 | 40.26 (33.89, 47.17) | -7% |  | 40.09 (33.77, 46.94) | -8% |
| 90 | 36.37 (29.20, 44.33) | -10% |  | 35.57 (28.04, 44.01) | -11% |
| Average inter-decade difference* | | -4% |  |  | -5% |
| **5-time chair rise test (s)** | | | | | |
| 20 | 6.59 (5.06, 8.32) |  |  | 7.76 (6.03, 9.70) |  |
| 30 | 7.12 (5.65, 8.75) | 8% |  | 8.09 (6.47, 9.90) | 4% |
| 40 | 7.66 (6.25, 9.22) | 8% |  | 8.44 (6.90, 10.13) | 4% |
| 50 | 8.23 (6.84, 9.75) | 7% |  | 8.79 (7.32, 10.40) | 4% |
| 60 | 8.82 (7.42, 10.34) | 7% |  | 9.15 (7.71, 10.71) | 4% |
| 70 | 9.43 (7.97, 11.00) | 7% |  | 9.51 (8.06, 11.09) | 4% |
| 80 | 10.05 (8.49, 11.75) | 7% |  | 9.89 (8.38, 11.52) | 4% |
| 90 | 10.7 (8.98, 12.57) | 6% |  | 10.27 (8.65, 12.02) | 4% |
| Average inter-decade difference* | | 7% |  |  | 4% |
| **V̇O_2_max (ml/kg/min)** | | | | | |
| 20 | 35.94 (29.36, 43.18) |  |  | 35.01 (28.30, 42.44) |  |
| 30 | 33.33 (27.44, 39.79) | -7% |  | 32.64 (26.63, 39.28) | -7% |
| 40 | 30.82 (25.46, 36.69) | -8% |  | 30.36 (24.91, 36.35) | -7% |
| 50 | 28.41 (23.42, 33.87) | -8% |  | 28.16 (23.13, 33.68) | -7% |
| 60 | 26.09 (21.32, 31.35) | -8% |  | 26.04 (21.29, 31.26) | -8% |
| 70 | 23.88 (19.18, 29.08) | -8% |  | 24 (19.41, 29.08) | -8% |
| 80 | 21.76 (17.04, 27.05) | -9% |  | 22.05 (17.50, 27.12) | -8% |
| 90 | 19.74 (14.93, 25.22) | -9% |  | 20.18 (15.59, 25.36) | -8% |
| Average inter-decade difference* | | -8% |  |  | -8% |
| **Isokinetic strength (N-m)** | | | | | |
| 20 | 152.4 (88.43, 233.68) |  |  | 136.96 (79.18, 210.49) |  |
| 30 | 141.57 (84.53, 213.22) | -7% |  | 129.09 (76.65, 195.12) | -6% |
| 40 | 131.13 (80.05, 194.74) | -7% |  | 121.45 (73.51, 181.36) | -6% |
| 50 | 121.09 (74.88, 178.35) | -8% |  | 114.04 (69.66, 169.30) | -6% |
| 60 | 111.45 (68.95, 164.11) | -8% |  | 106.86 (65.06, 158.98) | -6% |
| 70 | 102.21 (62.25, 152.04) | -8% |  | 99.92 (59.73, 150.40) | -6% |
| 80 | 93.37 (54.90, 142.00) | -9% |  | 93.21 (53.78, 143.43) | -7% |
| 90 | 84.93 (47.13, 133.78) | -9% |  | 86.74 (47.39, 137.88) | -7% |
| Average inter-decade difference* | | -8% |  |  | -6% |

*The average difference was calculated from the 6^th^ decade for gait speed, the 5^th^ decade for handgrip strength, and the 3^rd^ decade for the other outcomes. Low and high SB levels were defined using the first and third quartile values of the distribution, which roughly equal to 5.6h/d and 8.4h/d, respectively. Note: No “between-group” comparisons could be performed as age and SB were continuous variables. The reader may consult Supplementary Tables 2, 3d and 3e to obtain the age-ranges in which significant differences were observed.

Supplementary Table 6c. Estimated marginal mean values of physical function and performance across the adult lifespan according to the levels of SB in women

|  | **High SB** | |  | **Low SB** | |
| --- | --- | --- | --- | --- | --- |
| **Age** | **MEAN (95% CI)** | **Inter-decade difference (%)** |  | **MEAN (95% CI)** | **Inter-decade difference (%)** |
| **Gait speed (m/s)** | | | | | |
| 20 | 1.04 (0.92, 1.16) |  |  | 1.05 (0.87, 1.23) |  |
| 30 | 1.09 (0.98, 1.20) | 5% |  | 1.12 (1.01, 1.23) | 7% |
| 40 | 1.12 (1.01, 1.23) | 3% |  | 1.16 (1.04, 1.27) | 4% |
| 50 | 1.13 (1.02, 1.25) | 1% |  | 1.17 (1.05, 1.29) | 1% |
| 60 | 1.12 (1.01, 1.24) | -1% |  | 1.16 (1.04, 1.28) | -1% |
| 70 | 1.09 (0.98, 1.21) | -3% |  | 1.12 (1.01, 1.23) | -3% |
| 80 | 1.04 (0.92, 1.16) | -5% |  | 1.05 (0.94, 1.17) | -6% |
| 90 | 0.97 (0.83, 1.11) | -7% |  | 0.96 (0.82, 1.09) | -9% |
| Average inter-decade difference* | | -4% |  |  | -5% |
| **Handgrip strength (kg)** | | | | | |
| 20 | 27.87 (24.85, 31.07) |  |  | 30.08 (25.73, 34.77) |  |
| 30 | 28.3 (25.49, 31.26) | 2% |  | 29.54 (26.62, 32.62) | -2% |
| 40 | 28.24 (25.33, 31.31) | 0% |  | 28.77 (25.76, 31.93) | -3% |
| 50 | 27.69 (24.69, 30.87) | -2% |  | 27.75 (24.67, 31.01) | -4% |
| 60 | 26.68 (23.72, 29.82) | -4% |  | 26.52 (23.52, 29.69) | -4% |
| 70 | 25.22 (22.39, 28.22) | -5% |  | 25.08 (22.29, 28.03) | -5% |
| 80 | 23.36 (20.55, 26.34) | -7% |  | 23.45 (20.75, 26.32) | -6% |
| 90 | 21.14 (17.99, 24.54) | -10% |  | 21.67 (18.58, 24.99) | -8% |
| Average inter-decade difference* | | -5% |  |  | -5% |
| **5-time chair rise test (s)** | | | | | |
| 20 | 7.16 (5.83, 8.63) |  |  | 7.29 (5.91, 8.83) |  |
| 30 | 7.69 (6.37, 9.14) | 7% |  | 7.78 (6.42, 9.27) | 7% |
| 40 | 8.24 (6.91, 9.68) | 7% |  | 8.28 (6.93, 9.74) | 6% |
| 50 | 8.8 (7.45, 10.27) | 7% |  | 8.79 (7.45, 10.25) | 6% |
| 60 | 9.39 (8.00, 10.90) | 7% |  | 9.32 (7.96, 10.79) | 6% |
| 70 | 10 (8.54, 11.57) | 6% |  | 9.87 (8.47, 11.38) | 6% |
| 80 | 10.62 (9.07, 12.29) | 6% |  | 10.43 (8.97, 12.01) | 6% |
| 90 | 11.26 (9.61, 13.05) | 6% |  | 11.01 (9.45, 12.68) | 6% |
| Average inter-decade difference* | | 7% |  |  | 6% |
| **V̇O_2_max (ml/kg/min)** | | | | | |
| 20 | 36.27 (29.89, 43.28) |  |  | 35.83 (28.75, 43.68) |  |
| 30 | 33.62 (27.83, 39.94) | -7% |  | 33.32 (27.03, 40.26) | -7% |
| 40 | 31.06 (25.75, 36.86) | -8% |  | 30.9 (25.26, 37.10) | -7% |
| 50 | 28.6 (23.64, 34.04) | -8% |  | 28.57 (23.42, 34.23) | -8% |
| 60 | 26.25 (21.50, 31.47) | -8% |  | 26.33 (21.52, 31.63) | -8% |
| 70 | 23.99 (19.35, 29.14) | -9% |  | 24.19 (19.55, 29.31) | -8% |
| 80 | 21.84 (17.21, 27.01) | -9% |  | 22.13 (17.55, 27.24) | -9% |
| 90 | 19.79 (15.13, 25.07) | -9% |  | 20.17 (15.54, 25.39) | -9% |
| Average inter-decade difference* | | -8% |  |  | -8% |
| **Isokinetic strength (N-m)** | | | | | |
| 20 | 135.32 (91.91, 187.09) |  |  | 114.63 (71.07, 168.54) |  |
| 30 | 124.54 (84.91, 171.74) | -8% |  | 109.46 (69.83, 157.96) | -5% |
| 40 | 114.21 (77.42, 158.12) | -8% |  | 104.41 (67.97, 148.65) | -5% |
| 50 | 104.33 (69.51, 146.19) | -9% |  | 99.48 (65.39, 140.70) | -5% |
| 60 | 94.89 (61.27, 135.83) | -9% |  | 94.67 (62.04, 134.18) | -5% |
| 70 | 85.9 (52.90, 126.86) | -9% |  | 89.98 (57.94, 129.05) | -5% |
| 80 | 77.36 (44.62, 119.06) | -10% |  | 85.41 (53.20, 125.22) | -5% |
| 90 | 69.27 (36.63, 112.21) | -10% |  | 80.96 (48.00, 122.49) | -5% |
| Average inter-decade difference* | | -9% |  |  | -5% |

*The average difference was calculated from the 6^th^ decade for gait speed, the 5^th^ decade for handgrip strength, and the 3^rd^ decade for the other outcomes. Low and high SB levels were defined using the first and third quartile values of the distribution, which roughly equal to 5.3h/d and 7.5h/d, respectively. Note: No “between-group” comparisons could be performed as age and SB were continuous variables. The reader may consult Supplementary Tables 2, 3d and 3e to obtain the age-ranges in which significant differences were observed.

Supplementary Table 7a. Estimated marginal mean values of physical function and performance across the adult lifespan according to the levels of MVPA and SB (1/2)

|  | **Low MVPA / High SB** | |  | **Low MVPA / Low SB** | |  | **High MVPA / High SB** | |  | **High MVPA / Low SB** | |
| --- | --- | --- | --- | --- | --- | --- | --- | --- | --- | --- | --- |
| **Age** | **MEAN (95% CI)** | **Inter-decade difference (%)** |  | **MEAN (95% CI)** | **Inter-decade difference (%)** |  | **MEAN (95% CI)** | **Inter-decade difference (%)** |  | **MEAN (95% CI)** | **Inter-decade difference (%)** |
| **Gait speed (m/s)** | | | | | | | | | | | |
| 20 | 1.12 (0.99, 1.26) |  |  | 1.1 (0.95, 1.25) |  |  | 1.19 (1.08, 1.31) |  |  | 1.16 (1.03, 1.28) |  |
| 30 | 1.18 (1.08, 1.29) | 5% |  | 1.2 (1.07, 1.32) | 9% |  | 1.22 (1.12, 1.32) | 3% |  | 1.2 (1.10, 1.31) | 3% |
| 40 | 1.22 (1.11, 1.32) | 3% |  | 1.26 (1.15, 1.36) | 5% |  | 1.23 (1.13, 1.33) | 1% |  | 1.24 (1.13, 1.34) | 3% |
| 50 | 1.23 (1.12, 1.34) | 1% |  | 1.28 (1.17, 1.39) | 2% |  | 1.23 (1.13, 1.34) | 0% |  | 1.25 (1.14, 1.36) | 1% |
| 60 | 1.22 (1.11, 1.33) | -1% |  | 1.27 (1.16, 1.38) | -1% |  | 1.22 (1.12, 1.33) | -1% |  | 1.25 (1.14, 1.35) | 0% |
| 70 | 1.19 (1.08, 1.29) | -2% |  | 1.22 (1.12, 1.32) | -4% |  | 1.2 (1.10, 1.30) | -2% |  | 1.23 (1.13, 1.32) | -2% |
| 80 | 1.13 (1.03, 1.23) | -5% |  | 1.14 (1.04, 1.24) | -7% |  | 1.16 (1.06, 1.27) | -3% |  | 1.19 (1.09, 1.29) | -3% |
| 90 | 1.05 (0.93, 1.17) | -7% |  | 1.02 (0.90, 1.14) | -11% |  | 1.12 (0.99, 1.24) | -3% |  | 1.13 (1.01, 1.25) | -5% |
| Average inter-decade difference* | | -4% |  |  | -5% |  |  | -2% |  |  | -2% |
| **Handgrip strength (kg)** | | | | | | | | | | | |
| 20 | 34.82 (30.90, 38.97) |  |  | 35.64 (31.36, 40.20) |  |  | 35.14 (31.55, 38.92) |  |  | 39.21 (35.08, 43.56) |  |
| 30 | 35.41 (32.09, 38.89) | 2% |  | 36.06 (32.50, 39.79) | 1% |  | 36.03 (32.78, 39.44) | 3% |  | 38.04 (34.51, 41.73) | -3% |
| 40 | 35.46 (32.05, 39.03) | 0% |  | 35.91 (32.43, 39.57) | 0% |  | 36.15 (32.86, 39.60) | 0% |  | 36.73 (33.34, 40.29) | -3% |
| 50 | 34.96 (31.46, 38.64) | -1% |  | 35.22 (31.67, 38.96) | -2% |  | 35.49 (32.13, 39.01) | -2% |  | 35.31 (31.96, 38.83) | -4% |
| 60 | 33.93 (30.51, 37.54) | -3% |  | 33.99 (30.55, 37.60) | -3% |  | 34.07 (30.79, 37.52) | -4% |  | 33.77 (30.56, 37.15) | -4% |
| 70 | 32.39 (29.19, 35.76) | -5% |  | 32.25 (29.09, 35.56) | -5% |  | 31.95 (28.85, 35.21) | -6% |  | 32.13 (29.13, 35.28) | -5% |
| 80 | 30.38 (27.32, 33.61) | -6% |  | 30.04 (27.04, 33.20) | -7% |  | 29.18 (26.15, 32.38) | -9% |  | 30.39 (27.45, 33.48) | -5% |
| 90 | 27.95 (24.58, 31.54) | -8% |  | 27.43 (24.02, 31.06) | -9% |  | 25.88 (22.53, 29.46) | -11% |  | 28.57 (25.20, 32.15) | -6% |
| Average inter-decade difference* | | -4% |  |  | -4% |  |  | -5% |  |  | -5% |

*The average difference was calculated from the 6^th^ decade for gait speed, the 5^th^ decade for handgrip strength, and the 3^rd^ decade for the other outcomes. Low and high SB levels were defined using the first and third quartile values of the distribution, which roughly equal to 5.4h/d and 7.7h/d, respectively. Likewise, low and high MVPA levels were defined using the first and third quartile values of the distribution, which roughly equal to 10 min/d and 37 min/d, respectively. Note: No “between-group” comparisons could be performed as age, SB and PA were continuous variables. The reader may consult Supplementary Tables 2, 3d and 3e to obtain the age-ranges in which significant differences were observed.

Supplementary Table 7a. Estimated marginal mean values of physical function and performance across the adult lifespan according to the levels of MVPA and SB (2/2)

|  | **Low MVPA / High SB** | |  | **Low MVPA / Low SB** | |  | **High MVPA / High SB** | |  | **High MVPA / Low SB** | |
| --- | --- | --- | --- | --- | --- | --- | --- | --- | --- | --- | --- |
| **Age** | **MEAN (95% CI)** | **Inter-decade difference (%)** |  | **MEAN (95% CI)** | **Inter-decade difference (%)** |  | **MEAN (95% CI)** | **Inter-decade difference (%)** |  | **MEAN (95% CI)** | **Inter-decade difference (%)** |
| **5-time chair rise test (s)** | | | | | | | | | | | |
| 20 | 7.05 ( 5.99, 8.21) |  |  | 7.77 ( 6.61, 9.03) |  |  | 7.54 (6.48, 8.67) |  |  | 7.79 (6.69, 8.97) |  |
| 30 | 7.67 ( 6.63, 8.77) | 9% |  | 8.24 ( 7.13, 9.42) | 6% |  | 7.96 (6.95, 9.05) | 6% |  | 8.19 (7.14, 9.31) | 5% |
| 40 | 8.3 ( 7.30, 9.38) | 8% |  | 8.72 ( 7.66, 9.84) | 6% |  | 8.4 (7.41, 9.45) | 6% |  | 8.6 (7.58, 9.68) | 5% |
| 50 | 8.97 ( 7.97, 10.02) | 8% |  | 9.21 ( 8.18, 10.29) | 6% |  | 8.85 (7.86, 9.89) | 5% |  | 9.02 (8.02, 10.07) | 5% |
| 60 | 9.66 ( 8.65, 10.72) | 8% |  | 9.71 ( 8.70, 10.78) | 5% |  | 9.31 (8.30, 10.37) | 5% |  | 9.45 (8.45, 10.50) | 5% |
| 70 | 10.37 ( 9.32, 11.47) | 7% |  | 10.23 ( 9.20, 11.31) | 5% |  | 9.78 (8.73, 10.89) | 5% |  | 9.89 (8.87, 10.97) | 5% |
| 80 | 11.11 (10.00, 12.29) | 7% |  | 10.76 ( 9.69, 11.90) | 5% |  | 10.26 (9.14, 11.46) | 5% |  | 10.34 (9.27, 11.47) | 5% |
| 90 | 11.88 (10.66, 13.16) | 7% |  | 11.31 (10.15, 12.53) | 5% |  | 10.76 (9.53, 12.06) | 5% |  | 10.8 (9.66, 12.01) | 4% |
| Average inter-decade difference* | | 8% |  |  | 6% |  |  | 5% |  |  | 5% |
| **V̇O_2_max (ml/kg/min)** | | | | | | | | | | | |
| 20 | 29.54 (23.97, 35.69) |  |  | 27.05 (20.80, 34.13) |  |  | 36.77 (30.33, 43.82) |  |  | 40.4 (33.19, 48.32) |  |
| 30 | 27.99 (23.03, 33.44) | -5% |  | 26.08 (20.60, 32.20) | -4% |  | 33.94 (28.29, 40.11) | -8% |  | 36.76 (30.52, 43.58) | -9% |
| 40 | 26.49 (21.99, 31.41) | -5% |  | 25.13 (20.31, 30.46) | -4% |  | 31.23 (26.19, 36.72) | -8% |  | 33.28 (27.83, 39.21) | -9% |
| 50 | 25.04 (20.85, 29.61) | -5% |  | 24.19 (19.86, 28.95) | -4% |  | 28.64 (24.01, 33.66) | -8% |  | 29.98 (25.13, 35.25) | -10% |
| 60 | 23.62 (19.58, 28.04) | -6% |  | 23.27 (19.22, 27.70) | -4% |  | 26.15 (21.76, 30.95) | -9% |  | 26.84 (22.40, 31.69) | -10% |
| 70 | 22.24 (18.20, 26.69) | -6% |  | 22.37 (18.38, 26.75) | -4% |  | 23.78 (19.45, 28.54) | -9% |  | 23.89 (19.66, 28.52) | -11% |
| 80 | 20.91 (16.74, 25.53) | -6% |  | 21.48 (17.34, 26.07) | -4% |  | 21.52 (17.13, 26.41) | -10% |  | 21.1 (16.95, 25.71) | -12% |
| 90 | 19.61 (15.24, 24.53) | -6% |  | 20.62 (16.17, 25.60) | -4% |  | 19.37 (14.85, 24.49) | -10% |  | 18.49 (14.32, 23.19) | -12% |
| Average inter-decade difference* | | -6% |  |  | -4% |  |  | -9% |  |  | -11% |
| **Isokinetic strength (N-m)** | | | | | | | | | | | |
| 20 | 170.9 (131.20, 215.85) |  |  | 140.59 (99.64, 188.57) |  |  | 154.73 (117.02, 197.71) |  |  | 145.98 (108.02, 189.66) |  |
| 30 | 156.29 (121.63, 195.29) | -9% |  | 133.77 (98.16, 174.89) | -5% |  | 144.87 (111.44, 182.69) | -6% |  | 138.87 (105.15, 177.28) | -5% |
| 40 | 142.33 (111.79, 176.55) | -9% |  | 127.12 (96.10, 162.49) | -5% |  | 135.34 (105.35, 169.09) | -7% |  | 131.94 (101.75, 166.04) | -5% |
| 50 | 129.02 (101.62, 159.69) | -9% |  | 120.65 (93.23, 151.59) | -5% |  | 126.13 ( 98.63, 157.01) | -7% |  | 125.18 ( 97.68, 156.09) | -5% |
| 60 | 116.37 ( 91.07, 144.76) | -10% |  | 114.34 (89.31, 142.45) | -5% |  | 117.25 ( 91.25, 146.50) | -7% |  | 118.6 ( 92.82, 147.54) | -5% |
| 70 | 104.36 ( 80.24, 131.66) | -10% |  | 108.2 (84.18, 135.22) | -5% |  | 108.69 ( 83.25, 137.50) | -7% |  | 112.2 ( 87.15, 140.42) | -5% |
| 80 | 93.02 ( 69.31, 120.20) | -11% |  | 102.23 (77.93, 129.82) | -6% |  | 100.45 ( 74.82, 129.84) | -8% |  | 105.98 ( 80.75, 134.63) | -6% |
| 90 | 82.32 ( 58.56, 110.12) | -12% |  | 96.43 (70.83, 125.96) | -6% |  | 92.54 ( 66.20, 123.28) | -8% |  | 99.93 ( 73.82, 129.99) | -6% |
| Average inter-decade difference* | | -10% |  |  | -5% |  |  | -7% |  |  | -5% |

*The average difference was calculated from the 6^th^ decade for gait speed, the 5^th^ decade for handgrip strength, and the 3^rd^ decade for the other outcomes. Low and high SB levels were defined using the first and third quartile values of the distribution, which roughly equal to 5.4h/d and 7.7h/d, respectively. Likewise, low and high MVPA levels were defined using the first and third quartile values of the distribution, which roughly equal to 10 min/d and 37 min/d, respectively. Note: No “between-group” comparisons could be performed as age, SB and PA were continuous variables. The reader may consult Supplementary Tables 2, 3d and 3e to obtain the age-ranges in which significant differences were observed.

Supplementary Table 7b. Estimated marginal mean values of physical function and performance across the adult lifespan according to the levels of MVPA and SB in men (1/2)

|  | **Low MVPA / High SB** | |  | **Low MVPA / Low SB** | |  | **High MVPA / High SB** | |  | **High MVPA / Low SB** | |
| --- | --- | --- | --- | --- | --- | --- | --- | --- | --- | --- | --- |
| **Age** | **MEAN (95% CI)** | **Inter-decade difference (%)** |  | **MEAN (95% CI)** | **Inter-decade difference (%)** |  | **MEAN (95% CI)** | **Inter-decade difference (%)** |  | **MEAN (95% CI)** | **Inter-decade difference (%)** |
| **Gait speed (m/s)** | | | | | | | | | | | |
| 20 | 1.25 (1.01, 1.49) |  |  | 1.24 (0.97, 1.50) |  |  | 1.38 (1.16, 1.60) |  |  | 1.24 (0.98, 1.49) |  |
| 30 | 1.3 (1.12, 1.49) | 4% |  | 1.3 (1.11, 1.50) | 5% |  | 1.36 (1.18, 1.53) | -1% |  | 1.27 (1.07, 1.47) | 2% |
| 40 | 1.33 (1.15, 1.51) | 2% |  | 1.34 (1.15, 1.53) | 3% |  | 1.33 (1.16, 1.51) | -2% |  | 1.3 (1.12, 1.48) | 2% |
| 50 | 1.34 (1.16, 1.52) | 1% |  | 1.36 (1.17, 1.54) | 1% |  | 1.32 (1.14, 1.49) | -1% |  | 1.32 (1.14, 1.49) | 2% |
| 60 | 1.32 (1.14, 1.49) | -1% |  | 1.34 (1.16, 1.52) | -1% |  | 1.3 (1.13, 1.48) | -2% |  | 1.32 (1.15, 1.50) | 0% |
| 70 | 1.27 (1.11, 1.44) | -4% |  | 1.3 (1.14, 1.47) | -3% |  | 1.29 (1.12, 1.46) | -1% |  | 1.33 (1.16, 1.49) | 1% |
| 80 | 1.21 (1.03, 1.38) | -5% |  | 1.24 (1.07, 1.41) | -5% |  | 1.28 (1.10, 1.46) | -1% |  | 1.32 (1.15, 1.49) | -1% |
| 90 | 1.11 (0.90, 1.33) | -8% |  | 1.14 (0.91, 1.37) | -8% |  | 1.28 (1.05, 1.51) | 0% |  | 1.3 (1.09, 1.51) | -2% |
| Average inter-decade difference* | | -5% |  |  | -4% |  |  | -1% |  |  | 0% |
| **Handgrip strength (kg)** | | | | | | | | | | | |
| 20 | 45.83 (37.02, 55.57) |  |  | 46.55 (36.81, 57.43) |  |  | 44.75 (36.16, 54.26) |  |  | 46.4 (36.34, 57.69) |  |
| 30 | 47.27 (39.64, 55.57) | 3% |  | 48.7 (40.62, 57.51) | 5% |  | 46.38 (39.17, 54.19) | 4% |  | 47.27 (39.24, 56.04) | 2% |
| 40 | 47.76 (40.42, 55.72) | 1% |  | 49.53 (41.71, 58.02) | 2% |  | 47.04 (40.02, 54.63) | 1% |  | 47.39 (40.10, 55.29) | 0% |
| 50 | 47.29 (39.91, 55.28) | -1% |  | 49 (41.20, 57.46) | -1% |  | 46.72 (39.58, 54.46) | -1% |  | 46.78 (39.66, 54.48) | -1% |
| 60 | 45.85 (38.71, 53.59) | -3% |  | 47.13 (39.78, 55.09) | -4% |  | 45.43 (38.44, 53.00) | -3% |  | 45.43 (38.58, 52.84) | -3% |
| 70 | 43.51 (36.87, 50.70) | -5% |  | 44 (37.43, 51.10) | -7% |  | 43.21 (36.58, 50.39) | -5% |  | 43.39 (36.97, 50.32) | -4% |
| 80 | 40.33 (33.83, 47.39) | -7% |  | 39.75 (33.35, 46.71) | -10% |  | 40.13 (33.40, 47.47) | -7% |  | 40.7 (34.35, 47.59) | -6% |
| 90 | 36.42 (28.87, 44.83) | -10% |  | 34.56 (26.73, 43.41) | -13% |  | 36.29 (28.27, 45.30) | -10% |  | 37.43 (29.94, 45.75) | -8% |
| Average inter-decade difference* | | -4% |  |  | -5% |  |  | -4% |  |  | -4% |

*The average difference was calculated from the 6^th^ decade for gait speed, the 5^th^ decade for handgrip strength, and the 3^rd^ decade for the other outcomes. Low and high SB levels were defined using the first and third quartile values of the distribution, which roughly equal to 5.6h/d and 8.4h/d, respectively. Likewise, low and high MVPA levels were defined using the first and third quartile values of the distribution, which roughly equal to 11 min/d and 39 min/d, respectively. Note: No “between-group” comparisons could be performed as age, SB and PA were continuous variables. The reader may consult Supplementary Tables 2, 3d and 3e to obtain the age-ranges in which significant differences were observed.

Supplementary Table 7b. Estimated marginal mean values of physical function and performance across the adult lifespan according to the levels of MVPA and SB in men (2/2)

|  | **Low MVPA / High SB** | |  | **Low MVPA / Low SB** | |  | **High MVPA / High SB** | |  | **High MVPA / Low SB** | |
| --- | --- | --- | --- | --- | --- | --- | --- | --- | --- | --- | --- |
| **Age** | **MEAN (95% CI)** | **Inter-decade difference (%)** |  | **MEAN (95% CI)** | **Inter-decade difference (%)** |  | **MEAN (95% CI)** | **Inter-decade difference (%)** |  | **MEAN (95% CI)** | **Inter-decade difference (%)** |
| **5-time chair rise test (s)** | | | | | | | | | | | |
| 20 | 6.46 (4.86, 8.29) |  |  | 7.3 (5.50, 9.36) |  |  | 6.83 (5.23, 8.65) |  |  | 8.61 (6.80, 10.63) |  |
| 30 | 7.05 (5.52, 8.77) | 9% |  | 7.74 (6.06, 9.64) | 6% |  | 7.24 (5.73, 8.91) | 6% |  | 8.74 (7.06, 10.60) | 2% |
| 40 | 7.67 (6.21, 9.29) | 9% |  | 8.2 (6.62, 9.95) | 6% |  | 7.65 (6.22, 9.23) | 6% |  | 8.88 (7.31, 10.60) | 2% |
| 50 | 8.32 (6.89, 9.87) | 8% |  | 8.67 (7.17, 10.31) | 6% |  | 8.08 (6.68, 9.61) | 6% |  | 9.01 (7.52, 10.64) | 1% |
| 60 | 8.99 (7.56, 10.53) | 8% |  | 9.15 (7.69, 10.73) | 6% |  | 8.52 (7.09, 10.07) | 5% |  | 9.15 (7.69, 10.73) | 2% |
| 70 | 9.68 (8.21, 11.28) | 8% |  | 9.64 (8.17, 11.23) | 5% |  | 8.97 (7.46, 10.62) | 5% |  | 9.29 (7.83, 10.88) | 2% |
| 80 | 10.41 (8.82, 12.13) | 8% |  | 10.15 (8.61, 11.81) | 5% |  | 9.43 (7.77, 11.25) | 5% |  | 9.43 (7.91, 11.08) | 2% |
| 90 | 11.16 (9.39, 13.07) | 7% |  | 10.67 (9.00, 12.48) | 5% |  | 9.9 (8.04, 11.96) | 5% |  | 9.57 (7.96, 11.33) | 1% |
| Average inter-decade difference* | | 8% |  |  | 6% |  |  | 5% |  |  | 2% |
| **V̇O_2_max (ml/kg/min)** | | | | | | | | | | | |
| 20 | 32.74 (25.93, 40.34) |  |  | 28.94 (21.78, 37.11) |  |  | 40.27 (32.46, 48.92) |  |  | 43.67 (35.63, 52.53) |  |
| 30 | 30.76 (24.79, 37.38) | -6% |  | 27.7 (21.46, 34.74) | -4% |  | 36.78 (30.03, 44.22) | -9% |  | 39.59 (32.59, 47.28) | -9% |
| 40 | 28.85 (23.51, 34.73) | -6% |  | 26.49 (21.01, 32.61) | -4% |  | 33.45 (27.51, 39.97) | -9% |  | 35.72 (29.56, 42.46) | -10% |
| 50 | 26.99 (22.08, 32.40) | -6% |  | 25.31 (20.38, 30.77) | -4% |  | 30.28 (24.89, 36.19) | -9% |  | 32.04 (26.52, 38.09) | -10% |
| 60 | 25.2 (20.48, 30.41) | -7% |  | 24.15 (19.55, 29.24) | -5% |  | 27.26 (22.17, 32.88) | -10% |  | 28.56 (23.47, 34.16) | -11% |
| 70 | 23.47 (18.73, 28.74) | -7% |  | 23.02 (18.49, 28.06) | -5% |  | 24.41 (19.39, 30.00) | -10% |  | 25.29 (20.44, 30.65) | -11% |
| 80 | 21.8 (16.89, 27.34) | -7% |  | 21.92 (17.22, 27.19) | -5% |  | 21.71 (16.62, 27.47) | -11% |  | 22.21 (17.47, 27.52) | -12% |
| 90 | 20.19 (15.01, 26.15) | -7% |  | 20.85 (15.82, 26.57) | -5% |  | 19.17 (13.93, 25.23) | -12% |  | 19.33 (14.61, 24.72) | -13% |
| Average inter-decade difference* | | -7% |  |  | -5% |  |  | -10% |  |  | -11% |
| **Isokinetic strength (N-m)** | | | | | | | | | | | |
| 20 | 170.6 (97.63, 263.81) |  |  | 136.69 (70.37, 224.82) |  |  | 130.51 (69.99, 209.73) |  |  | 137.32 (73.75, 220.49) |  |
| 30 | 155.41 (92.39, 234.74) | -9% |  | 128.54 (70.48, 203.92) | -6% |  | 124.71 (70.13, 194.90) | -4% |  | 129.79 (73.30, 202.31) | -5% |
| 40 | 140.93 (86.47, 208.63) | -9% |  | 120.65 (69.74, 185.43) | -6% |  | 119.04 (69.33, 182.10) | -5% |  | 122.47 (71.79, 186.61) | -6% |
| 50 | 127.16 (79.67, 185.69) | -10% |  | 113.01 (67.83, 169.65) | -6% |  | 113.51 (67.39, 171.58) | -5% |  | 115.37 (68.95, 173.67) | -6% |
| 60 | 114.09 (71.82, 166.09) | -10% |  | 105.61 (64.44, 156.90) | -7% |  | 108.1 (64.14, 163.46) | -5% |  | 108.48 (64.60, 163.67) | -6% |
| 70 | 101.73 (62.88, 149.89) | -11% |  | 98.47 (59.40, 147.36) | -7% |  | 102.83 (59.61, 157.75) | -5% |  | 101.8 (58.78, 156.56) | -6% |
| 80 | 90.08 (53.06, 136.85) | -11% |  | 91.58 (52.86, 140.87) | -7% |  | 97.69 (53.99, 154.24) | -5% |  | 95.34 (51.81, 152.02) | -6% |
| 90 | 79.14 (42.84, 126.50) | -12% |  | 84.93 (45.27, 136.97) | -7% |  | 92.68 (47.62, 152.60) | -5% |  | 89.08 (44.17, 149.59) | -7% |
| Average inter-decade difference* | | -10% |  |  | -7% |  |  | -5% |  |  | -6% |

*The average difference was calculated from the 6^th^ decade for gait speed, the 5^th^ decade for handgrip strength, and the 3^rd^ decade for the other outcomes. Low and high SB levels were defined using the first and third quartile values of the distribution, which roughly equal to 5.6h/d and 8.4h/d, respectively. Likewise, low and high MVPA levels were defined using the first and third quartile values of the distribution, which roughly equal to 11 min/d and 39 min/d, respectively. Note: No “between-group” comparisons could be performed as age, SB and PA were continuous variables. The reader may consult Supplementary Tables 2, 3d and 3e to obtain the age-ranges in which significant differences were observed.

Supplementary Table 7c. Estimated marginal mean values of physical function and performance across the adult lifespan according to the levels of MVPA and SB in women (1/2)

|  | **Low MVPA / High SB** | |  | **Low MVPA / Low SB** | |  | **High MVPA / High SB** | |  | **High MVPA / Low SB** | |
| --- | --- | --- | --- | --- | --- | --- | --- | --- | --- | --- | --- |
| **Age** | **MEAN (95% CI)** | **Inter-decade difference (%)** |  | **MEAN (95% CI)** | **Inter-decade difference (%)** |  | **MEAN (95% CI)** | **Inter-decade difference (%)** |  | **MEAN (95% CI)** | **Inter-decade difference (%)** |
| **Gait speed (m/s)** | | | | | | | | | | | |
| 20 | 1.02 (0.84, 1.21) |  |  | 1.04 (0.85, 1.23) |  |  | 1.07 (0.93, 1.20) |  |  | 1.06 (0.93, 1.20) |  |
| 30 | 1.08 (0.97, 1.19) | 6% |  | 1.11 (0.94, 1.29) | 7% |  | 1.11 (0.99, 1.23) | 4% |  | 1.12 (1.01, 1.24) | 6% |
| 40 | 1.11 (1.00, 1.23) | 3% |  | 1.16 (1.04, 1.28) | 5% |  | 1.13 (1.02, 1.25) | 2% |  | 1.16 (1.04, 1.27) | 4% |
| 50 | 1.13 (1.01, 1.25) | 2% |  | 1.18 (1.05, 1.30) | 2% |  | 1.14 (1.02, 1.26) | 1% |  | 1.17 (1.05, 1.28) | 1% |
| 60 | 1.12 (1.00, 1.24) | -1% |  | 1.17 (1.04, 1.29) | -1% |  | 1.13 (1.01, 1.24) | -1% |  | 1.15 (1.04, 1.27) | -2% |
| 70 | 1.09 (0.97, 1.21) | -3% |  | 1.12 (1.01, 1.24) | -4% |  | 1.1 (0.98, 1.21) | -3% |  | 1.11 (1.00, 1.23) | -3% |
| 80 | 1.04 (0.91, 1.16) | -5% |  | 1.05 (0.93, 1.17) | -6% |  | 1.05 (0.92, 1.17) | -5% |  | 1.05 (0.93, 1.17) | -5% |
| 90 | 0.97 (0.81, 1.12) | -7% |  | 0.95 (0.80, 1.10) | -10% |  | 0.98 (0.82, 1.13) | -7% |  | 0.97 (0.81, 1.13) | -8% |
| Average inter-decade difference* | | -4% |  |  | -5% |  |  | -4% |  |  | -5% |
| **Handgrip strength (kg)** | | | | | | | | | | | |
| 20 | 27.64 (23.39, 32.24) |  |  | 28.65 (24.06, 33.64) |  |  | 28.19 (24.72, 31.88) |  |  | 32.08 (28.27, 36.14) |  |
| 30 | 27.82 (24.96, 30.84) | 1% |  | 28.65 (24.75, 32.84) | 0% |  | 28.95 (25.90, 32.17) | 3% |  | 30.78 (27.63, 34.11) | -4% |
| 40 | 27.7 (24.74, 30.82) | 0% |  | 28.27 (25.20, 31.52) | -1% |  | 28.98 (25.96, 32.17) | 0% |  | 29.44 (26.38, 32.67) | -4% |
| 50 | 27.27 (24.18, 30.55) | -2% |  | 27.52 (24.32, 30.92) | -3% |  | 28.28 (25.24, 31.49) | -2% |  | 28.07 (25.01, 31.30) | -5% |
| 60 | 26.54 (23.47, 29.80) | -3% |  | 26.41 (23.28, 29.74) | -4% |  | 26.87 (23.92, 29.99) | -5% |  | 26.66 (23.74, 29.75) | -5% |
| 70 | 25.52 (22.57, 28.66) | -4% |  | 24.97 (22.07, 28.04) | -5% |  | 24.81 (22.02, 27.76) | -8% |  | 25.23 (22.49, 28.13) | -5% |
| 80 | 24.24 (21.22, 27.46) | -5% |  | 23.21 (20.42, 26.18) | -7% |  | 22.18 (19.39, 25.17) | -11% |  | 23.78 (20.92, 26.83) | -6% |
| 90 | 22.7 (19.11, 26.61) | -6% |  | 21.2 (17.95, 24.72) | -9% |  | 19.1 (15.90, 22.60) | -14% |  | 22.32 (18.67, 26.30) | -6% |
| Average inter-decade difference* | | -3% |  |  | -5% |  |  | -7% |  |  | -5% |

*The average difference was calculated from the 6^th^ decade for gait speed, the 5^th^ decade for handgrip strength, and the 3^rd^ decade for the other outcomes. Low and high SB levels were defined using the first and third quartile values of the distribution, which roughly equal to 5.3h/d and 7.5h/d, respectively. Likewise, low and high MVPA levels were defined using the first and third quartile values of the distribution, which roughly equal to 10 min/d and 36 min/d, respectively. Note: No “between-group” comparisons could be performed as age, SB and PA were continuous variables. The reader may consult Supplementary Tables 2, 3d and 3e to obtain the age-ranges in which significant differences were observed.

Supplementary Table 7c. Estimated marginal mean values of physical function and performance across the adult lifespan according to the levels of MVPA and SB in women (2/2)

|  | **Low MVPA / High SB** | |  | **Low MVPA / Low SB** | |  | **High MVPA / High SB** | |  | **High MVPA / Low SB** | |
| --- | --- | --- | --- | --- | --- | --- | --- | --- | --- | --- | --- |
| **Age** | **MEAN (95% CI)** | **Inter-decade difference (%)** |  | **MEAN (95% CI)** | **Inter-decade difference (%)** |  | **MEAN (95% CI)** | **Inter-decade difference (%)** |  | **MEAN (95% CI)** | **Inter-decade difference (%)** |
| **5-time chair rise test (s)** | | | | | | | | | | | |
| 20 | 6.84 ( 5.48, 8.35) |  |  | 7.45 (5.98, 9.08) |  |  | 7.61 (6.20, 9.15) |  |  | 7.08 (5.71, 8.61) |  |
| 30 | 7.47 ( 6.13, 8.94) | 9% |  | 7.91 (6.49, 9.47) | 6% |  | 7.99 (6.62, 9.50) | 5% |  | 7.6 (6.25, 9.08) | 7% |
| 40 | 8.13 ( 6.78, 9.59) | 9% |  | 8.38 (7.00, 9.89) | 6% |  | 8.39 (7.03, 9.87) | 5% |  | 8.14 (6.80, 9.59) | 7% |
| 50 | 8.81 ( 7.45, 10.28) | 8% |  | 8.87 (7.50, 10.35) | 6% |  | 8.8 (7.43, 10.28) | 5% |  | 8.69 (7.35, 10.14) | 7% |
| 60 | 9.52 ( 8.11, 11.04) | 8% |  | 9.37 (7.99, 10.85) | 6% |  | 9.21 (7.81, 10.73) | 5% |  | 9.27 (7.90, 10.74) | 7% |
| 70 | 10.26 ( 8.77, 11.86) | 8% |  | 9.88 (8.46, 11.41) | 5% |  | 9.64 (8.17, 11.23) | 5% |  | 9.86 (8.44, 11.38) | 6% |
| 80 | 11.03 ( 9.43, 12.75) | 8% |  | 10.41 (8.91, 12.01) | 5% |  | 10.08 (8.52, 11.77) | 5% |  | 10.47 (8.97, 12.08) | 6% |
| 90 | 11.82 (10.08, 13.70) | 7% |  | 10.95 (9.35, 12.67) | 5% |  | 10.52 (8.85, 12.34) | 4% |  | 11.09 (9.49, 12.82) | 6% |
| Average inter-decade difference* | | 8% |  |  | 6% |  |  | 5% |  |  | 7% |
| **V̇O_2_max (ml/kg/min)** | | | | | | | | | | | |
| 20 | 33.36 (27.02, 40.37) |  |  | 30.93 (23.65, 39.18) |  |  | 41.19 (33.81, 49.30) |  |  | 44.41 (35.70, 54.07) |  |
| 30 | 31.27 (25.57, 37.54) | -6% |  | 29.34 (22.97, 36.48) | -5% |  | 37.55 (31.05, 44.66) | -9% |  | 40.2 (32.74, 48.44) | -9% |
| 40 | 29.25 (24.05, 34.95) | -6% |  | 27.79 (22.18, 34.03) | -5% |  | 34.07 (28.26, 40.41) | -9% |  | 36.21 (29.76, 43.29) | -10% |
| 50 | 27.29 (22.45, 32.61) | -7% |  | 26.28 (21.24, 31.86) | -5% |  | 30.76 (25.44, 36.58) | -10% |  | 32.42 (26.74, 38.65) | -10% |
| 60 | 25.41 (20.75, 30.54) | -7% |  | 24.82 (20.13, 30.00) | -6% |  | 27.62 (22.59, 33.15) | -10% |  | 28.84 (23.67, 34.53) | -11% |
| 70 | 23.59 (18.98, 28.69) | -7% |  | 23.4 (18.82, 28.47) | -6% |  | 24.65 (19.75, 30.09) | -11% |  | 25.48 (20.57, 30.91) | -12% |
| 80 | 21.84 (17.17, 27.06) | -7% |  | 22.02 (17.36, 27.23) | -6% |  | 21.84 (16.96, 27.34) | -11% |  | 22.32 (17.49, 27.72) | -12% |
| 90 | 20.15 (15.36, 25.60) | -8% |  | 20.68 (15.78, 26.23) | -6% |  | 19.21 (14.28, 24.87) | -12% |  | 19.37 (14.52, 24.91) | -13% |
| Average inter-decade difference* | | -7% |  |  | -6% |  |  | -10% |  |  | -11% |
| **Isokinetic strength (N-m)** | | | | | | | | | | | |
| 20 | 134.57 (90.03, 188.04) |  |  | 109.91 (63.01, 169.77) |  |  | 136.51 (89.20, 193.84) |  |  | 122.38 (75.81, 180.05) |  |
| 30 | 124.3 (84.11, 172.31) | -8% |  | 106.05 (64.25, 158.27) | -4% |  | 124.93 (82.88, 175.57) | -8% |  | 115.02 (73.28, 166.13) | -6% |
| 40 | 114.43 (77.39, 158.70) | -8% |  | 102.27 (64.67, 148.44) | -4% |  | 113.86 (75.86, 159.54) | -9% |  | 107.89 (70.13, 153.76) | -6% |
| 50 | 104.97 (69.86, 147.21) | -8% |  | 98.55 (63.96, 140.57) | -4% |  | 103.3 (68.10, 145.80) | -9% |  | 100.99 (66.23, 143.05) | -6% |
| 60 | 95.92 (61.68, 137.70) | -9% |  | 94.9 (61.92, 134.89) | -4% |  | 93.25 (59.71, 134.24) | -10% |  | 94.32 (61.54, 134.06) | -7% |
| 70 | 87.28 (53.12, 129.87) | -9% |  | 91.32 (58.47, 131.46) | -4% |  | 83.72 (50.93, 124.62) | -10% |  | 87.87 (56.09, 126.75) | -7% |
| 80 | 79.04 (44.52, 123.42) | -9% |  | 87.8 (53.82, 130.06) | -4% |  | 74.71 (42.09, 116.61) | -11% |  | 81.65 (50.04, 120.96) | -7% |
| 90 | 71.22 (36.18, 118.01) | -10% |  | 84.36 (48.34, 130.34) | -4% |  | 66.2 (33.54, 109.87) | -11% |  | 75.67 (43.63, 116.46) | -7% |
| Average inter-decade difference* | | -9% |  |  | -4% |  |  | -10% |  |  | -7% |

*The average difference was calculated from the 6^th^ decade for gait speed, the 5^th^ decade for handgrip strength, and the 3^rd^ decade for the other outcomes. Low and high SB levels were defined using the first and third quartile values of the distribution, which roughly equal to 5.3h/d and 7.5h/d, respectively. Likewise, low and high MVPA levels were defined using the first and third quartile values of the distribution, which roughly equal to 10 min/d and 36 min/d, respectively. Note: No “between-group” comparisons could be performed as age, SB and PA were continuous variables. The reader may consult Supplementary Tables 2, 3d and 3e to obtain the age-ranges in which significant differences were observed.


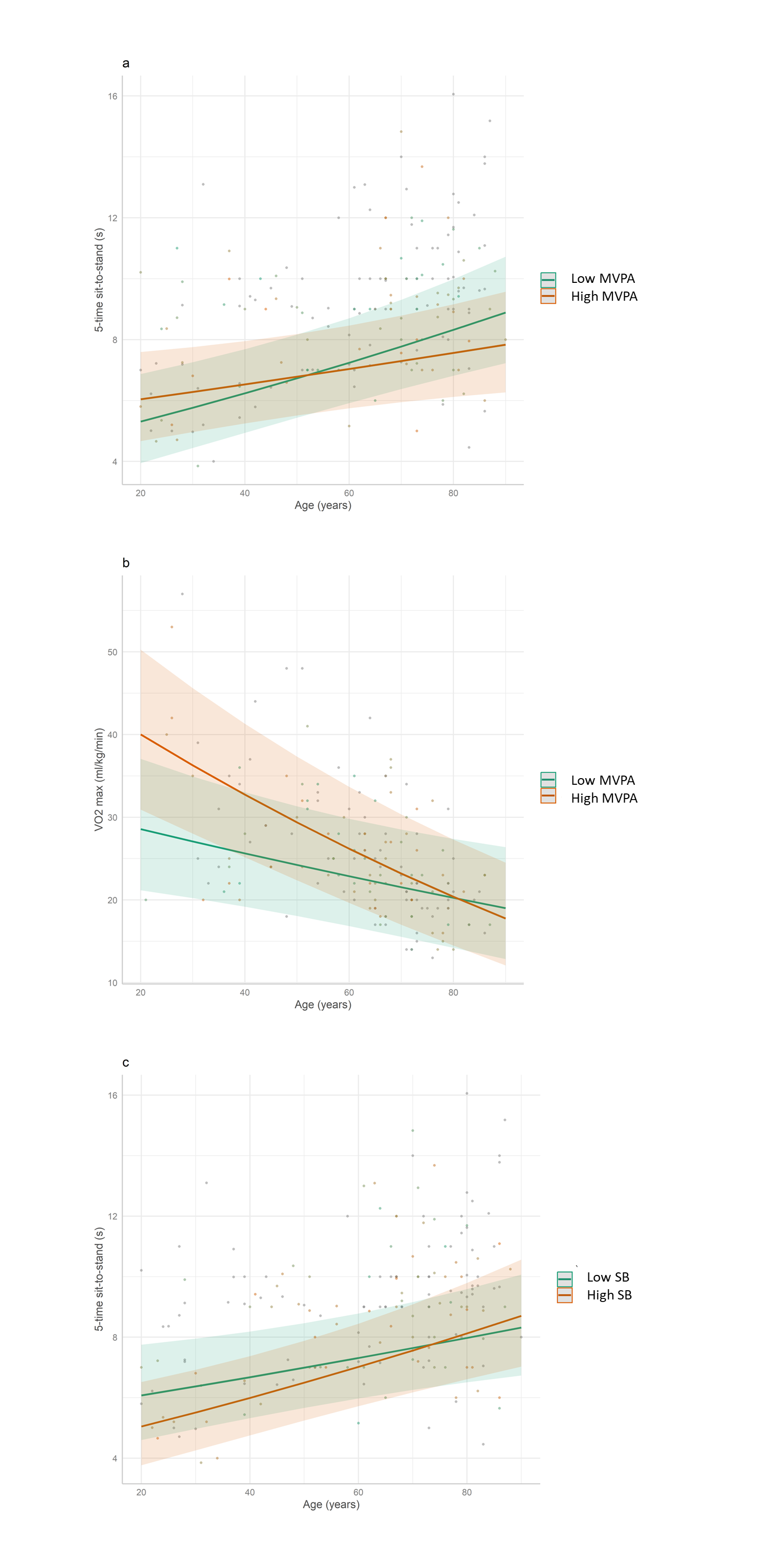


**Supplementary Figure 1. Associations between age and physical function and performance according to the levels of MVPA and SB in men.**

The graph indicates that higher MVPA levels were significantly associated with a more favourable age-related relationship in the 5-time chair rise test performance (a) and VO_2_max (b), independently of SB levels. In addition, the age-related reduction in chair rise performance was significantly more pronounced with higher levels of SB (c), independently of MVPA. For illustrative purposes, low and high levels of MVPA and SB were defined using the first and third quartile values of the distribution, which roughly equal to 11min/d and 39min/d for MVPA, and 5.6h/d and 8.4h/d for SB.


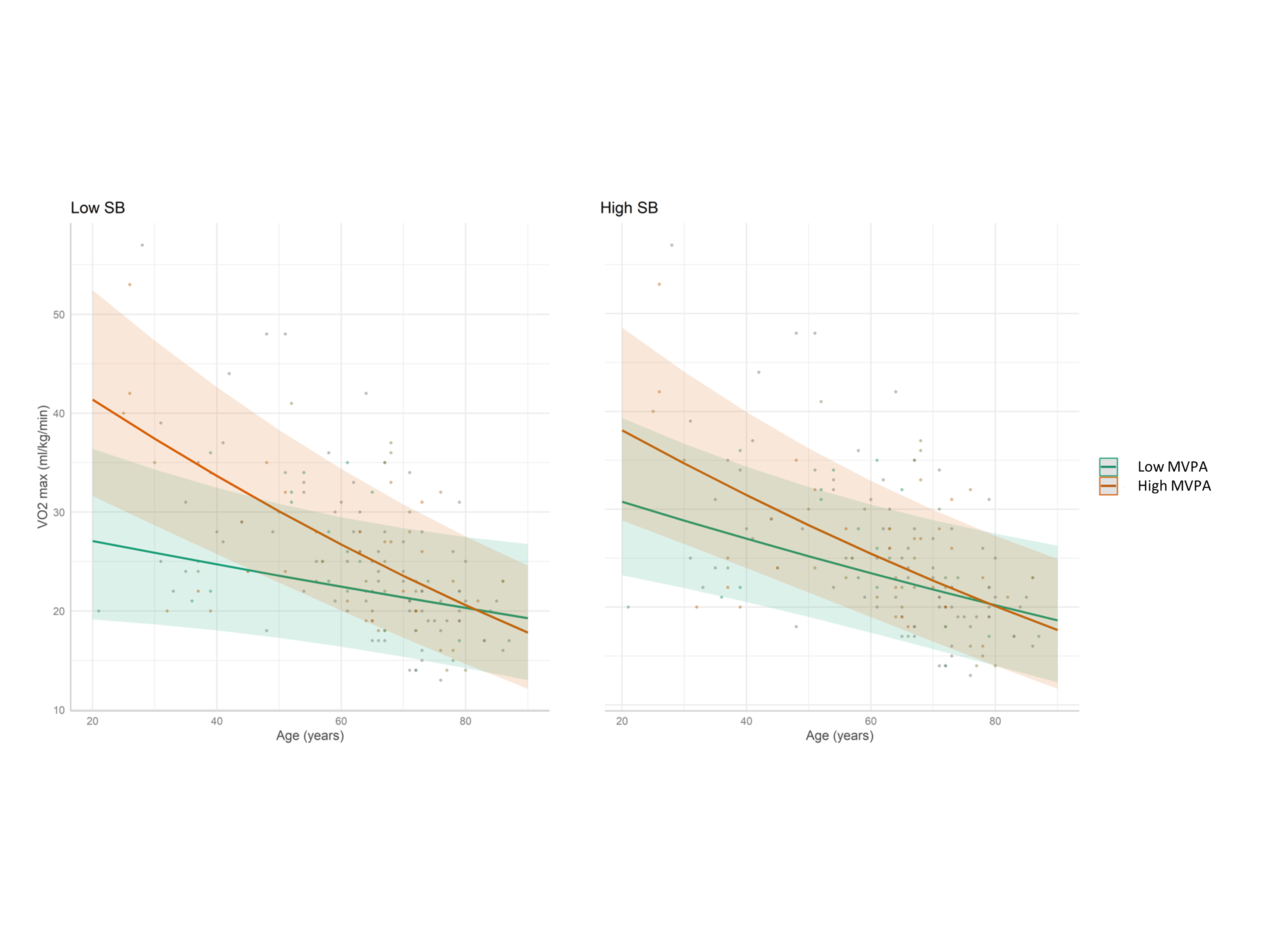


**Supplementary Figure 2. Associations between age and VO_2_max according to the levels of MVPA and SB in men.**

The graphs indicate that there was a significant three-way interaction between age, MVPA and SB such that the beneficial relationship between MVPA and VO_2_max was attenuated with higher levels of SB. For illustrative purposes, low and high levels of MVPA and SB were defined using the first and third quartile values of the distribution, which roughly equal to 11min/d and 39min/d for MVPA, and 5.6h/d and 8.4h/d for SB.


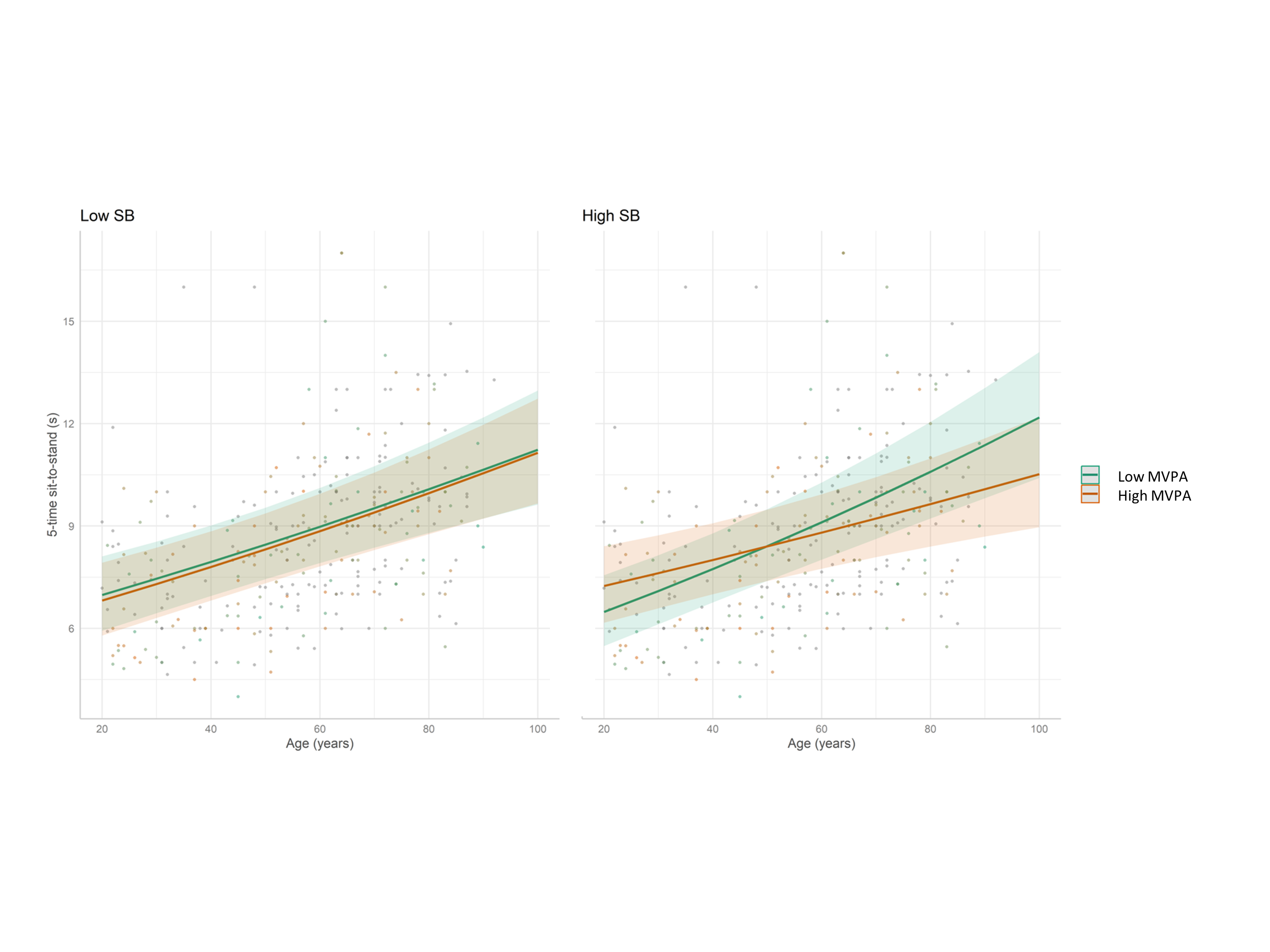


**Supplementary Figure 3. Associations between age and 5-time chair rise performance according to the levels of MVPA and SB in women.**

The graphs indicate that there was a significant three-way interaction between age, MVPA and SB, such that the age-related loss in chair rise performance was attenuated in women who combined low levels of SB with high levels of MVPA, compared to those with high SB levels and/or low levels of MVPA. For illustrative purposes, low and high levels of MVPA and SB were defined using the first and third quartile values of the distribution, which roughly equal to 10min/d and 36min/d for MVPA, and 5.3h/d and 7.5h/d for SB.


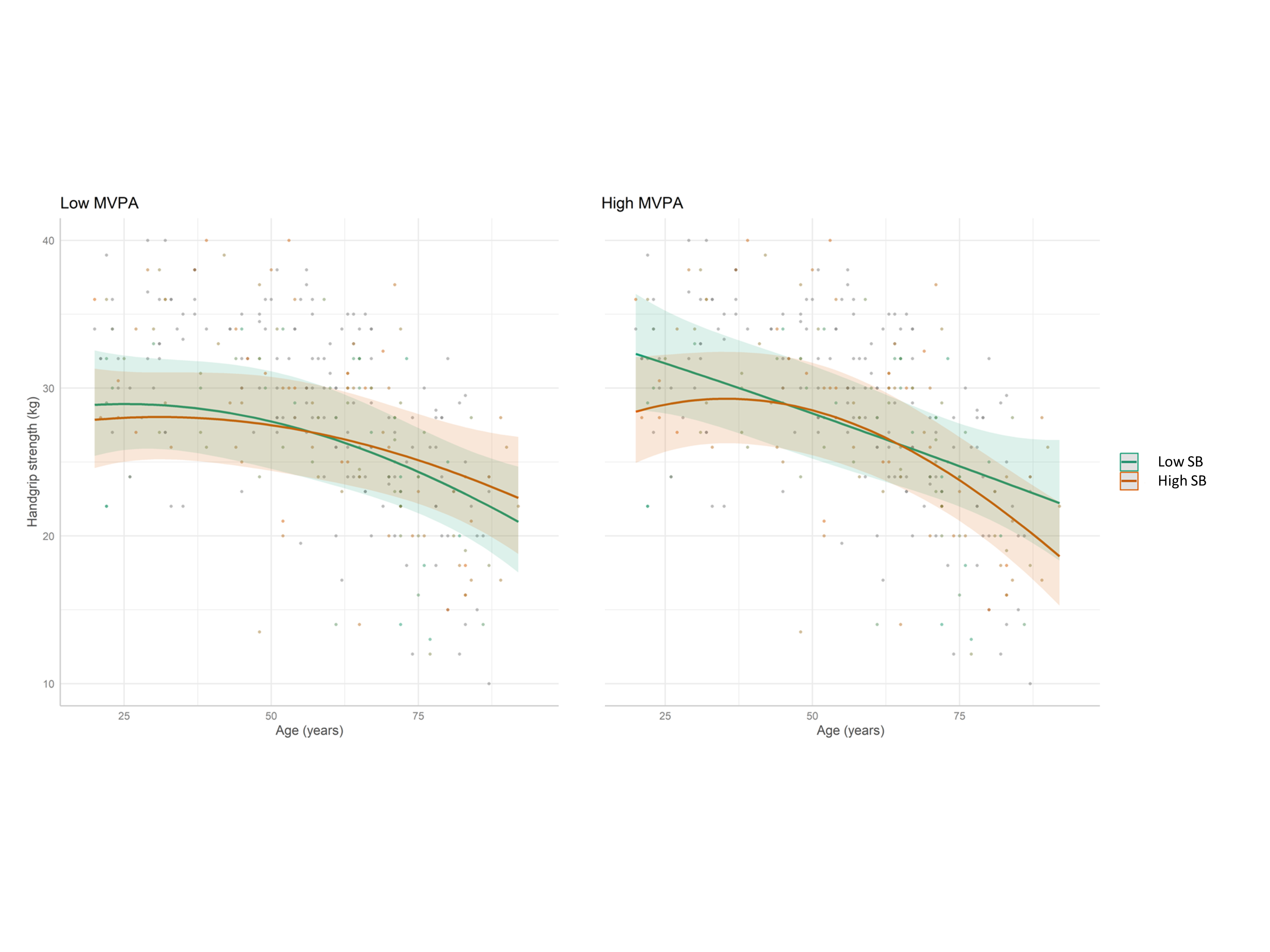


**Supplementary Figure 4. Associations between age and handgrip strength according to the levels of MVPA and SB in women.**

The graphs indicate that there was a significant three-way interaction between age, MVPA and SB, such that the age-related loss in handgrip strength was attenuated in women who combined low levels of SB with high levels of MVPA, compared to those with high SB levels and/or low levels of MVPA. For illustrative purposes, low and high levels of MVPA and SB were defined using the first and third quartile values of the distribution, which roughly equal to 10min/d and 36min/d for MVPA, and 5.4h/d and 7.4h/d for SB.
